# Supplementary material for: Regulation of developmental gatekeeping and cell fate transition by the calpain protease DEK1 in Physcomitrium patens
Source: Commun Biol. 2024 Mar 4;7:261. doi: 10.1038/s42003-024-05933-z (PMC10912778; doi:10.1038/s42003-024-05933-z)
Supplement: Supplementary file 2 — Supplementary Material [file 42003_2024_5933_MOESM2_ESM.pdf]

Supplementary Materials for

Regulation of developmental gatekeeping and cell fate transition by the calpain protease DEK1 in *Physcomitrium patens*

Viktor Demko, Tatiana Belova, Maxim Messerer, Torgeir R. Hvidsten, Pierre-François Perroud, Ako Eugene Ako, Wenche Johansen, Klaus F.X. Mayer, Odd-Arne Olsen, Daniel Lang*

* Corresponding author daniel.lang@mailbox.org

This PDF file includes:

- Supplementary Figures S1 to S13
- Description of Supplementary Data sheets S1 to S13
- Supplementary References

All other biological and software resources cited in the main text and this document including the External Files deposited in Zenodo archive 10.5281/zenodo.5513495 are listed in Supplementary Data sheet S13.

Supplementary Figures

S1

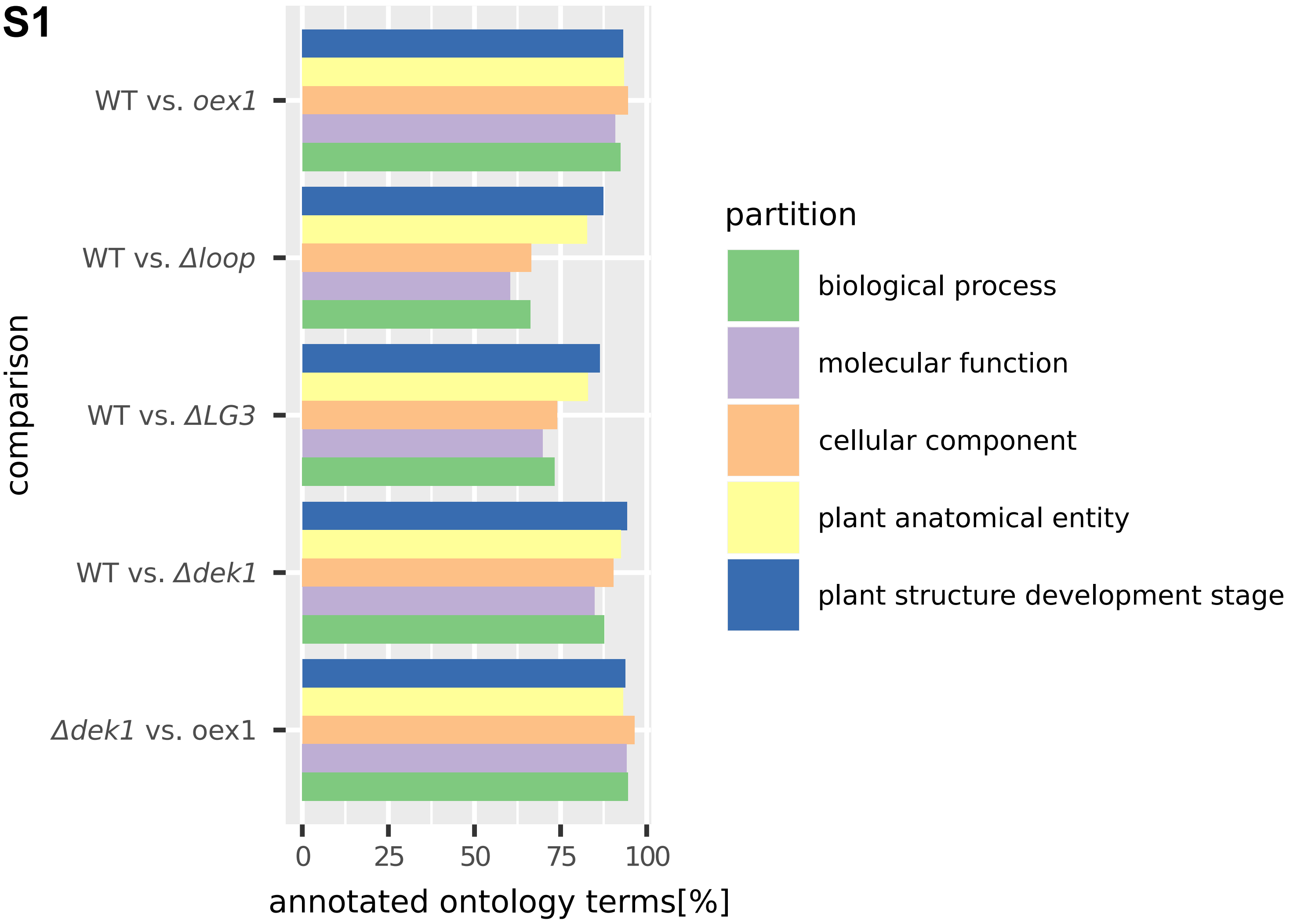

WT vs. *oex1*
WT vs. *Δloop*
WT vs. *ΔLG3*
WT vs. *Δdek1*
Δdek1 vs. oex1
comparison
0
25
50
75
100
annotated ontology terms[%]
partition
biological process
molecular function
cellular component
plant anatomical entity
plant structure development stage

Supplementary Fig. S1.

Percentage of ontology terms in deregulated genes indicates that DEK1 mutation affects most cellular and organismic functions. Percentage of unique ontology terms from the Gene Ontology *biological process*, *molecular function*, *cellular component* partitions and the Plant Ontology *plant anatomical entity* and *plant structure development stage* partitions that are assigned to deregulated genes in the mutant lines.

S2

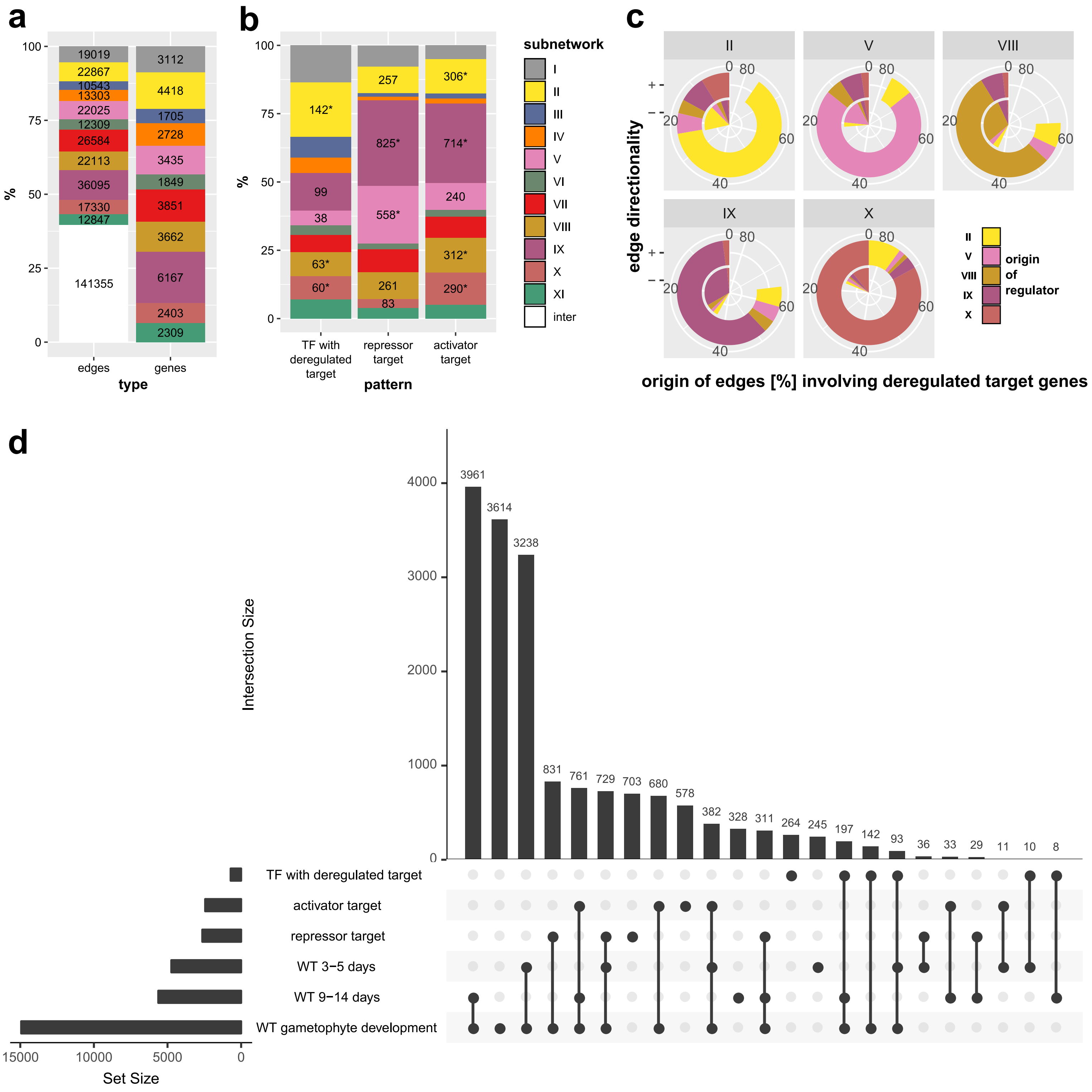

a
b
c
d
subnetwork
I
II
III
IV
V
VI
VII
VIII
IX
X
XI
inter
edges
genes
type
TF with deregulated target
repressor target
activator target
pattern
edge directionality
origin of regulator
origin of edges [%] involving deregulated target genes
Intersection Size
Set Size
TF with deregulated target
activator target
repressor target
WT 3−5 days
WT 9−14 days
WT gametophyte development

Supplementary Fig. S2.

Tracing DEK1 impact on the gene regulatory networks (GRN) of *Physcomitrium patens.*

S2a. Summary of the predicted *P. patens* GRN. Bar plot depicts the percentage of total edges (regulatory interactions) and nodes (genes) comprising the 11 subnetworks that form the *P. patens* GRN. Absolute numbers are given as bar stack annotations. Inter-subnetwork edges are displayed in white.

S2b. Network enrichment analysis of DEK1-deregulated genes. Bar plot displaying the major patterns of deregulation in *DEK1* mutants (middle and right bar) as well as upstream transcription factors (left bar) predicted to directly target the affected genes across the 11 subnetworks. Numeric annotations in bar stacks are provided for subnetworks with significant (FDR < 0.01; network enrichment analysis) enrichment of genes with a mutant deregulation pattern that is consistent with being a target of a DEK1-regulated activator or repressor (see Fig. 1e and 1f) and depicted in the network graph of Fig. 2b. An asterisk indicates individual, significant data points.

S2c. Inter- and intra-connections between enriched subnetworks. Pie chart depicting the overall percentage of edges within and between the enriched subnetworks (Fig. 2b and Supplementary Fig. S2b) split according to edge directionality inferred by Pearson correlation coefficient of normalized expression levels ($r > 0$ [+]: outer ring; $r < 0$ [-]: inner ring). Individual subplots display the subnetwork origin of the target while the color-coding depicts the subnetwork origin of the respective regulator.

S2d. Non-redundant intersections between the different gene sets. UpsetR plot displaying non-overlapping intersections between different gene sets discussed in the main text and figures (Fig. 2 and Supplementary Fig. S2b). The gene sets are available as individual lists in the supplementary file archive (`gene_sets.zip`).

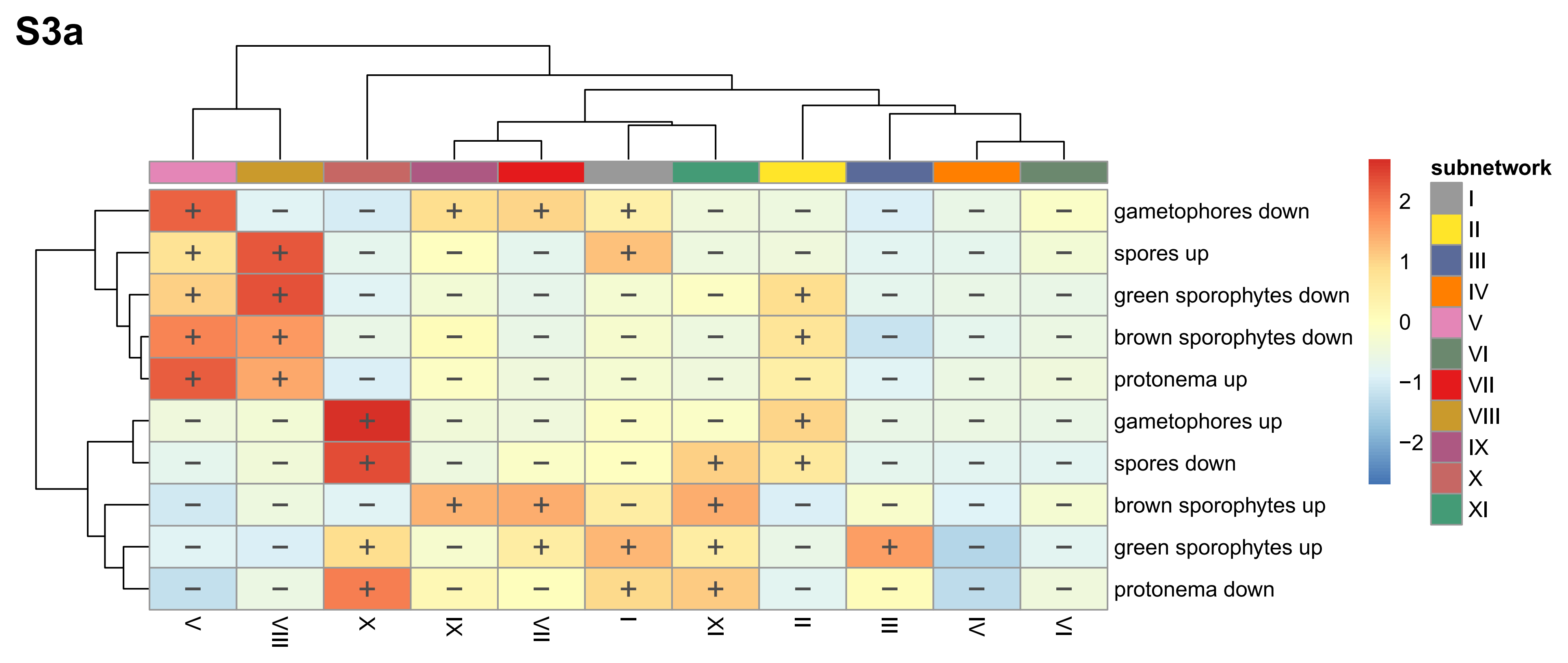
S3a
gametophores down
spores up
green sporophytes down
brown sporophytes down
protonema up
gametophores up
spores down
brown sporophytes up
green sporophytes up
protonema down
V
VIII
X
IX
VII
I
XI
II
III
IV
VI
2
1
0
−1
−2
subnetwork
I
II
III
IV
V
VI
VII
VIII
IX
X
XI

S3b

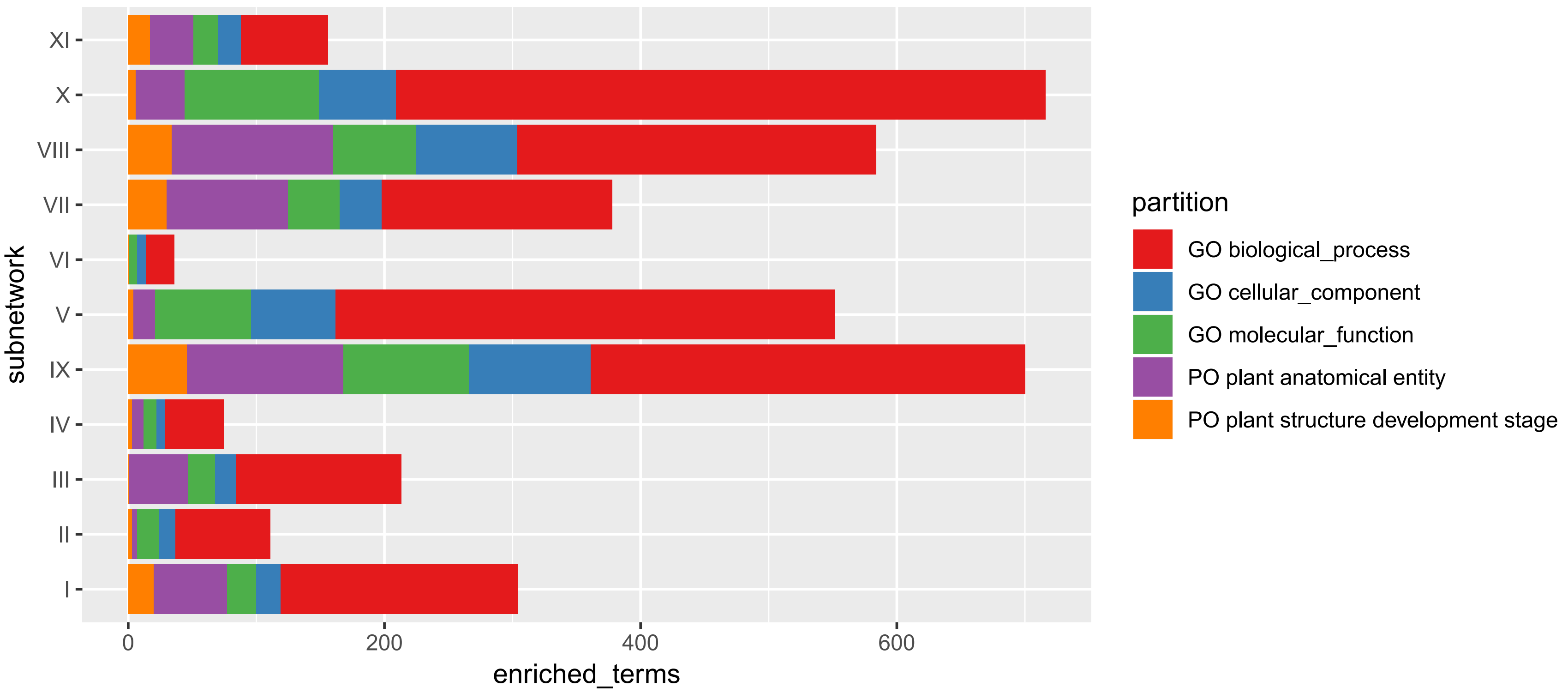

XI
X
VIII
VII
VI
V
IX
IV
III
II
I
subnetwork
0
200
400
600
enriched_terms
partition
GO biological_process
GO cellular_component
GO molecular_function
PO plant anatomical entity
PO plant structure development stage

S3c

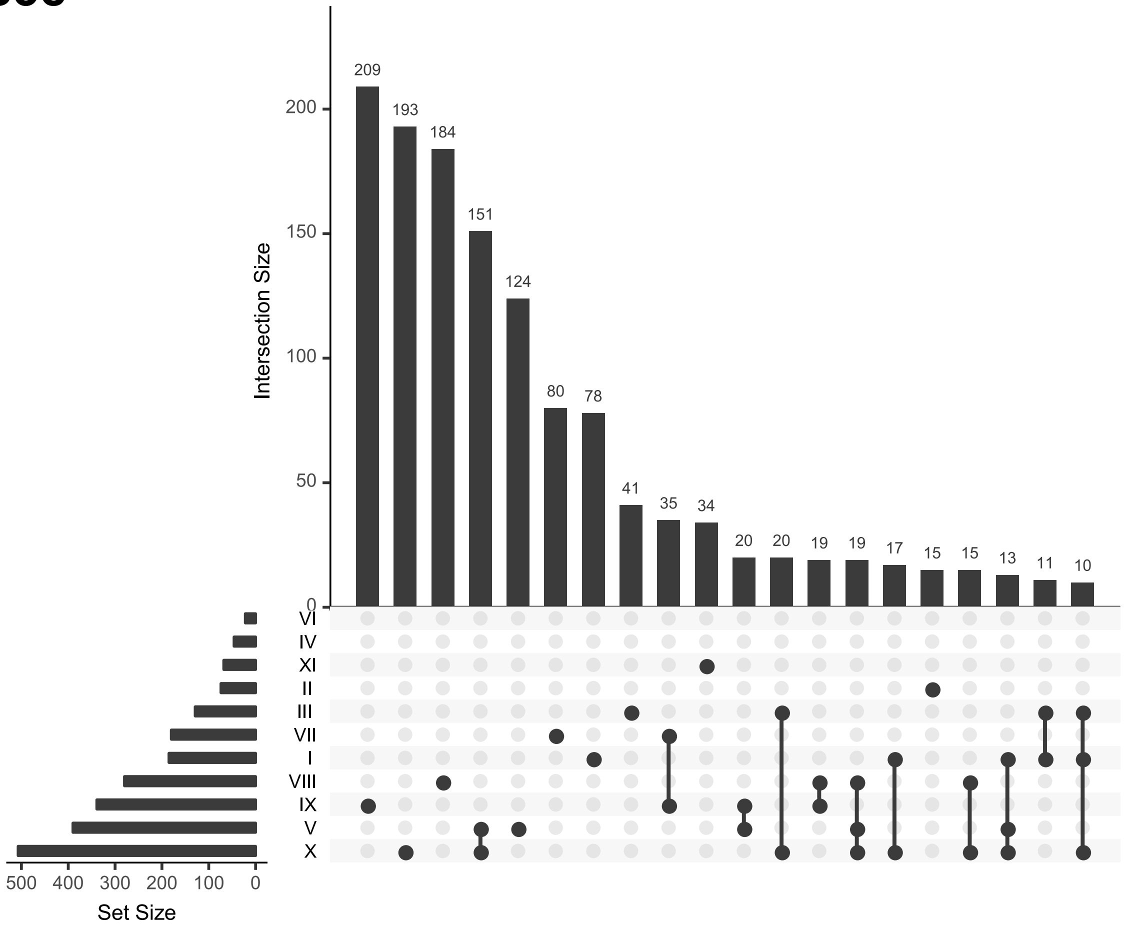

Intersection Size
200
150
100
50
0
209
193
184
151
124
80
78
41
35
34
20
20
19
19
17
15
15
13
11
10
VI
IV
XI
II
III
VII
I
VIII
IX
V
X
500 400 300 200 100 0
Set Size

S3d

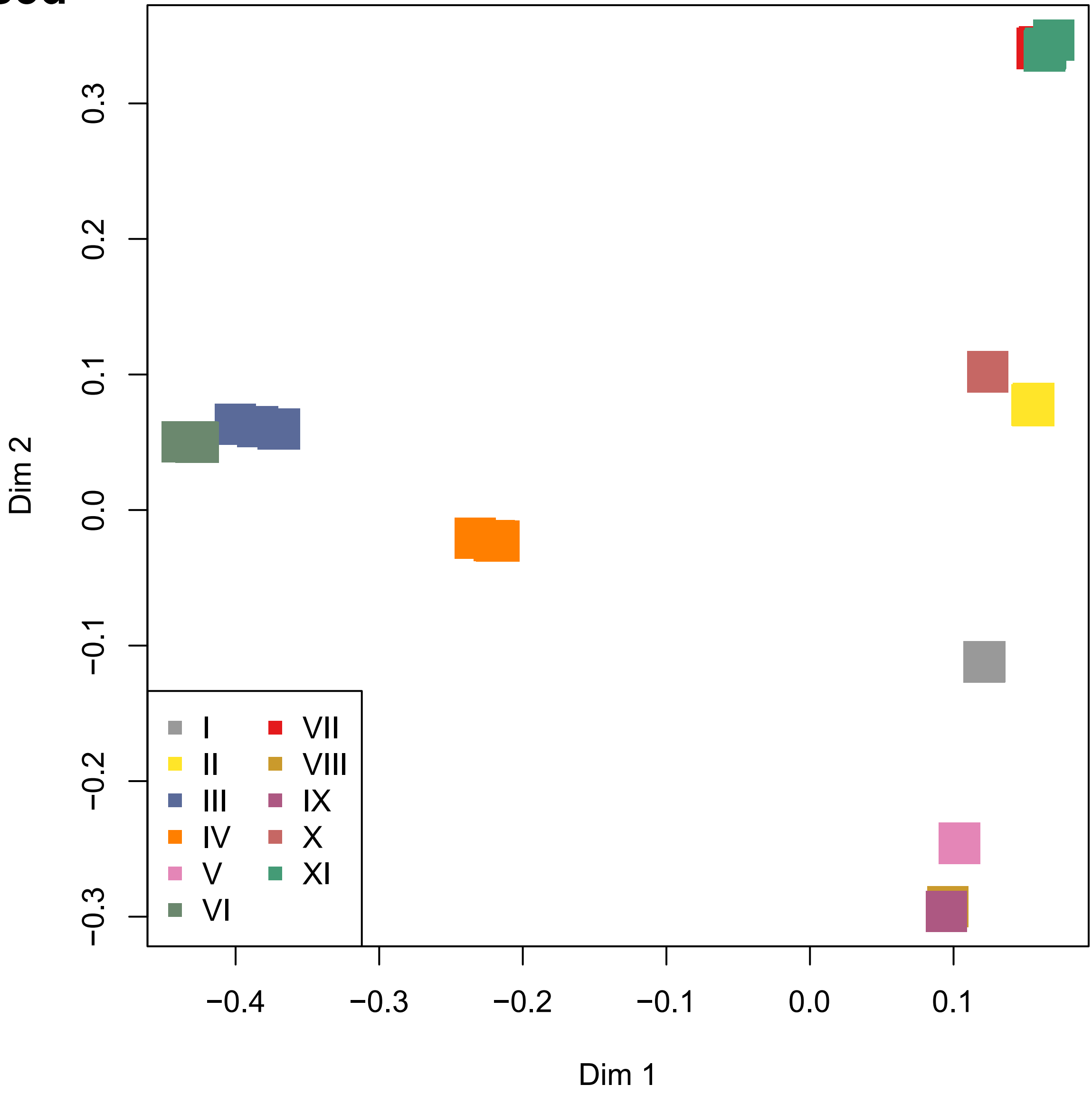

Dim 1
Dim 2
−0.4
−0.3
−0.2
−0.1
0.0
0.1
0.3
0.2
0.1
0.0
−0.1
−0.2
−0.3
I
II
III
IV
V
VI
VII
VIII
IX
X
XI

S3e

gametophyte vegetative stage
response to carbohydrate
cell periphery
organelle lumen
nucleus
cytosol
response to lipid
response to endogenous stimulus
protonema sub−apical initial cell
photoperiodism
external encapsulating structure
long−day photoperiodism
protonema whole plant development stage

S3f

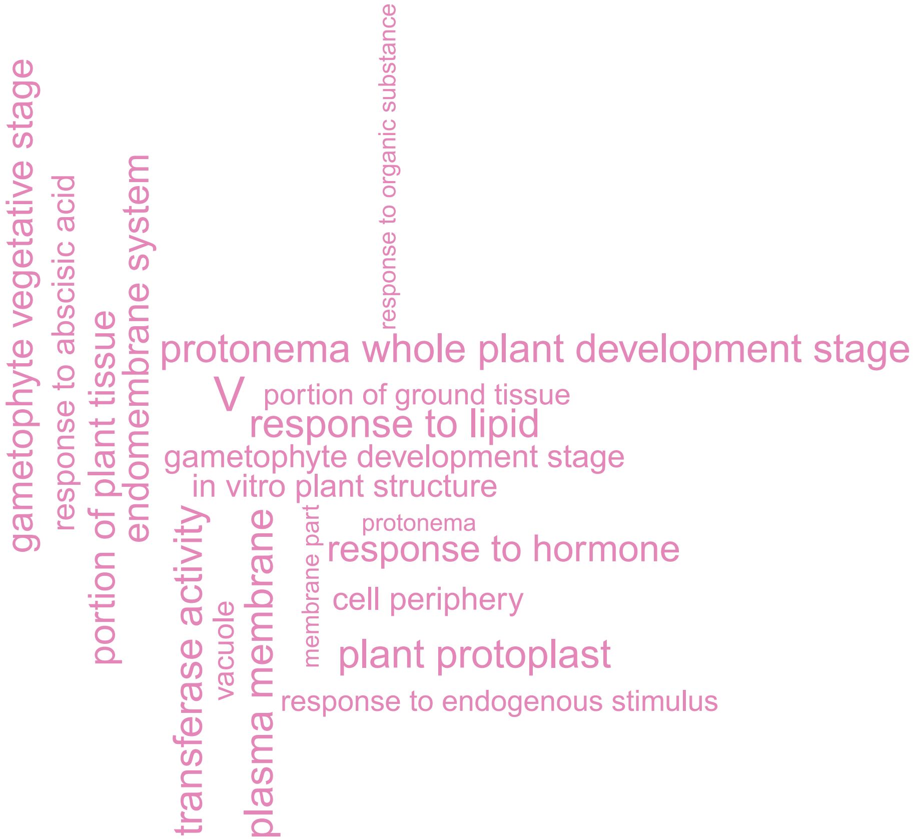

response to organic substance
gametophyte vegetative stage
response to abscisic acid
portion of plant tissue
endomembrane system
protonema whole plant development stage
V
portion of ground tissue
response to lipid
gametophyte development stage
in vitro plant structure
transferase activity
vacuole
plasma membrane
membrane part
protonema
response to hormone
cell periphery
plant protoplast
response to endogenous stimulus

S3g

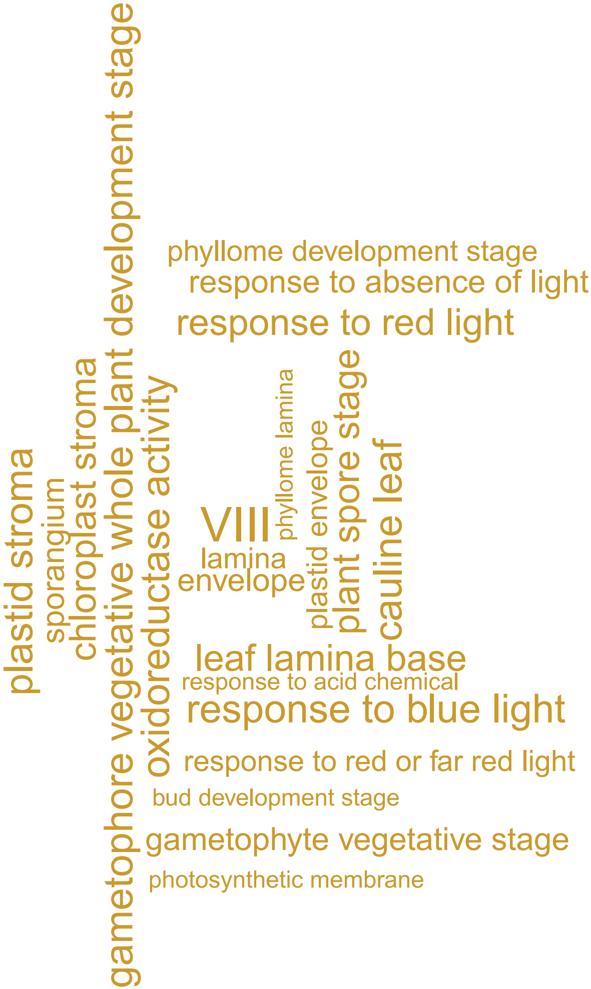
gametophore vegetative whole plant development stage
phyllome development stage
response to absence of light
response to red light
plastid stroma
sporangium
chloroplast stroma
oxidoreductase activity
VIII
lamina
envelope
phyllome lamina
plastid envelope
plant spore stage
cauline leaf
leaf lamina base
response to acid chemical
response to blue light
response to red or far red light
bud development stage
gametophyte vegetative stage
photosynthetic membrane

S3h

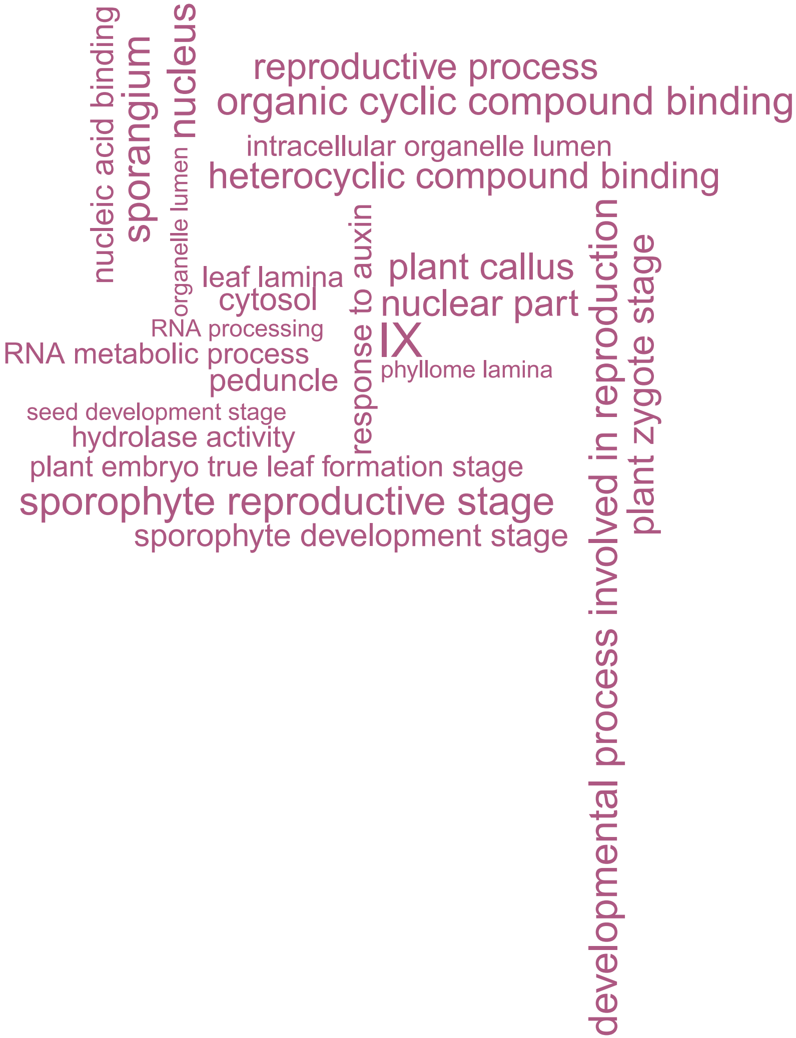
nucleic acid binding
sporangium
nucleus
organelle lumen
reproductive process
organic cyclic compound binding
intracellular organelle lumen
heterocyclic compound binding
response to auxin
plant callus
leaf lamina
cytosol
nuclear part
RNA processing
IX
RNA metabolic process
phyllome lamina
peduncle
seed development stage
hydrolase activity
plant embryo true leaf formation stage
sporophyte reproductive stage
sporophyte development stage
developmental process involved in reproduction
plant zygote stage

S3i

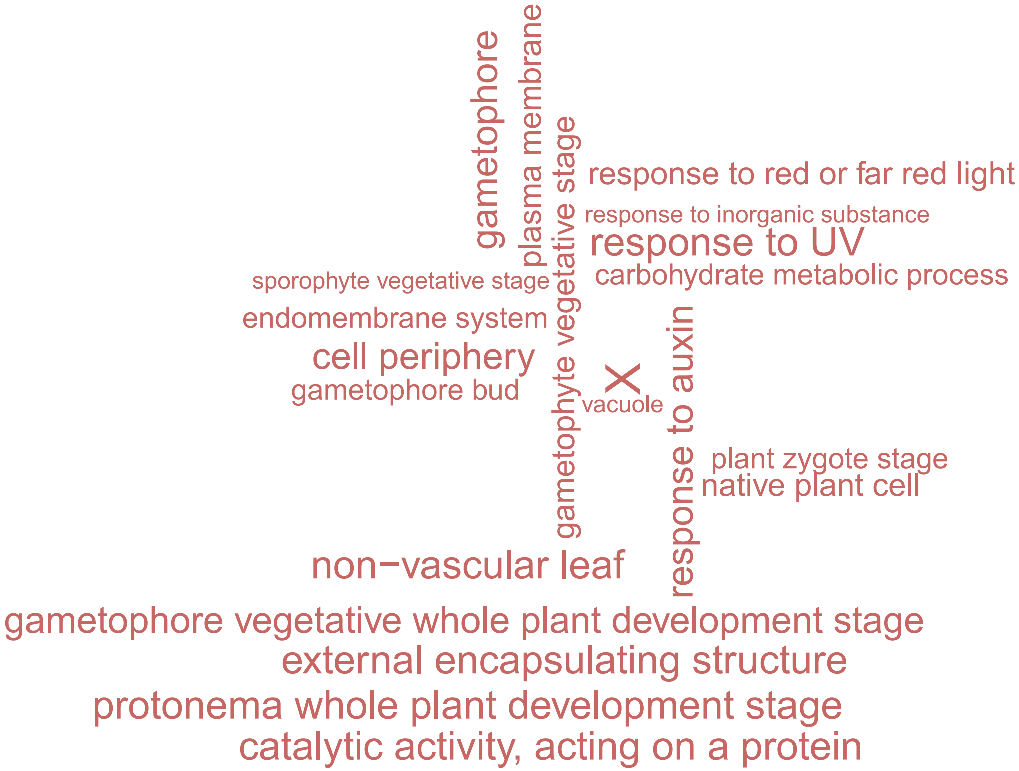
gametophore
plasma membrane
gametophyte vegetative stage
response to red or far red light
response to inorganic substance
response to UV
sporophyte vegetative stage
carbohydrate metabolic process
endomembrane system
cell periphery
gametophore bud
X
vacuole
response to auxin
plant zygote stage
native plant cell
non−vascular leaf
gametophore vegetative whole plant development stage
external encapsulating structure
protonema whole plant development stage
catalytic activity, acting on a protein

Supplementary Fig. S3.

Functional characterization of the subnetworks. We utilized the developmental stage samples included in the *P. patens* Gene Atlas[1] as well as Plant Ontology and Gene Ontology annotations for functional characterization of the subnetworks generated in this study. Both approaches independently considered the network's structure to assess overrepresentation of functional concepts among the genes in the network. The ontology analysis comprised a multi-step procedure relying on a machine learning approach to identify the most specific and characteristic terms for the genes encoded in each subnetwork. The final set of most characteristic PO and GO terms for each enriched subnetwork (Panels S3e-i) comprises the ontology terms that were most informative to classify the top20 master regulators from each subnetwork according to their targets' functional composition.

S3a. Network enrichment analysis of differentially expressed genes in developmental stages from the *P. patens* Expression Atlas. We inferred up- and down-regulated genes for each developmental stage represented in the Gene Atlas data by performing differential gene expression analysis using a combination of the likelihood ratio test (LRT) and Wald test implemented in R/sleuth[2] comparing the samples derived from the respective stage with all other samples. Results were filtered using LRT- qvalue <0.1 and classified into up and down-regulation based on b-values of the Wald test. Individual gene sets were tested for undirected enrichment in the subnetworks using the R/NEAT software package. Results were plotted as ratios of observed/expected values in a heatmap using the R/pheat package annotating significant (FDR < 0.01) enrichment and depletion.

S3b. Subnetwork ontology enrichment analysis. We performed ontology enrichment analysis for the subnetworks based on an updated Plant Ontology (PO) and Gene Ontology (GO) annotations available as part of the supplementary file archive using the command line version of Ontologizer with Benjamini-Hochberg (FDR) correction at 90% confidence. The stacked barchart depicts the absolute numbers of filtered enriched terms.

S3c. Non-redundant overlap among GO biological process (BP) concepts among the subnetworks. Consistent with gene regulatory subnetworks potentially encoding distinct cell fates, enriched terms overlapped among some of the subnetworks. The bar chart displays an UpsetR analysis of the 20 largest non-redundant sets of enriched GO BP terms.

S3d. Functional similarity of the subnetworks' top20 master regulators' targets. In order to identify the most specific enriched terms considering the network structure and hierarchy, we employed the Random Forest machine learning approach classifying subnetwork membership of the master regulators using their targets' association among the globally enriched GO and PO terms. Globally enriched terms were selected analyzing enrichment in the subnetworks regulatory links using R/NEAT with FDR <0.01. Multidimensional scaling plot of proximity matrix from R/randomForest of the top20 master regulators for each subnetwork.

S3e-i. Top5 most specific concepts to describe targets in subnetworks II, V, VIII, IX and X. Word cloud representations of the most important terms for each subnetwork to classify subnetwork membership of the top20 master regulators using the Random Forest predictor. We selected and ranked terms for each subnetwork demanding variable importance >0 using the decrease in node impurity based on the Gini index implemented by the R/randomForest package. See Supplementary Data sheet S3 for complete results.

S4

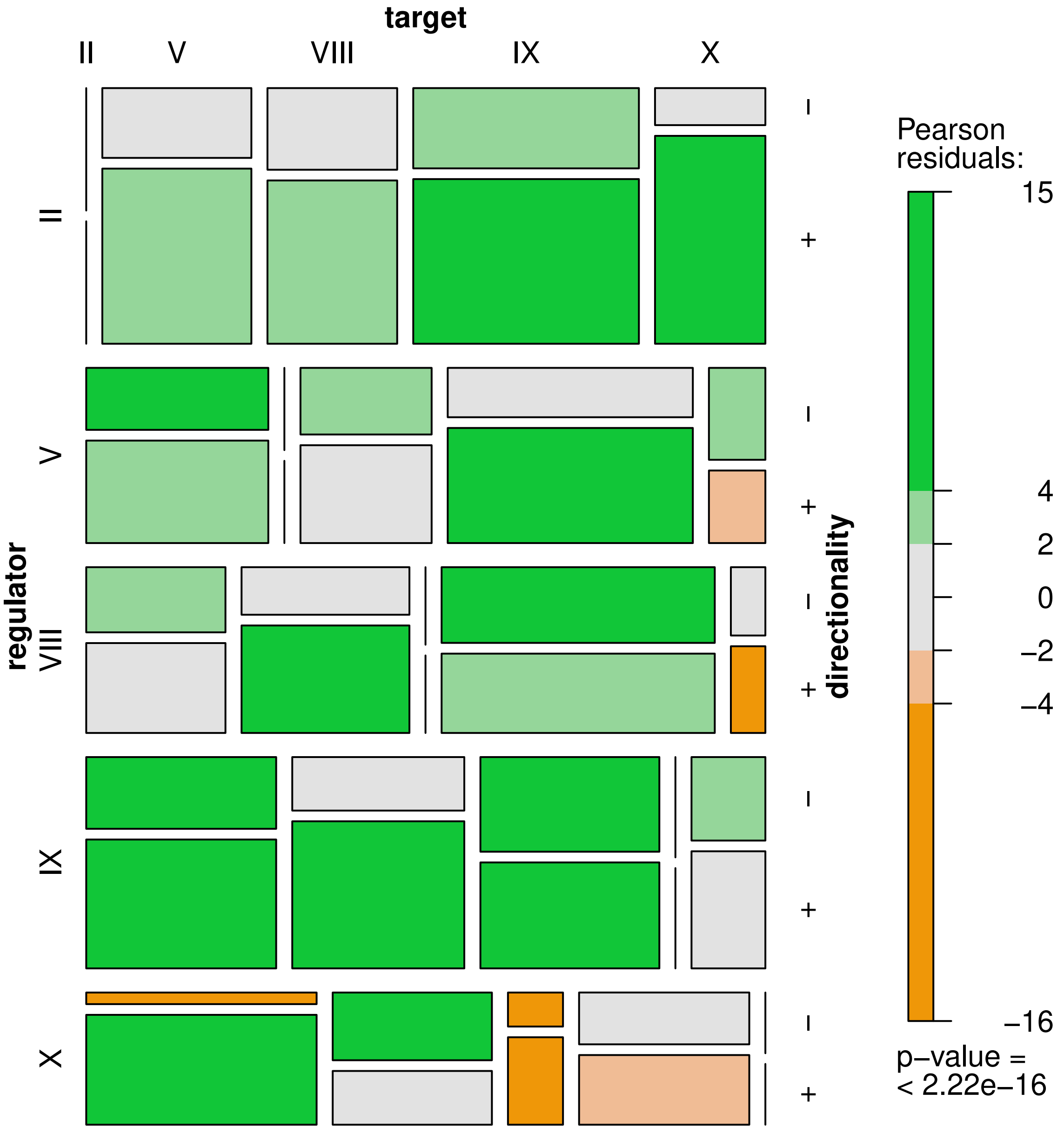
target
II
V
VIII
IX
X
regulator
II
V
VIII
IX
X
directionality
-
+
Pearson
residuals:
15
4
2
0
−2
−4
−16
p−value =
< 2.22e−16

Supplementary Fig. S4.

Mosaic plot of inter-subnetwork connections. Mosaic plot or *Marimekko diagram* showing the cross-sectional distribution of inter-subnetwork connections considering the predicted directionality based on the Pearson correlation coefficient of expression profiles between a regulator and a predicted target from another subnetwork. Color-scale of the boxes depicts the distribution of Pearson residuals indicating significant over- (green) or under- (orange) representation of inter-connecting edges obtained from a significant X^2 test (p-value < $2.22e^{-16}$).

S5a

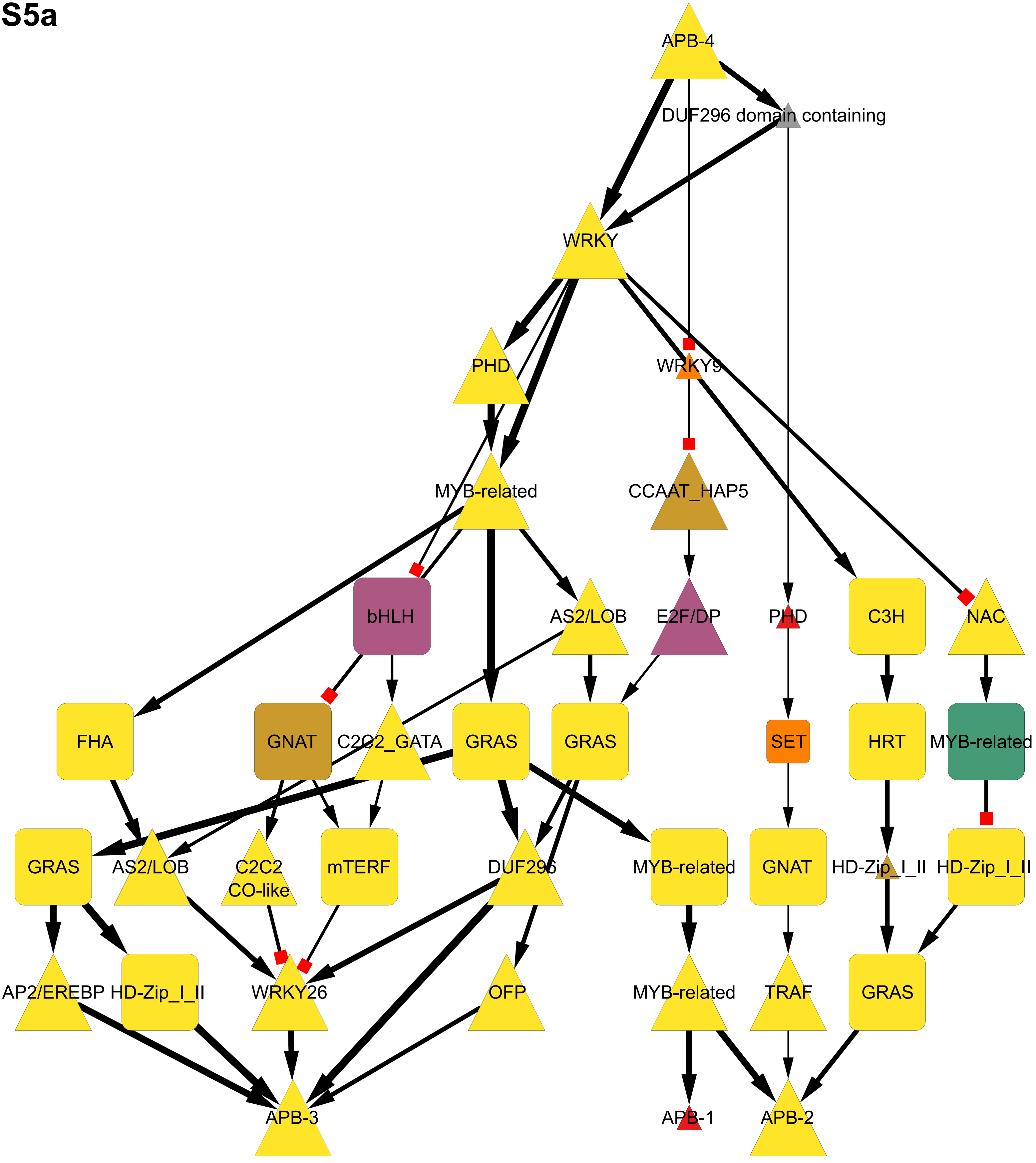
APB-4
DUF296 domain containing
WRKY
PHD
WRKY9
MYB-related
CCAAT_HAP5
bHLH
AS2/LOB
E2F/DP
PHD
C3H
NAC
FHA
GNAT
C2C2_GATA
GRAS
GRAS
SET
HRT
MYB-related
GRAS
AS2/LOB
C2C2
CO-like
mTERF
DUF296
MYB-related
GNAT
HD-Zip_I_II
HD-Zip_I_II
AP2/EREBP
HD-Zip_I_II
WRKY26
OFP
MYB-related
TRAF
GRAS
APB-3
APB-1
APB-2

S5b

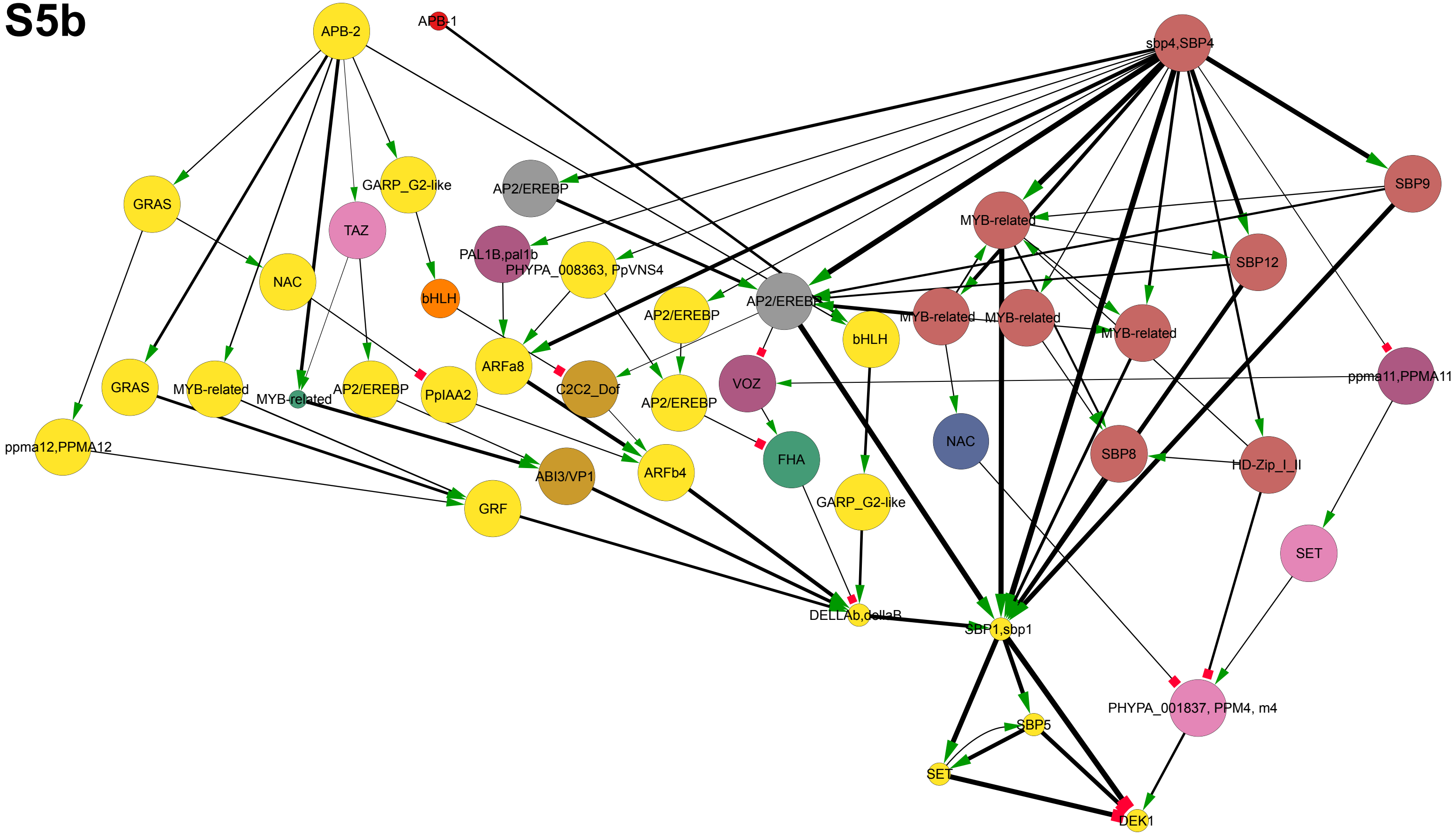
APB-2
APB-1
sbp4,SBP4
GRAS
GARP_G2-like
AP2/EREBP
TAZ
SBP9
MYB-related
PAL1B,pal1b
PHYPA_008363, PpVNS4
SBP12
NAC
bHLH
AP2/EREBP
AP2/EREBP
MYB-related
MYB-related
MYB-related
bHLH
ARFa8
C2C2_Dof
VOZ
ppma11,PPMA11
GRAS
MYB-related
MYB-related
AP2/EREBP
PpIAA2
AP2/EREBP
NAC
ppma12,PPMA12
ABI3/VP1
ARFb4
FHA
SBP8
HD-Zip_I_II
GRF
GARP_G2-like
SET
DELLAb,dellaB
SBP1,sbp1
PHYPA_001837, PPM4, m4
SBP5
SET
DEK1

Supplementary Fig. S5.
K-shortest simple path network graphs.

S5a. APB regulatory hierarchy. Network plot depicting the regulatory hierarchy of the AINTEGUMENTA, PLETHORA and BABY BOOM (APB) subfamily[28, 29] of AP2/ANT transcription factors in *P. patens*[5]. The network subgraph was extracted using the PathLinker[6] Cytoscape app (k = 20) and visualized in Cytoscape[7]. Nodes are color-coded by subnetwork identity. Node shapes differentiate between NERD-targeted calpain cleavages (Arg-N-end rule = triangle) and no or other cleavages (round rectangle). Edge arrows indicate predicted directionality based on the Pearson correlation coefficient of expression profiles (R >0 black arrowhead; R <0 red square). Edge width is based on the path rank score obtained from the PathLinker algorithm (1. rank = thickest).

S5b. DEK1 upstream regulon. Network plot depicting the upstream regulatory context of DEK1. The network subgraph was extracted using the PathLinker Cytoscape app (k = 50) and visualized in Cytoscape. Nodes are color-coded by subnetwork identity. Node shapes differentiate between NERD-targeted calpain cleavages (Arg-N-end rule = triangle) and no or other cleavages (round rectangle). Edge arrows indicate predicted directionality based on the Pearson correlation coefficient of expression profiles (R >0 black arrowhead; R <0 red square). Edge width is based on the path rank score obtained from the PathLinker algorithm (1. rank = thickest).

S6a

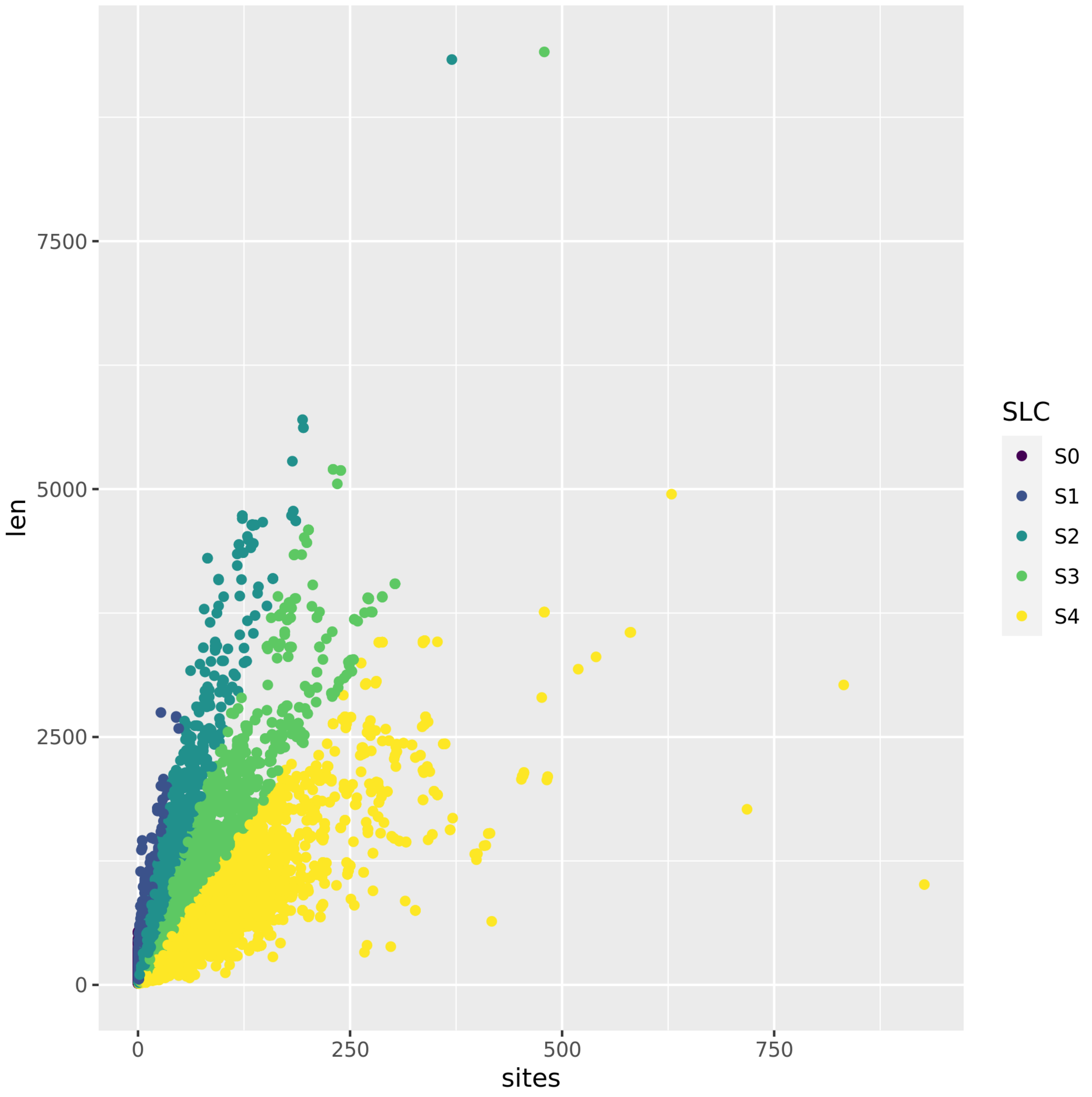

S6b

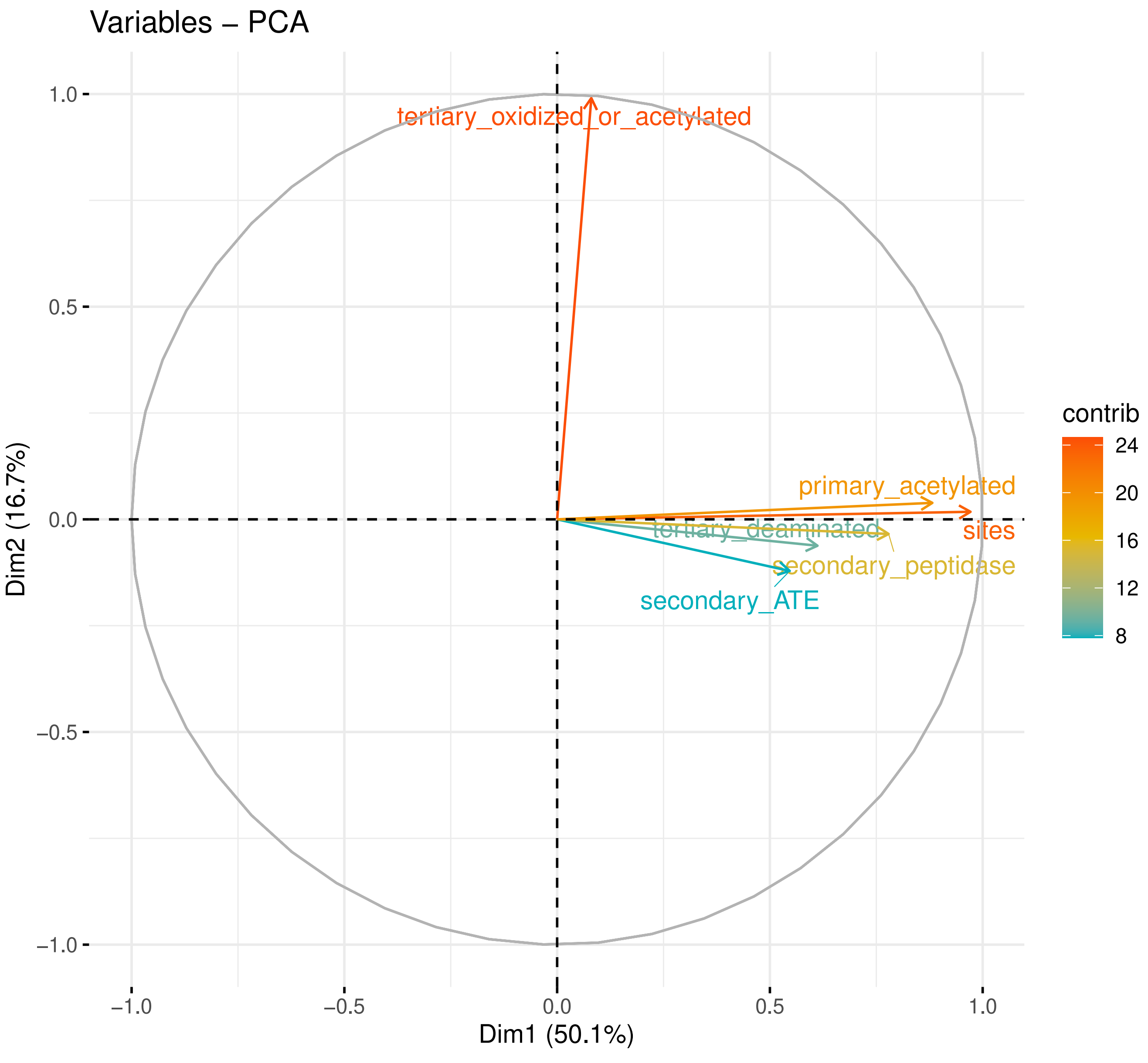

Variables – PCA
tertiary_oxidized_or_acetylated
primary_acetylated
tertiary_deaminated
sites
secondary_peptidase
secondary_ATE
Dim1 (50.1%)
Dim2 (16.7%)
−1.0
−0.5
0.0
0.5
1.0
contrib
24
20
16
12
8

S6c

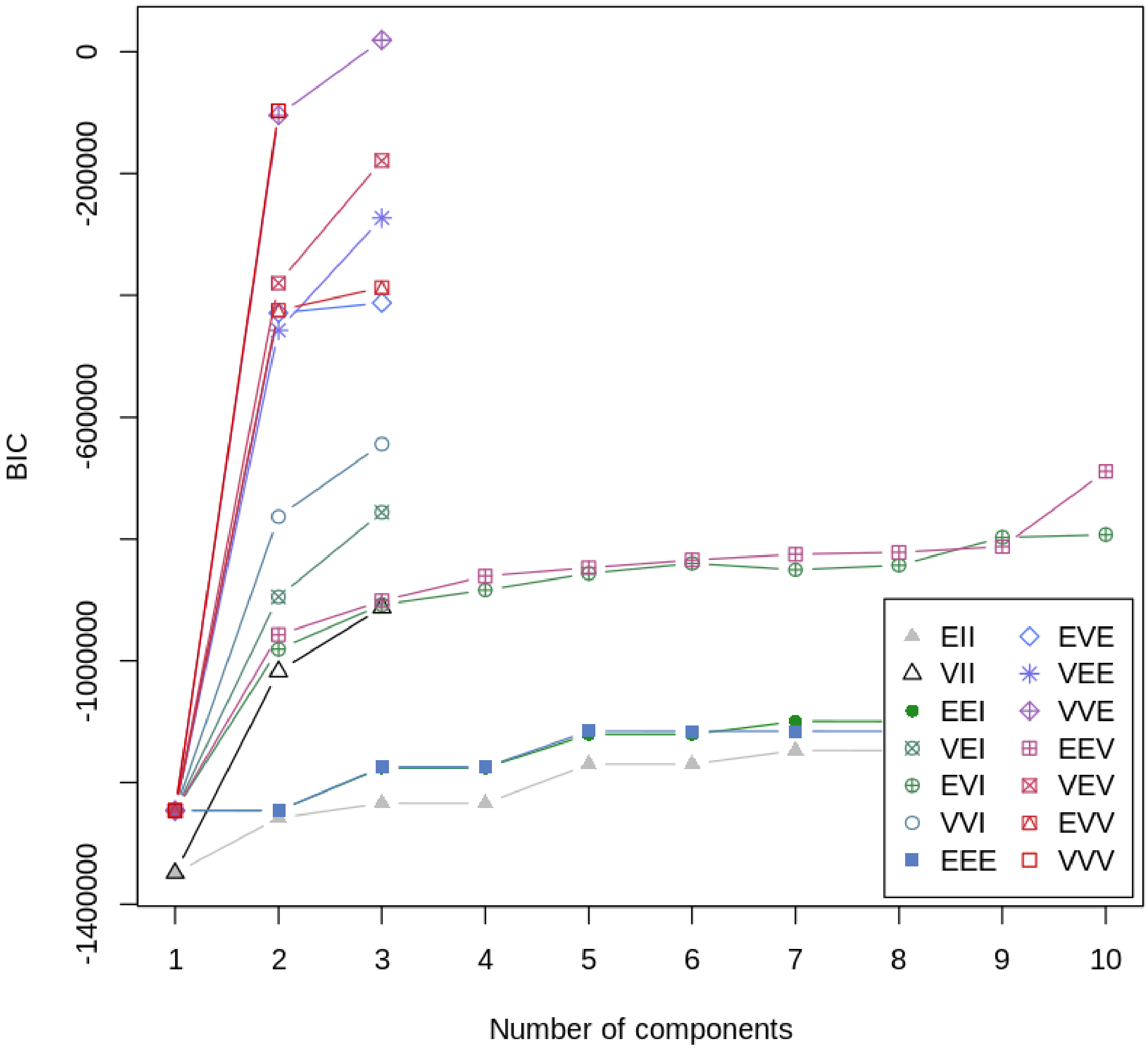

BIC
0
-200000
-600000
-1000000
-1400000
1
2
3
4
5
6
7
8
9
10
Number of components
EII
VII
EEI
VEI
EVI
VVI
EEE
EVE
VEE
VVE
EEV
VEV
EVV
VVV

S6d

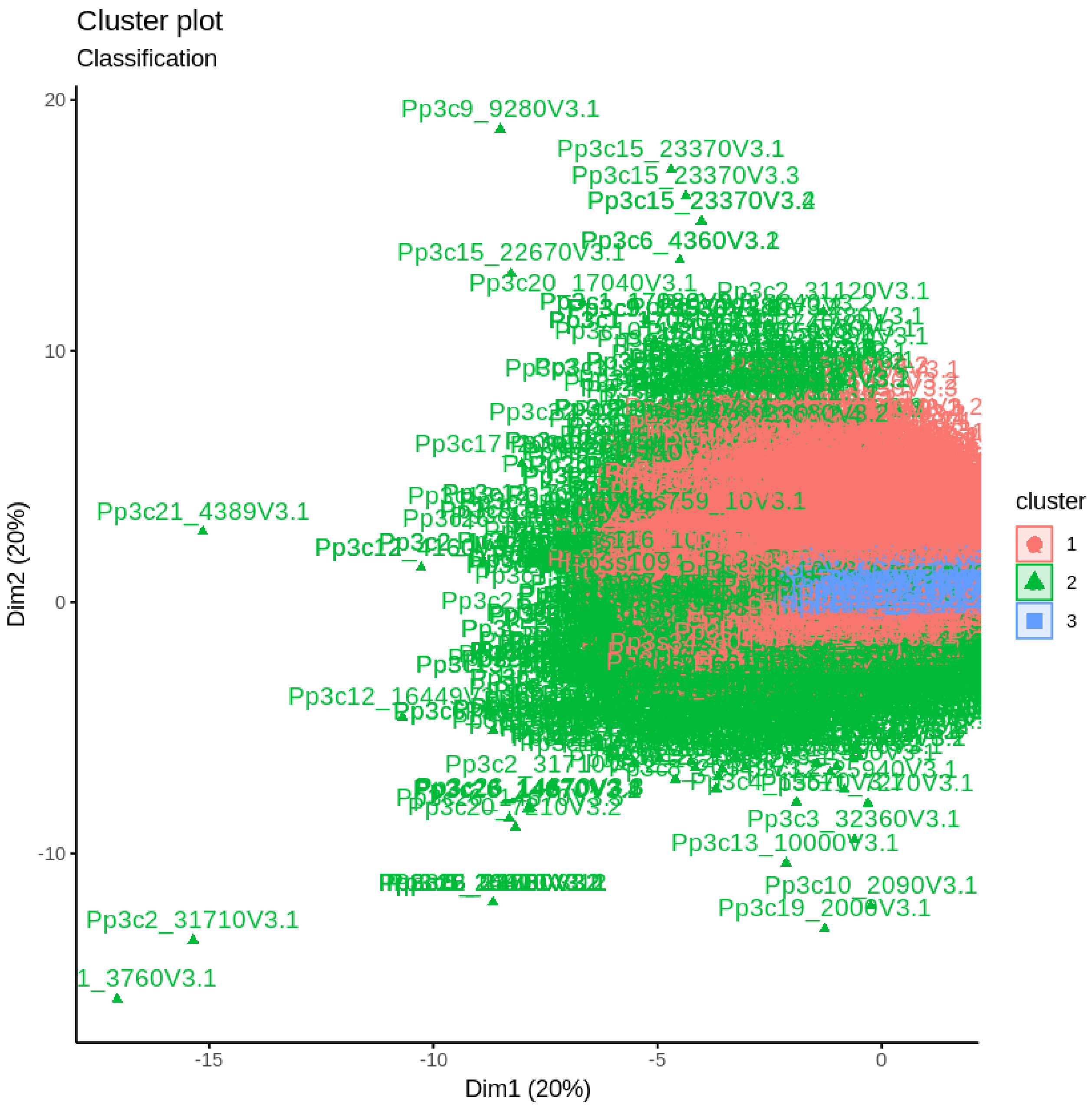
Cluster plot
Classification
Dim2 (20%)
Dim1 (20%)
20
10
0
-10
-15
-10
-5
0
cluster
1
2
3
Pp3c9_9280V3.1
Pp3c15_23370V3.1
Pp3c15_23370V3.3
Pp3c15_22670V3.1
Pp3c20_17040V3.1
Pp3c2_31120V3.1
Pp3c21_4389V3.1
Pp3c2_31710V3.1
Pp3c3_32360V3.1
Pp3c10_2090V3.1
1_3760V3.1

S6e

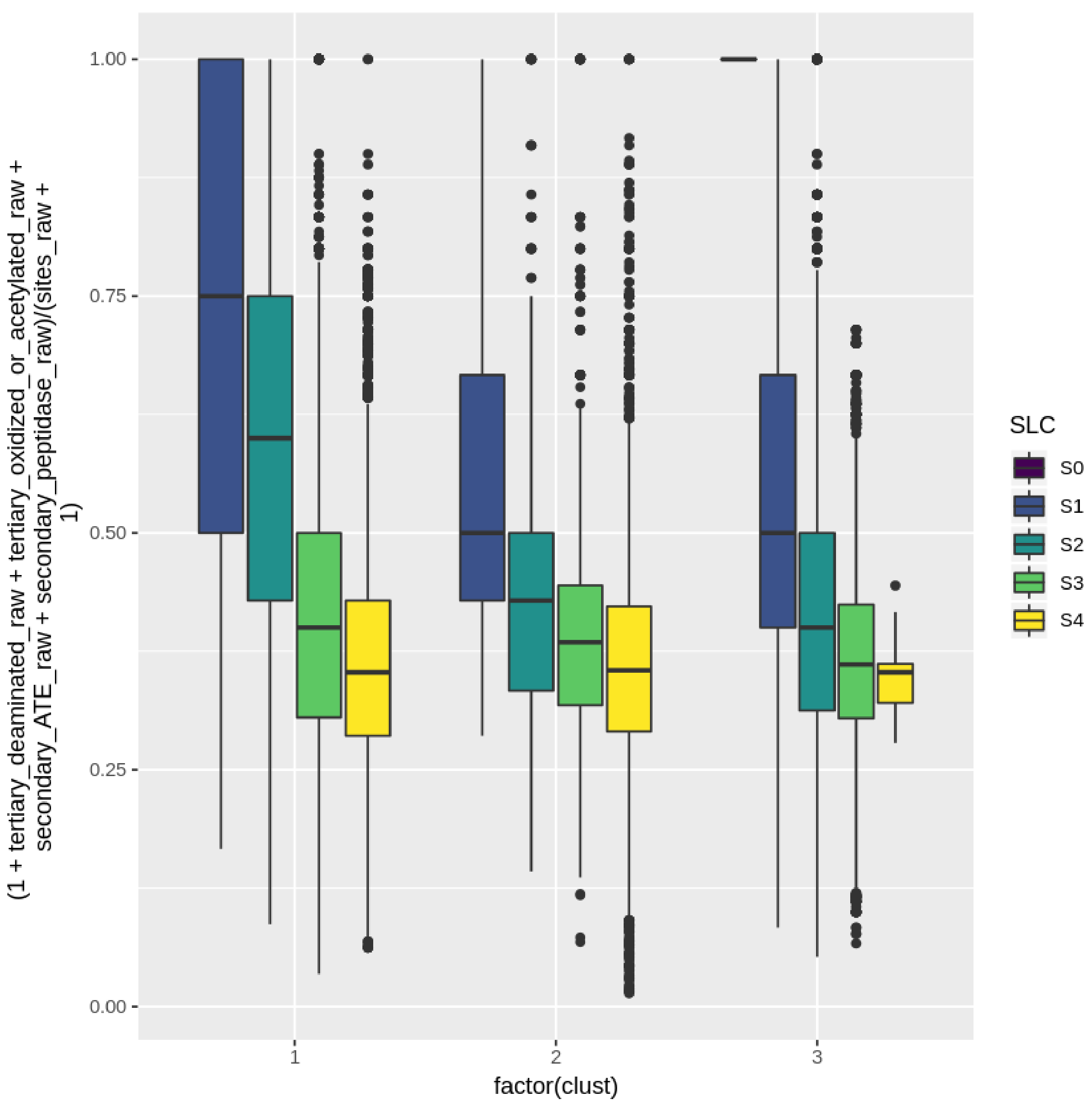

(1 + tertiary_deaminated_raw + tertiary_oxidized_or_acetylated_raw + secondary_ATE_raw + secondary_peptidase_raw)/(sites_raw + 1)
1.00
0.75
0.50
0.25
0.00
1
2
3
factor(clust)
SLC
S0
S1
S2
S3
S4

S6f

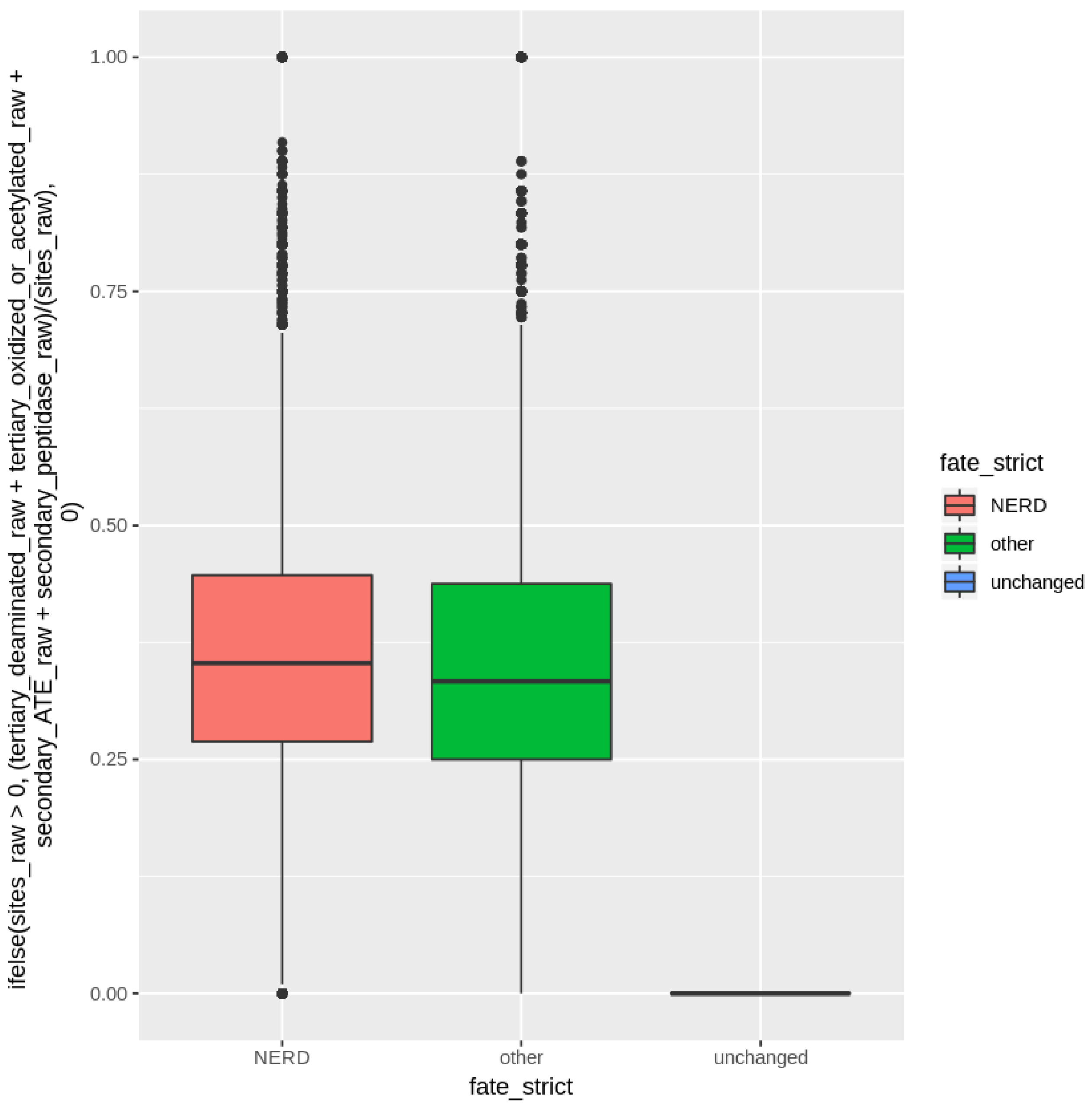
ifelse(sites_raw > 0, (tertiary_deaminated_raw + tertiary_oxidized_or_acetylated_raw + secondary_ATE_raw + secondary_peptidase_raw)/(sites_raw), 0)
1.00
0.75
0.50
0.25
0.00
NERD
other
unchanged
fate_strict
fate_strict
NERD
other
unchanged

S6g

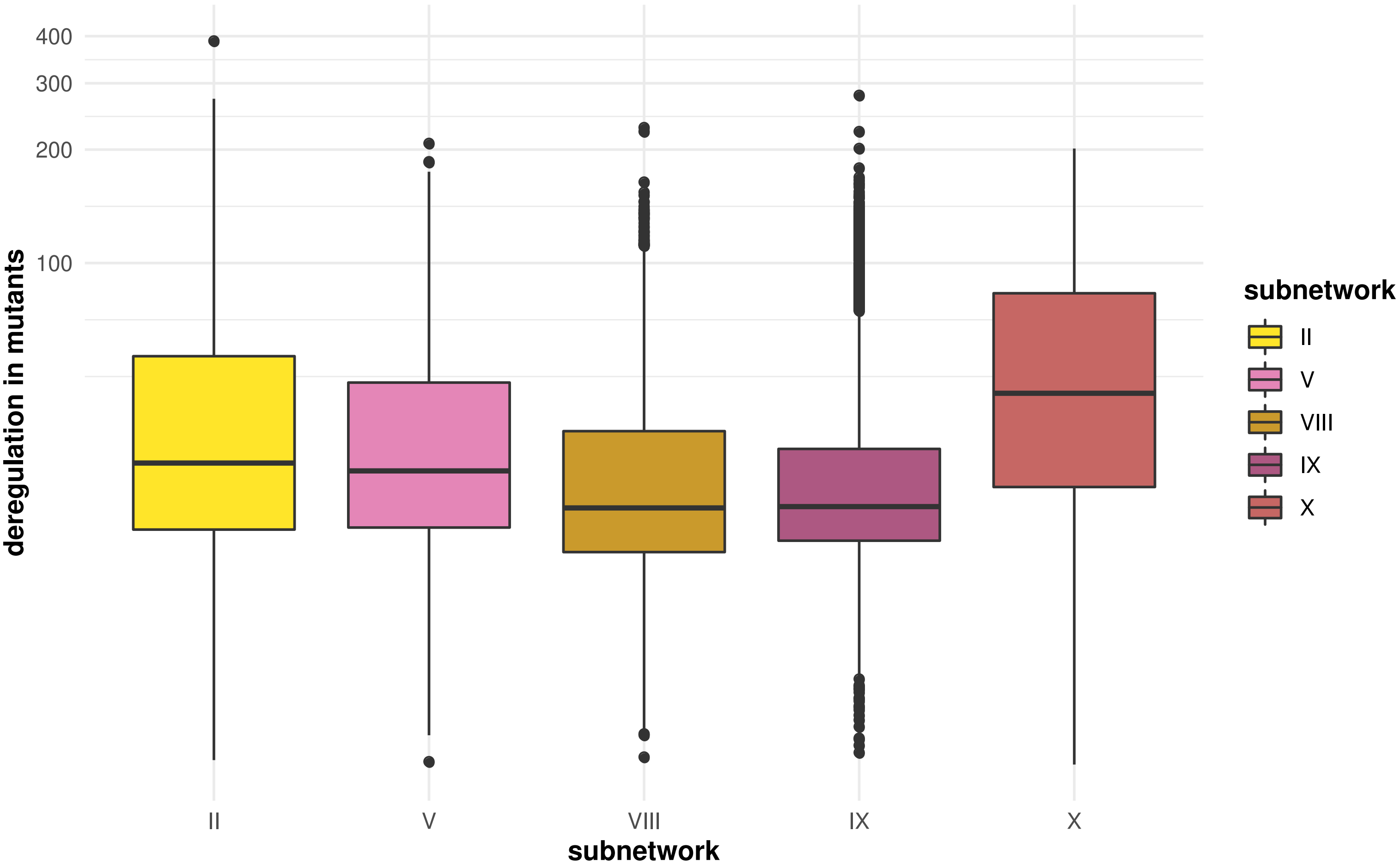
400
300
200
100
deregulation in mutants
II
V
VIII
IX
X
subnetwork
subnetwork
II
V
VIII
IX
X

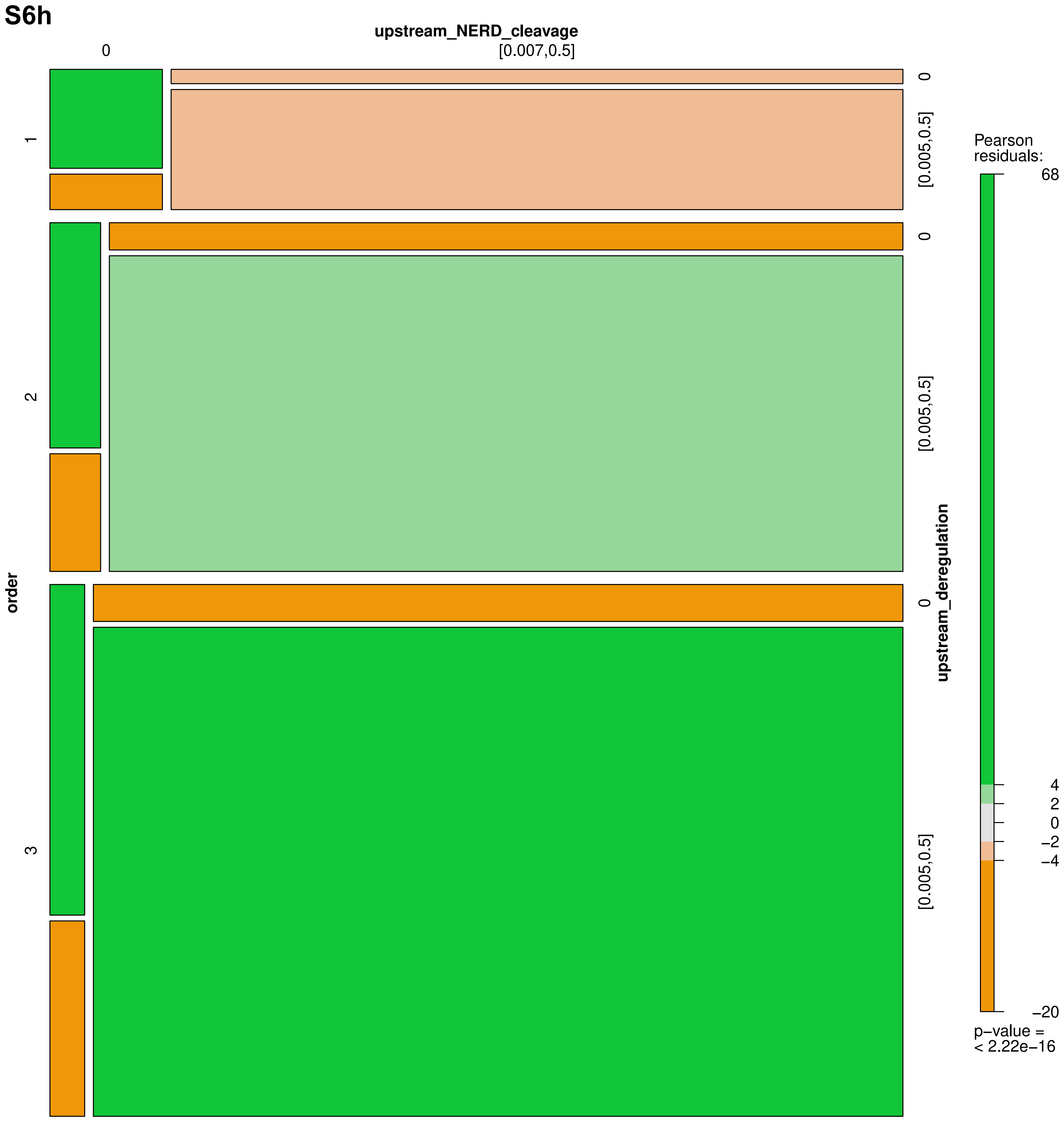
S6h
upstream_NERD_cleavage
0
[0.007,0.5]
order
1
2
3
upstream_deregulation
0
[0.005,0.5]
Pearson residuals:
68
4
2
0
−2
−4
−20
p−value =
< 2.22e−16

Supplementary Fig. S6.
Prediction of direct and indirect DEK1 targets.

S6a. Scatter plot of protein length vs. number of predicted calpain cleavage sites. Overall site frequencies were scaled by the total protein length. Log-transformed, scaled overall site frequencies were clustered with k-means clustering into five site abundance level categories (SLC; point colors), representing: S0 = no; S1 = very few – few; S2 = few – medium; S3 = medium – many; S4 = many - very many predicted sites. These labels were obtained by ranking the resulting k-means clusters by their centroid/mean number of predicted cleavage sites.

S6b. PCA of calpain cleavage site predictions. Overall site frequency and individual NERD site type frequencies (see Fig. 3a and Methods) were scaled by the total protein length and analyzed by PCA. Eigenvector plot of the first two principal components. While overall site frequency and primary acetylated sites contribute only to the first dimension, the second dimension separates the NERD-like signatures.

S6c. Model-based clustering of calpain cleavage site predictions. Line plot depicting the Bayesian Information Criterion vs. the number of resulting clusters (components) using different clustering models. The VVE model resulted in the optimal BIC.

S6d. Optimal model-based clustering (VVE model) of calpain cleavage site predictions. Cluster scatter plot of the first two principal components of a PCA of calpain cleavage site classifications for *P. patens* proteins.

S6e. Fractions of NERD-like cleavages among the VVE clusters color-coded by the overall abundance level of predicted sites. Box-whisker plots depicting the distribution of the relative proportion of NERD-like cleavages. Proteins with no predicted cleavages were represented as 1. Color-coding according to five site abundance level categories (SLC; point colors), representing: S0 = no; S1 = very few – few; S2 = few – medium; S3 = medium – many; S4 = many - very many predicted sites. X-axis corresponds to the three identified clusters obtained by model-based clustering via VVE.

S6f. Fractions of NERD-like cleavages among the VVE clusters. Box-whisker plots depicting the distribution of the relative proportion of NERD-like cleavages. Proteins with no predicted cleavages were represented as 0. Color-coding based on VV3 clustering.

S6g. Absolute deregulation of subnetworks in *DEK1* mutants. Box-whisker plots depicting the distribution of the cumulative level of deregulation of genes with significantly altered expression levels in the mutants encoded by the five DEK1-controlled subnetworks. The levels of deregulation were represented by the sum of the $\chi 2$ test statistic of the likelihood ratio tests (LRTs) comparing wild type vs. *Δdek1*, *oex1* vs. wild type and *Δdek1* vs. *oex1* employing it in the sense of an absolute, cumulative effect size. Fill-color depicts subnetwork affiliation.

S6h. Regulatory cascades demonstrate consistent deregulation patterns. Mosaic plot or *Marimekko diagram* depicting the three-way cross-tabulation of three three factorial variables: left axis – order of the regulon i.e. 1st order: TF→target; 2nd: TF→TF→target and 3rd: TF→TF→TF→target. Upper and left axis represent binary factors representing the binary status of their upstream regulon with respect to predicted levels of DEK1 control.

Upper axis – upstream_NERD_cleavage – i.e. predicted direct DEK1 cleavage targets. Right axis - indirect DEK1 targets i.e. deregulation of upstream TFs. Binary status defines whether the regulon comprises TFs predicted direct (x) or indirect (y) DEK1 targets (> 0% of the TFs) or not (= 0% TFs). Boxes are colored based on Pearson residuals from a significant $\chi 2$ test of the cross-table comparing the proportions of the three classes.

S7a

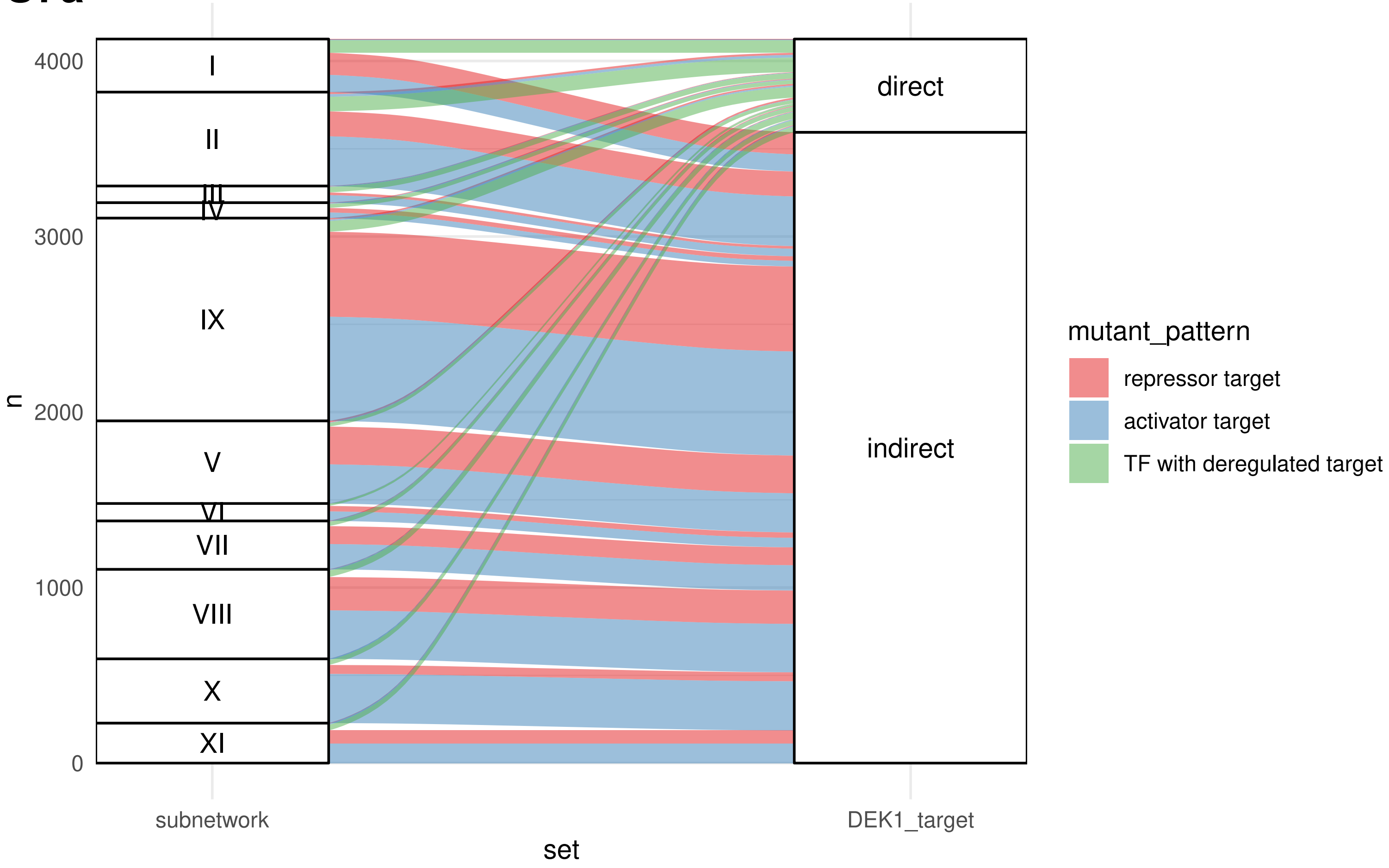

4000
3000
2000
1000
0
n
I
II
III
IV
IX
V
VI
VII
VIII
X
XI
direct
indirect
subnetwork
DEK1_target
set
mutant_pattern
repressor target
activator target
TF with deregulated target

S7b

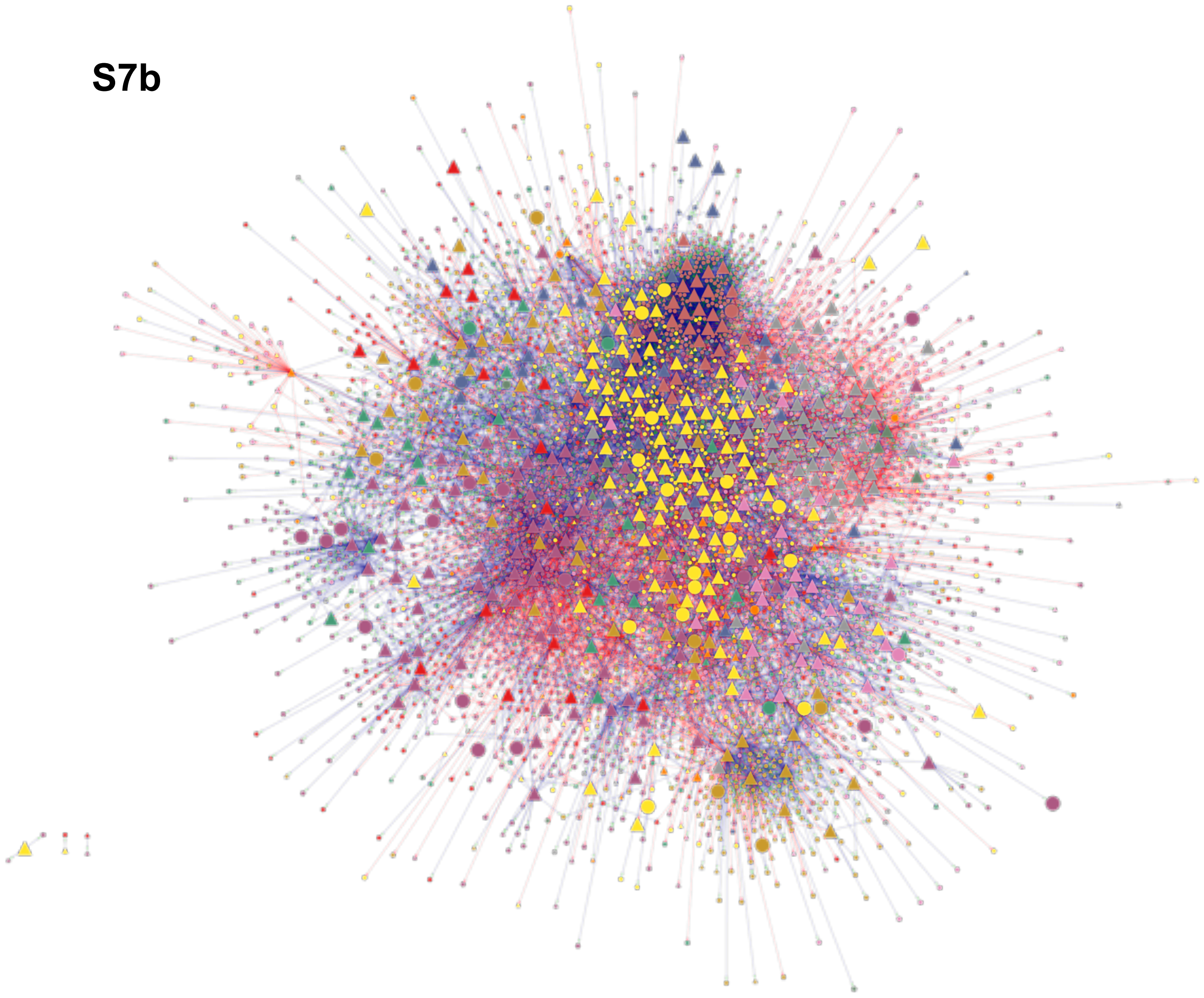

S7c

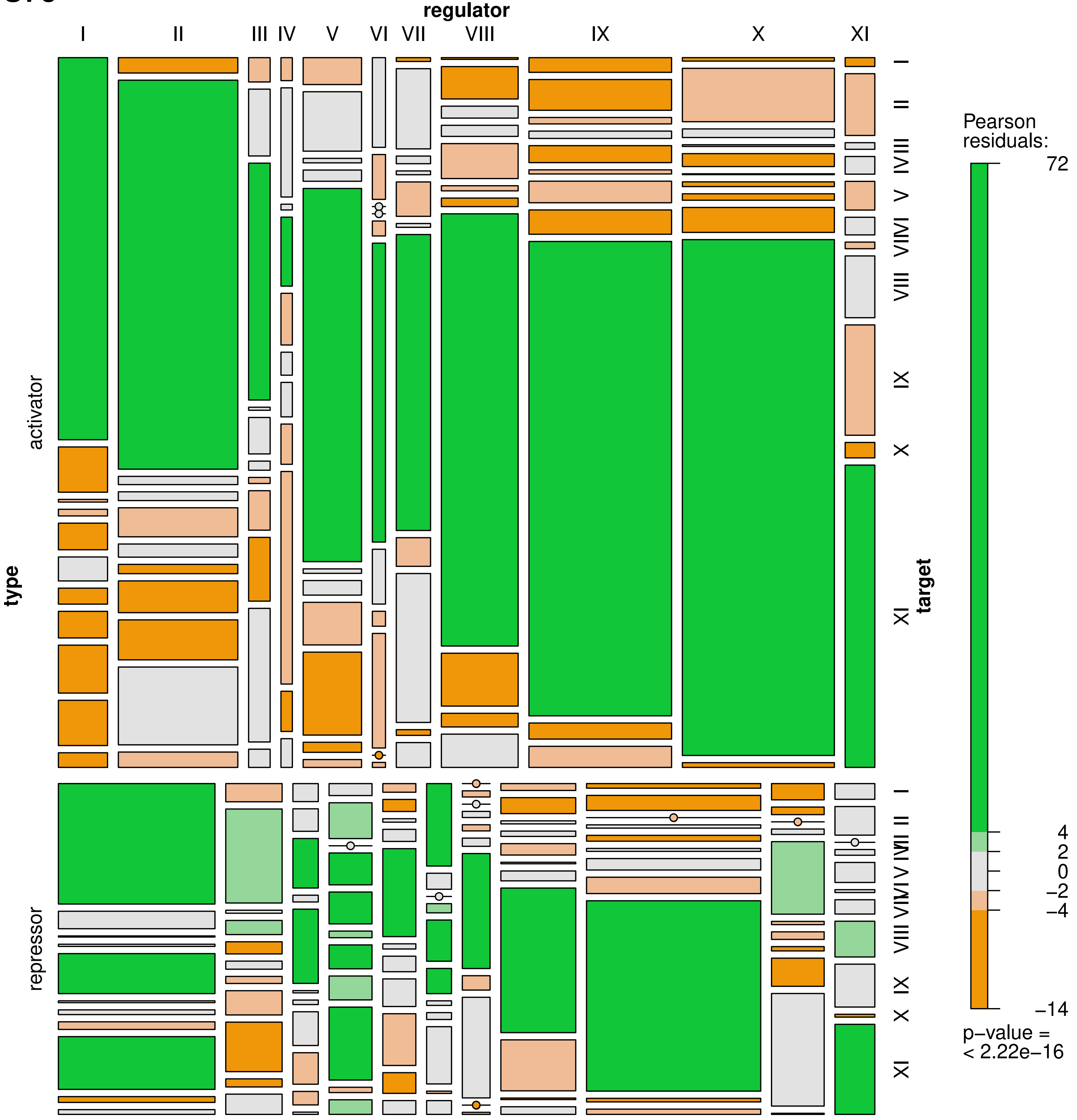
regulator
I
II
III
IV
V
VI
VII
VIII
IX
X
XI
type
activator
repressor
target
Pearson residuals:
72
4
2
0
−2
−4
−14
p−value =
< 2.22e−16

Fig. S7.
Filtered set of predicted direct and indirect DEK1 targets.

S7a. Alluvial plot depicting the distribution of the filtered, predicted direct and indirect DEK1 targets among the 11 subnetworks. Color-coding of bands reflects directionality of deregulation patterns in the mutant lines (see Fig. 1f for details). The green bands represent unaffected upstream TFs predicted to control the significantly deregulated target genes.

S7b. Global network of the filtered, predicted direct and indirect DEK1 targets. Network plot of the 4,125 direct and indirect DEK1 targets. Nodes are color-coded by subnetwork affiliation. Node size is relative to the nodes' local reaching centrality (master regulator TFs have largest). Predicted direct DEK1 cleavage targets are depicted as triangles. Indirect DEK1 targets as circles. Edges are color-coded by deregulation pattern i.e. type of DEK1-controlled regulatory interaction: *repressor targets* (red) and *activator targets* (blue).

S7c. Distribution of intra- and inter-subnetwork regulatory interactions controlled by DEK1. Mosaic plot or Marimekko diagram depicting the three-way cross-tabulation of three factorial variables: Left y-axis: mutant deregulation pattern i.e. type of regulatory interaction *repressor targets* and *activator targets*. Top x-axis: regulator subnetwork affiliation. Right x-axis: target subnetwork affiliation. Box size relative to the number of edges. Boxes are colored based on Pearson residuals from a significant $\chi 2$ test of the cross-table (green: enrichment; orange: under-representation).

S8a

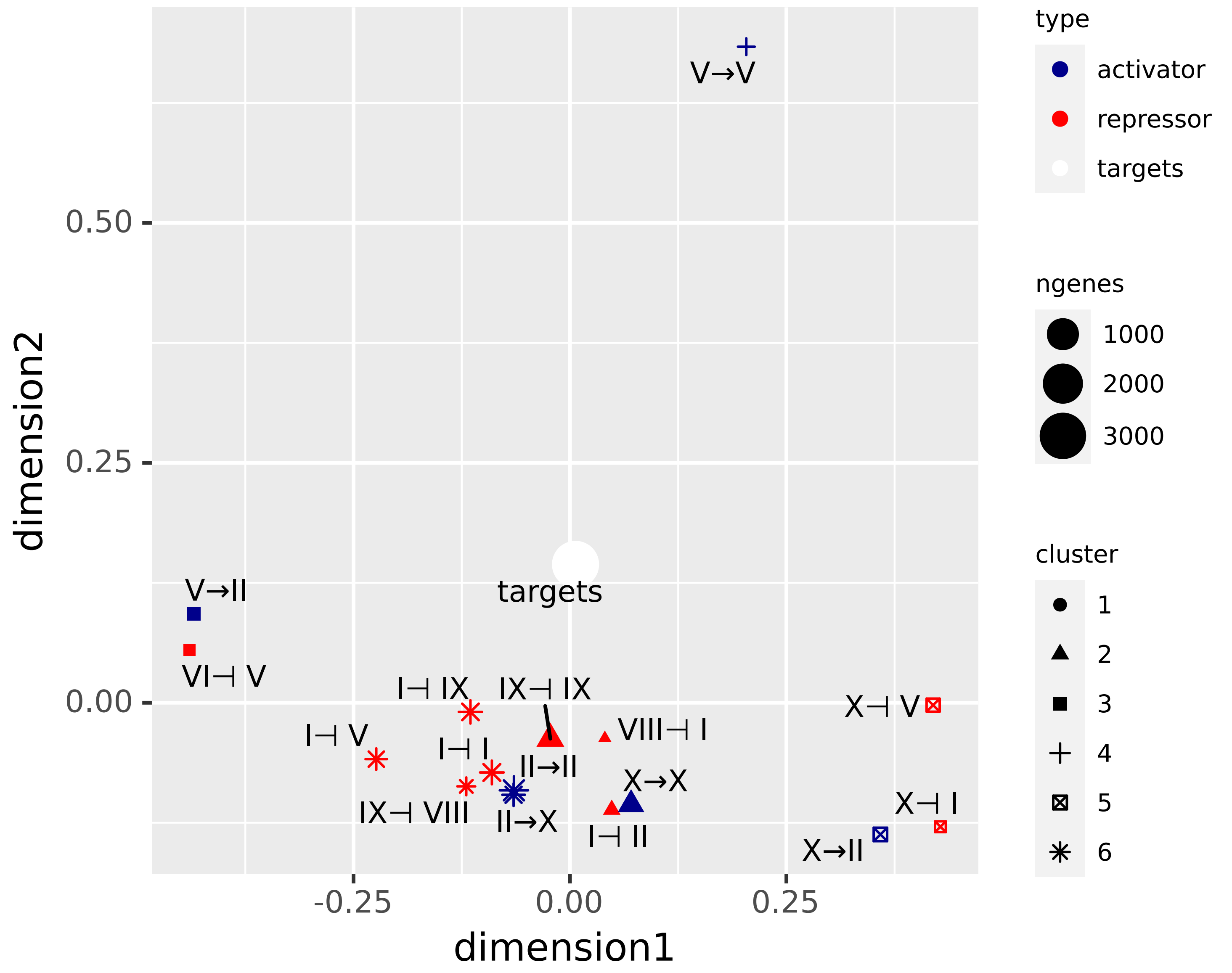

type
activator
repressor
targets
ngenes
1000
2000
3000
cluster
1
2
3
4
5
6
dimension2
dimension1
0.50
0.25
0.00
-0.25
0.00
0.25
V→V
V→II
VI⊣ V
targets
I⊣ IX
IX⊣ IX
I⊣ V
I⊣ I
II→II
VIII⊣ I
X→X
IX⊣ VIII
II→X
I⊣ II
X⊣ V
X⊣ I
X→II

S8b

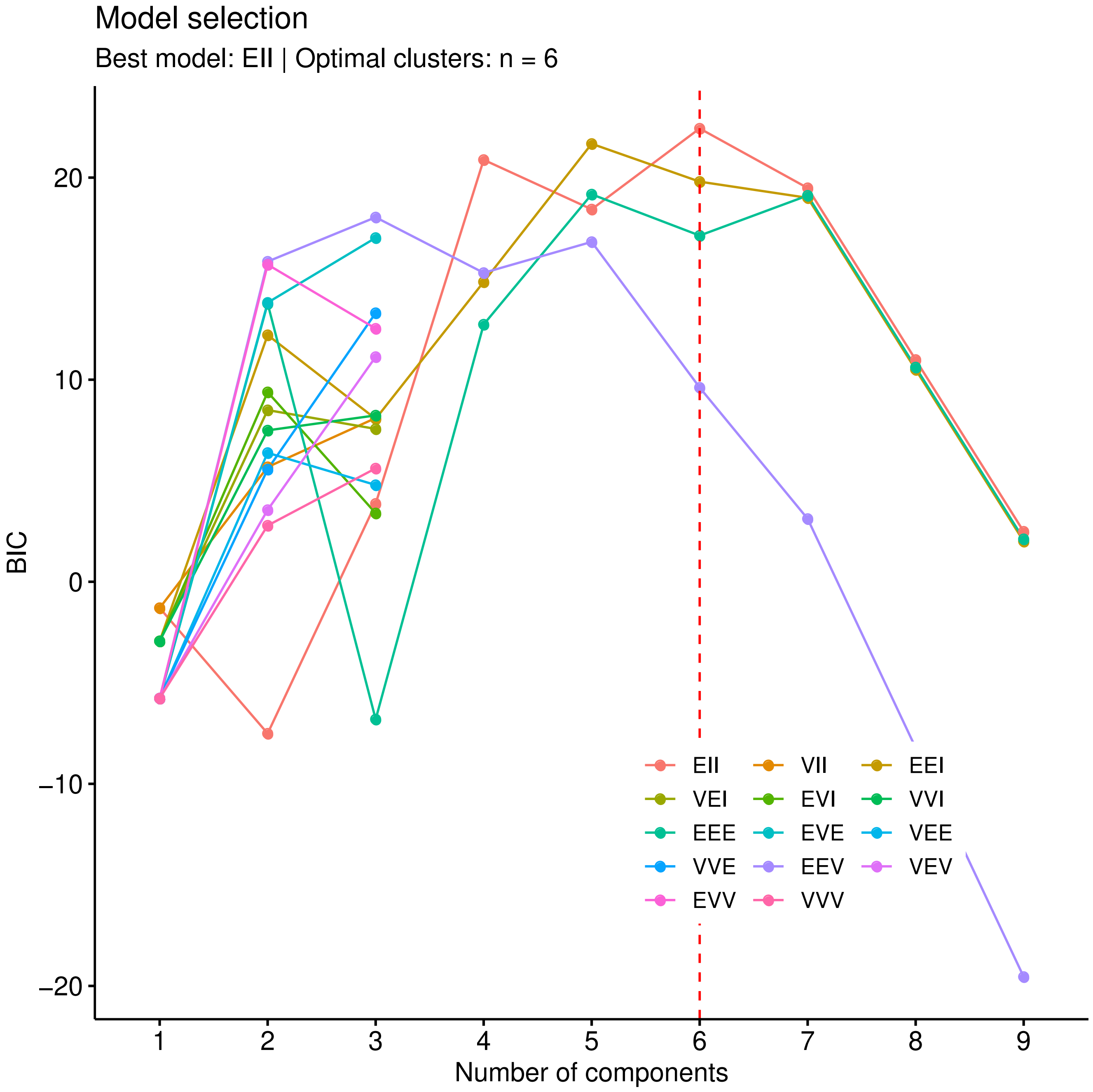
Model selection
Best model: EII | Optimal clusters: n = 6
BIC
Number of components
20
10
0
−10
−20
1
2
3
4
5
6
7
8
9
EII
VII
EEI
VEI
EVI
VVI
EEE
EVE
VEE
VVE
EEV
VEV
EVV
VVV

S8c

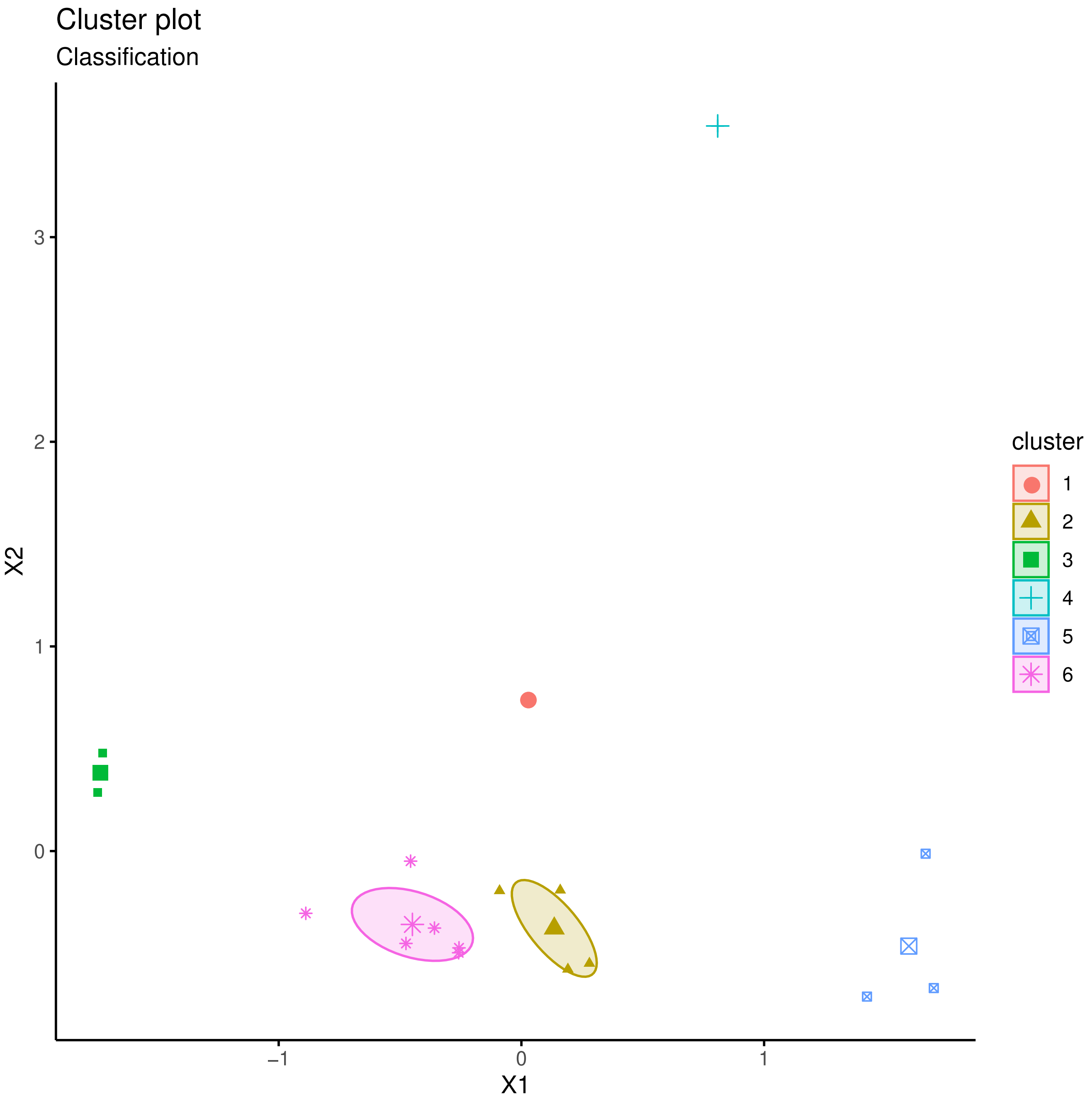

S8d

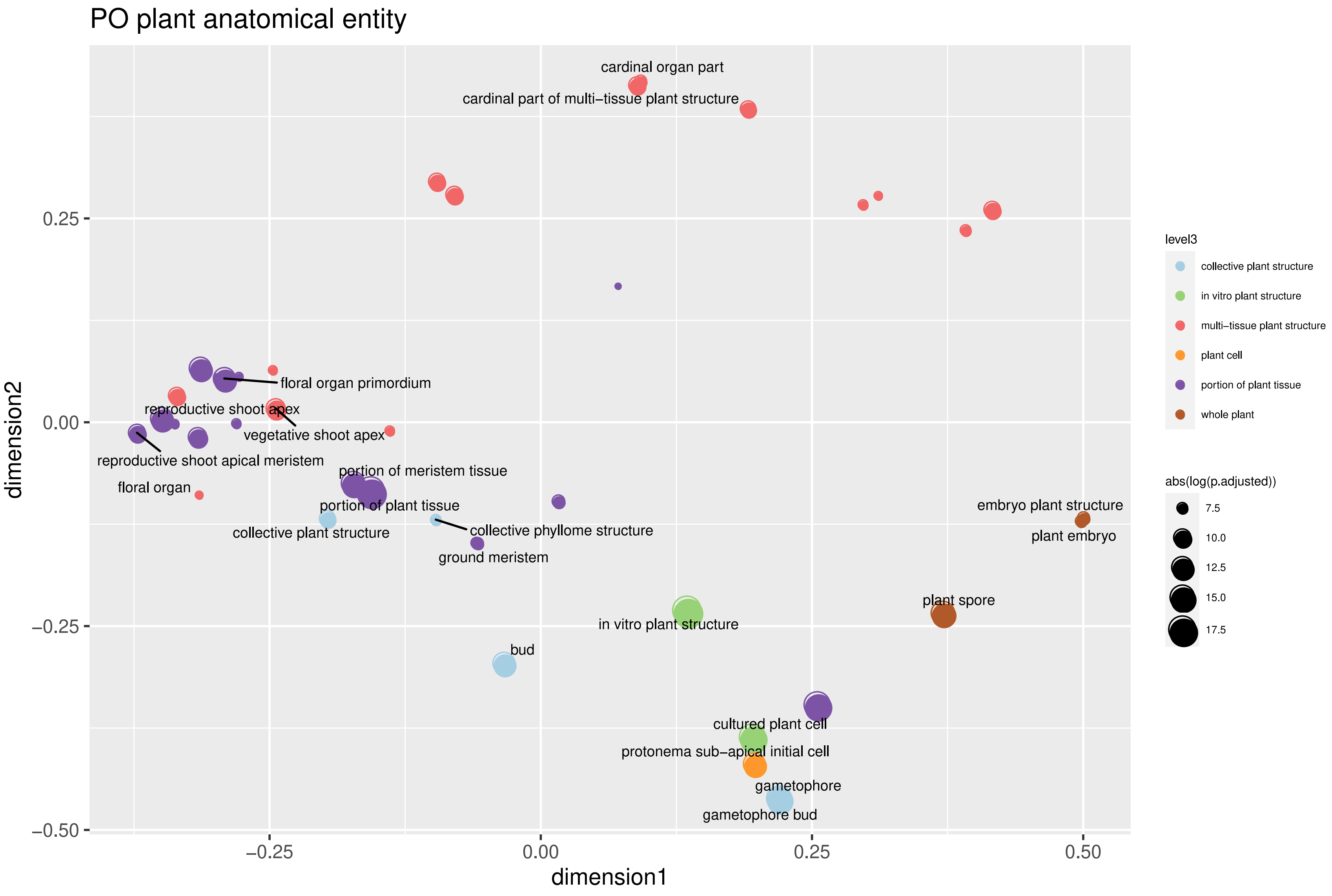
PO plant anatomical entity
cardinal organ part
cardinal part of multi−tissue plant structure
floral organ primordium
reproductive shoot apex
vegetative shoot apex
reproductive shoot apical meristem
portion of meristem tissue
floral organ
portion of plant tissue
collective plant structure
collective phyllome structure
ground meristem
embryo plant structure
plant embryo
plant spore
in vitro plant structure
bud
cultured plant cell
protonema sub−apical initial cell
gametophore
gametophore bud
dimension2
dimension1
0.25
0.00
−0.25
−0.50
0.50
level3
collective plant structure
in vitro plant structure
multi−tissue plant structure
plant cell
portion of plant tissue
whole plant
abs(log(p.adjusted))
7.5
10.0
12.5
15.0
17.5

S8e

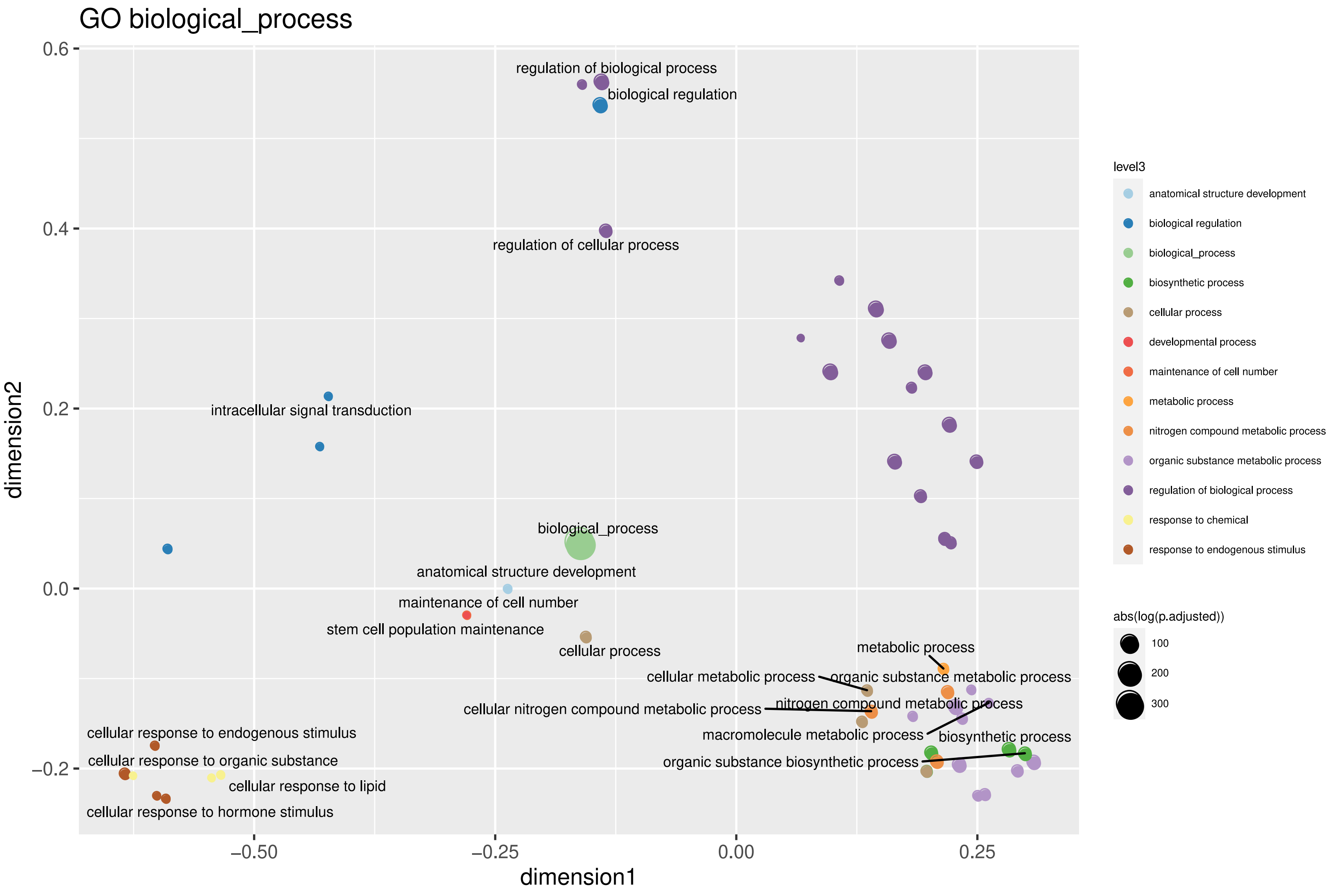

GO biological_process
regulation of biological process
biological regulation
regulation of cellular process
intracellular signal transduction
biological_process
anatomical structure development
maintenance of cell number
stem cell population maintenance
cellular process
metabolic process
cellular metabolic process
organic substance metabolic process
cellular nitrogen compound metabolic process
nitrogen compound metabolic process
macromolecule metabolic process
biosynthetic process
organic substance biosynthetic process
cellular response to endogenous stimulus
cellular response to organic substance
cellular response to lipid
cellular response to hormone stimulus
dimension1
dimension2
0.6
0.4
0.2
0.0
−0.2
−0.50
−0.25
0.00
0.25
level3
anatomical structure development
biological regulation
biological_process
biosynthetic process
cellular process
developmental process
maintenance of cell number
metabolic process
nitrogen compound metabolic process
organic substance metabolic process
regulation of biological process
response to chemical
response to endogenous stimulus
abs(log(p.adjusted))
100
200
300

Fig. S8.
Functional characterization of direct and indirect DEK1 targets by ontology analysis.

S8a. Semantic similarity of DEK1-controlled regulatory interactions. Multidimensional scaling plot of a distance matrix derived by comparing enriched GO biological process terms between the sets of DEK1-controlled regulatory interactions within and between subnetworks using semantic similarity. Sets are depicted as symbols that represent one of six clusters of similar concepts and color-coded by deregulation pattern (DEK1-controlled *repressor* [red] or *activator* [blue] *target*). Point size is relative to the total number of genes in the set.

S8b. Model-based clustering of DEK1-controlled regulatory interactions. Line plot depicting the Bayesian Information Criterion vs. the number of resulting clusters (components) using different clustering models. The EII model with n = 6 resulted in the optimal BIC.

S8c. Optimal model-based clustering (EII model) of DEK1-controlled regulatory interactions. Cluster scatter plot of a multidimensional scaling plot of the semantic similarity/distance matrix (see Panel S8a of the optimal EII model clustering analysis. Points are color-coded by cluster affiliation. Clusters with more than one gene set are depicted with ellipses depicting the variation around the cluster center (larger symbol of same color).

S8d. and S8e. Automatically inferred representative key concepts of enriched (d) PO anatomical entity and (e) GO biological process for DEK1-controlled regulatory interactions. Multidimensional scaling plot of a distance matrix derived by comparing enriched ontology terms from the respective ontology partition for the gene sets of DEK1-controlled regulatory interactions. All enriched terms are represented by a point that is scaled by the number of genes with that term in the set. For groups of similar concepts (aggregated by level 3 ontology terms), terms were ranked in ascending order by information content and log FDR value of term enrichment.

S9

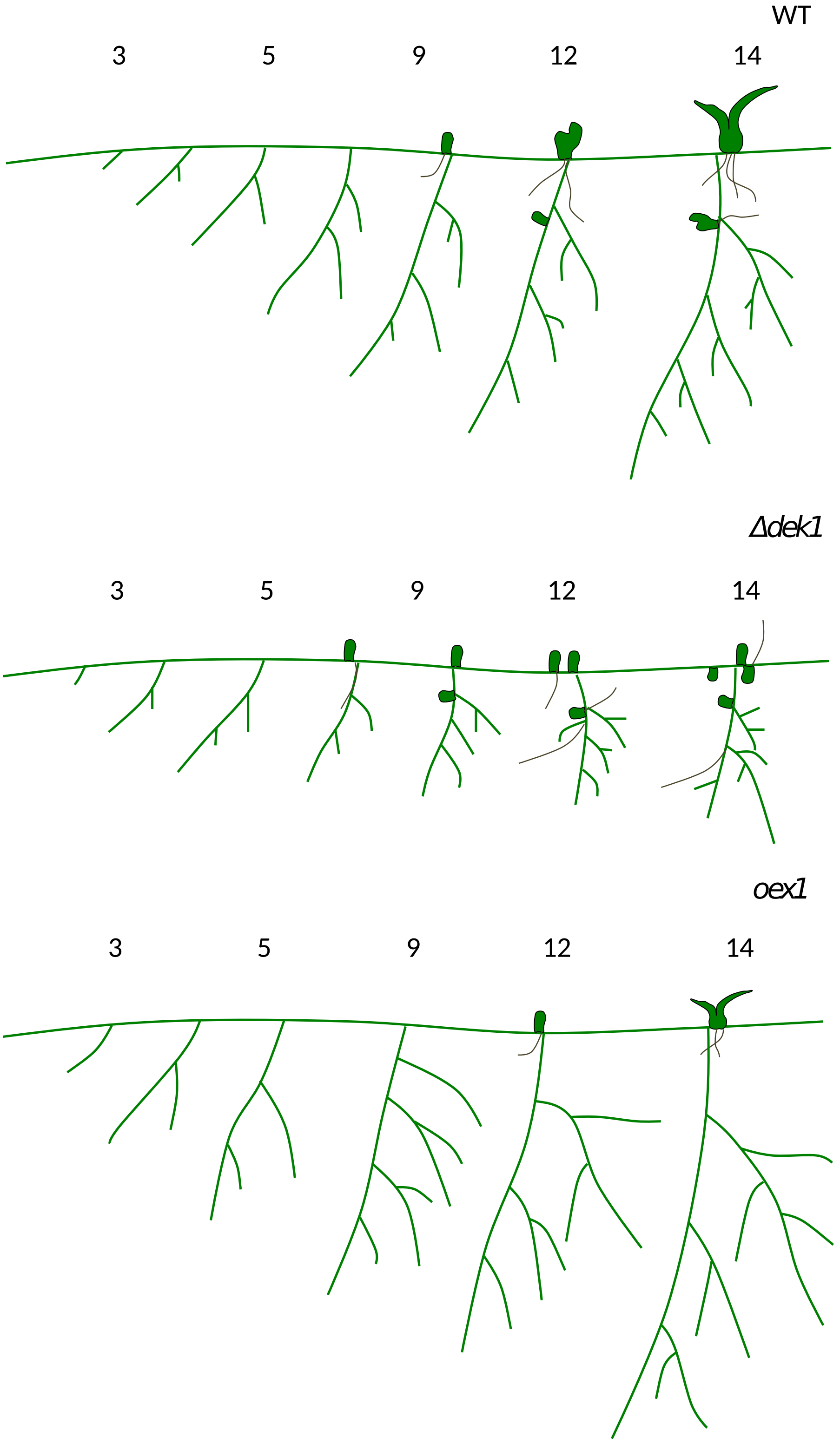

	primary filaments	side branches	bud initiation	gametophore development
Δdek1	normal	reduced elongation	increased	arrested
oex1	elongated	increased elongation	reduced	normal

Fig. S9.

Major phenotypes of the *DEK1* deletion and overexpression mutants. Schematic overview of *P. patens* gametophyte development in wild type (WT), *DEK1* deletion mutant (*Δdek1*) and DEK1 Linker-Calpain overexpressing line (*oex1*). Numbers indicate days after culture initiation. Primary filament is oriented horizontally with side-branches progressively forming secondary filaments positioned vertically. *Δdek1* mutant shows reduced elongation of secondary filaments and accelerated gametophore bud initiation when compared to WT. Developmentally arrested *Δdek1* buds are often formed in clusters. In contrast to *Δdek1*, *oex1* shows increased elongation of secondary filaments and delayed gametophore bud initiation.

S10

a

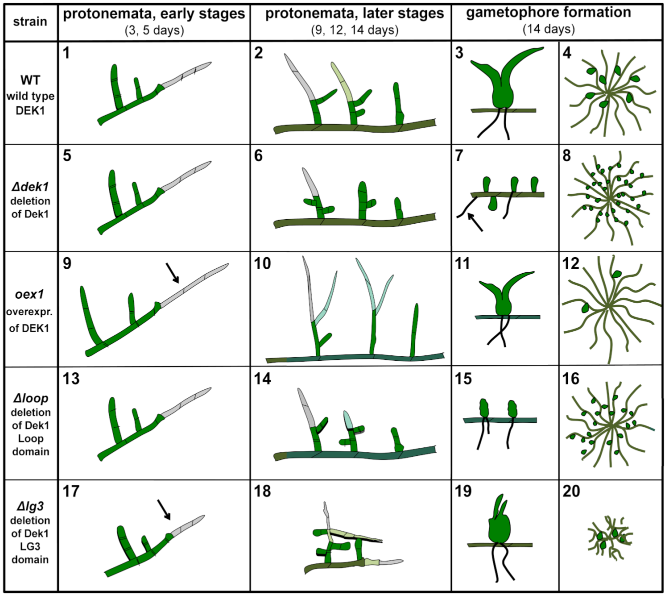

b

mutant phenotype	displayed in cell# in panel a: normal	altered
colony size reduced	4, 8, 16	20
elongation primary caulonema reduced	1, 5, 13	17
number buds per filament high	4, 12, 20	8, 16
percents filaments with buds low	4	12, 20
gametophore formation arrested	3	7, 15
development of phyllids delayed	3	11
gametophore formation delayed	3	11
percents filaments with buds high	4	8, 16
elongation secondary caulonema increased	2	10
colony size enlarged	4, 8, 16	12
rhizoid formation delayed	3, 15, 19	11
elongation primary caulonema increased	1, 5, 13	9
elongation secondary caulonema reduced	2	6, 14,18
rhizoid formation ectopic	3, 15, 19	7
protonemata branching aberrant	2, 6,10,14	18
development of phyllids aberrant	3	19
gametophore formation aberrant	3	20

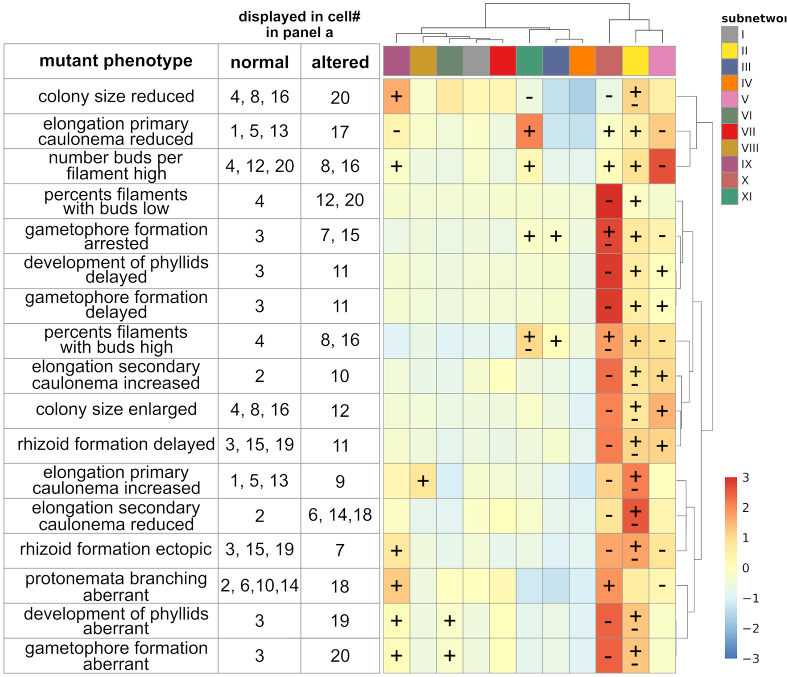

c

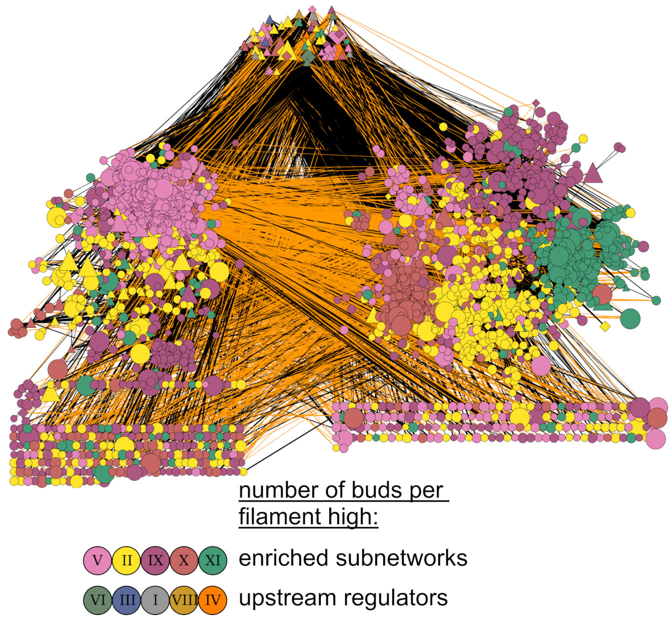

d

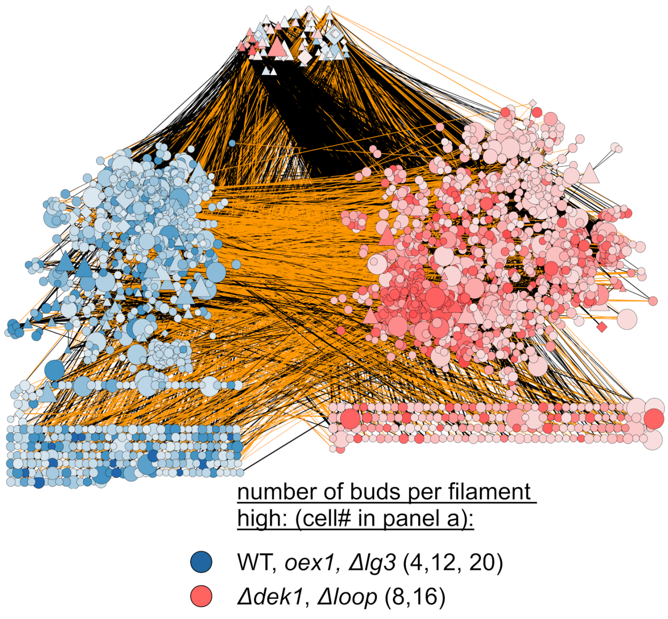

S10e

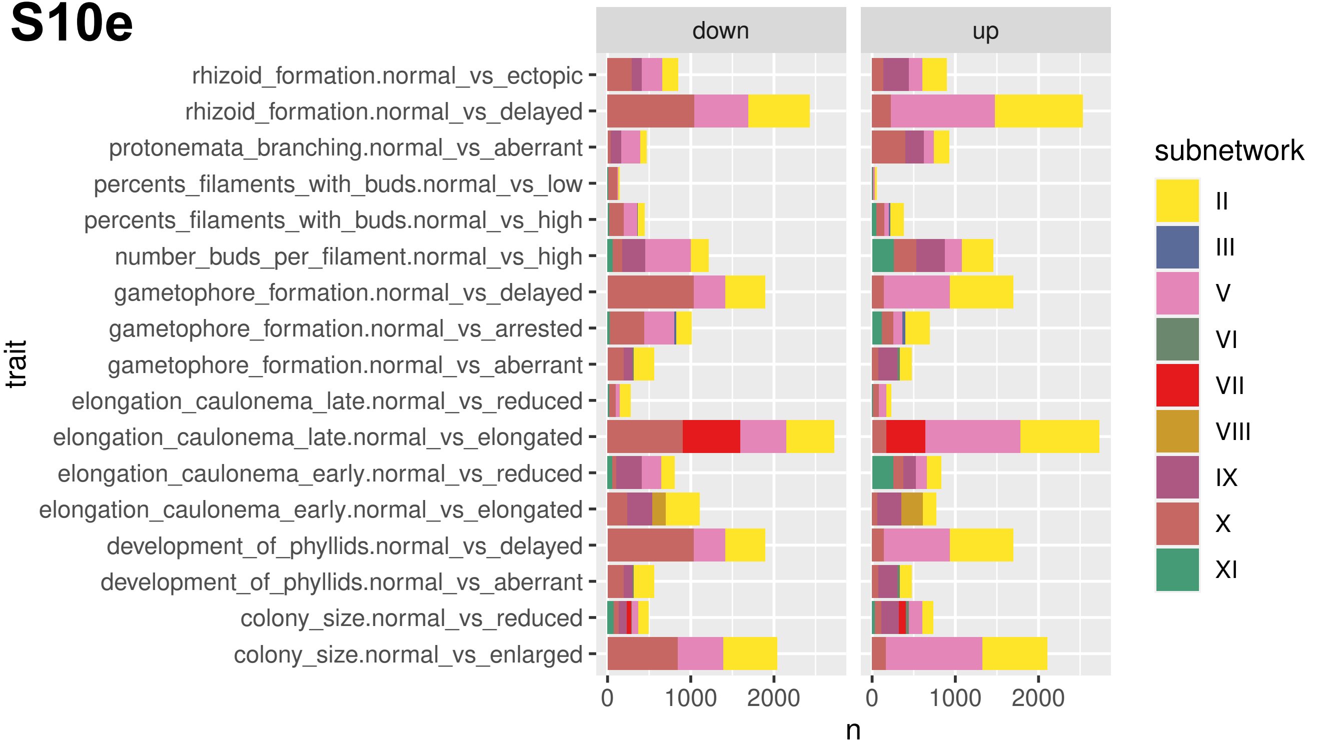
down
up
rhizoid_formation.normal_vs_ectopic
rhizoid_formation.normal_vs_delayed
protonemata_branching.normal_vs_aberrant
percents_filaments_with_buds.normal_vs_low
percents_filaments_with_buds.normal_vs_high
number_buds_per_filament.normal_vs_high
gametophore_formation.normal_vs_delayed
gametophore_formation.normal_vs_arrested
gametophore_formation.normal_vs_aberrant
elongation_caulonema_late.normal_vs_reduced
elongation_caulonema_late.normal_vs_elongated
elongation_caulonema_early.normal_vs_reduced
elongation_caulonema_early.normal_vs_elongated
development_of_phyllids.normal_vs_delayed
development_of_phyllids.normal_vs_aberrant
colony_size.normal_vs_reduced
colony_size.normal_vs_enlarged
trait
0
1000
2000
0
1000
2000
n
subnetwork
II
III
V
VI
VII
VIII
IX
X
XI

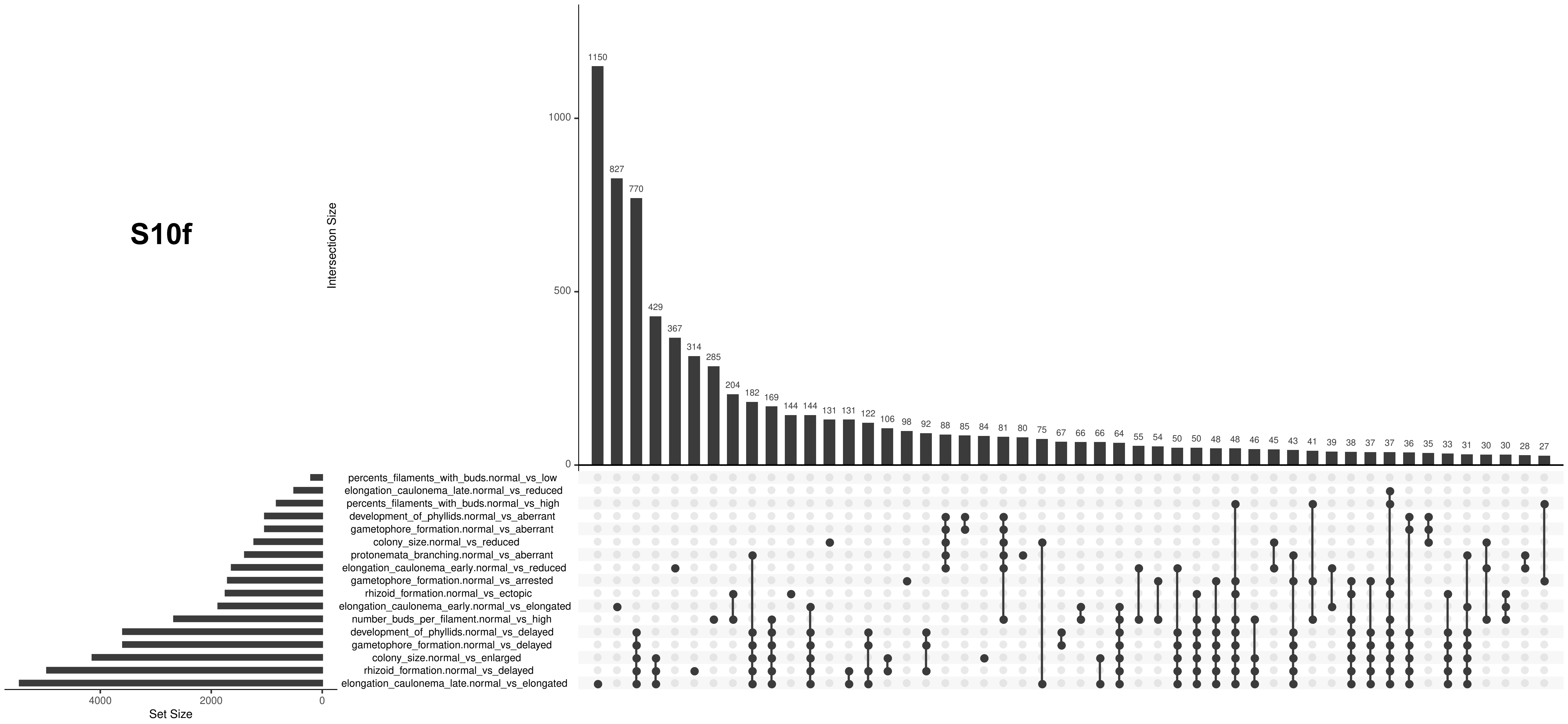
S10f
Intersection Size
1150
827
770
429
367
314
285
204
182
169
144
144
131
131
122
106
98
92
88
85
84
81
80
75
67
66
66
64
55
54
50
50
48
48
46
45
43
41
39
38
37
37
36
35
33
31
30
30
28
27
1000
500
0
percents_filaments_with_buds.normal_vs_low
elongation_caulonema_late.normal_vs_reduced
percents_filaments_with_buds.normal_vs_high
development_of_phyllids.normal_vs_aberrant
gametophore_formation.normal_vs_aberrant
colony_size.normal_vs_reduced
protonemata_branching.normal_vs_aberrant
elongation_caulonema_early.normal_vs_reduced
gametophore_formation.normal_vs_arrested
rhizoid_formation.normal_vs_ectopic
elongation_caulonema_early.normal_vs_elongated
number_buds_per_filament.normal_vs_high
development_of_phyllids.normal_vs_delayed
gametophore_formation.normal_vs_delayed
colony_size.normal_vs_enlarged
rhizoid_formation.normal_vs_delayed
elongation_caulonema_late.normal_vs_elongated
4000
2000
0
Set Size

S10g

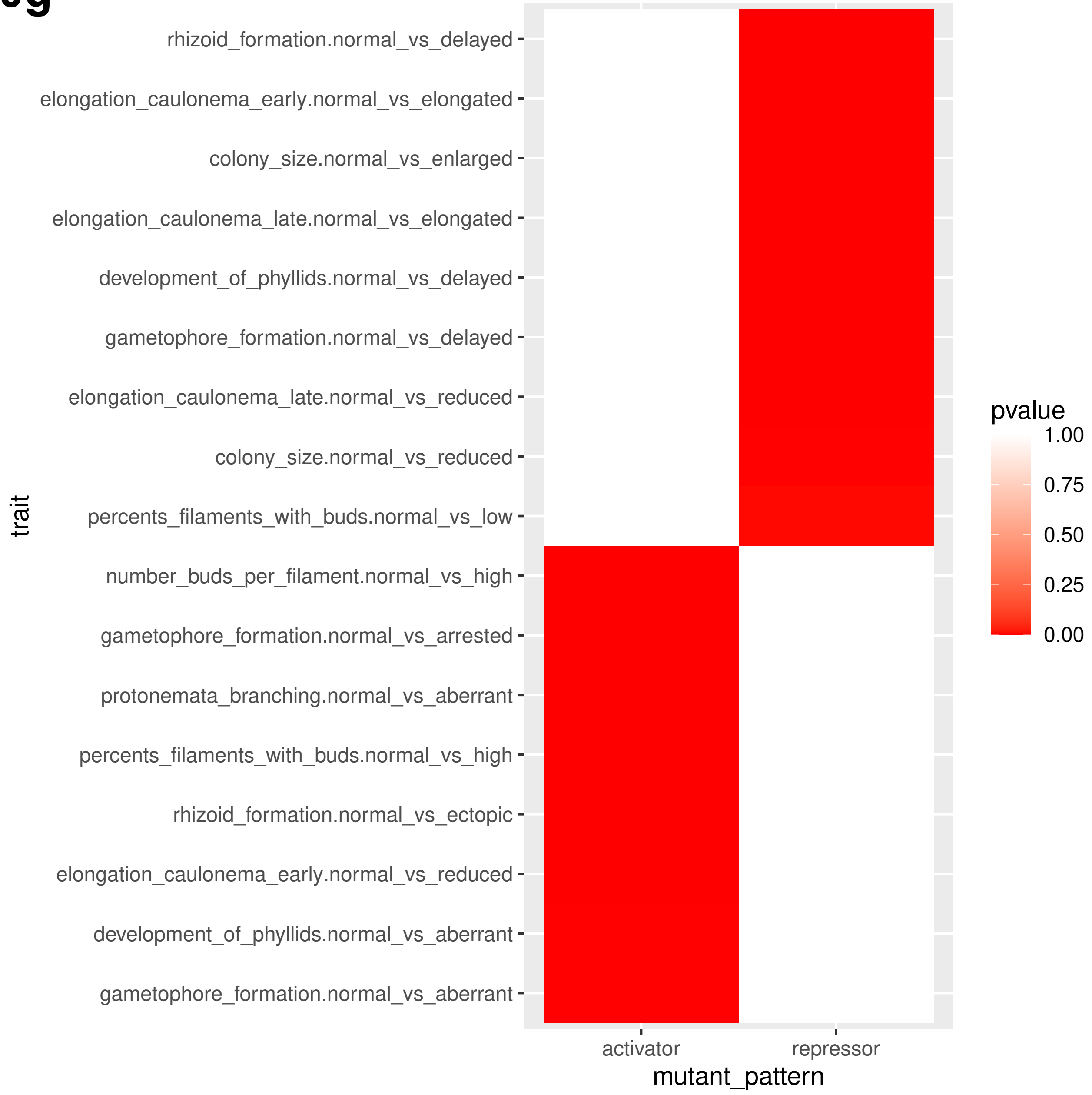

rhizoid_formation.normal_vs_delayed
elongation_caulonema_early.normal_vs_elongated
colony_size.normal_vs_enlarged
elongation_caulonema_late.normal_vs_elongated
development_of_phyllids.normal_vs_delayed
gametophore_formation.normal_vs_delayed
elongation_caulonema_late.normal_vs_reduced
colony_size.normal_vs_reduced
percents_filaments_with_buds.normal_vs_low
number_buds_per_filament.normal_vs_high
gametophore_formation.normal_vs_arrested
protonemata_branching.normal_vs_aberrant
percents_filaments_with_buds.normal_vs_high
rhizoid_formation.normal_vs_ectopic
elongation_caulonema_early.normal_vs_reduced
development_of_phyllids.normal_vs_aberrant
gametophore_formation.normal_vs_aberrant
trait
activator
repressor
mutant_pattern
pvalue
1.00
0.75
0.50
0.25
0.00

S10h

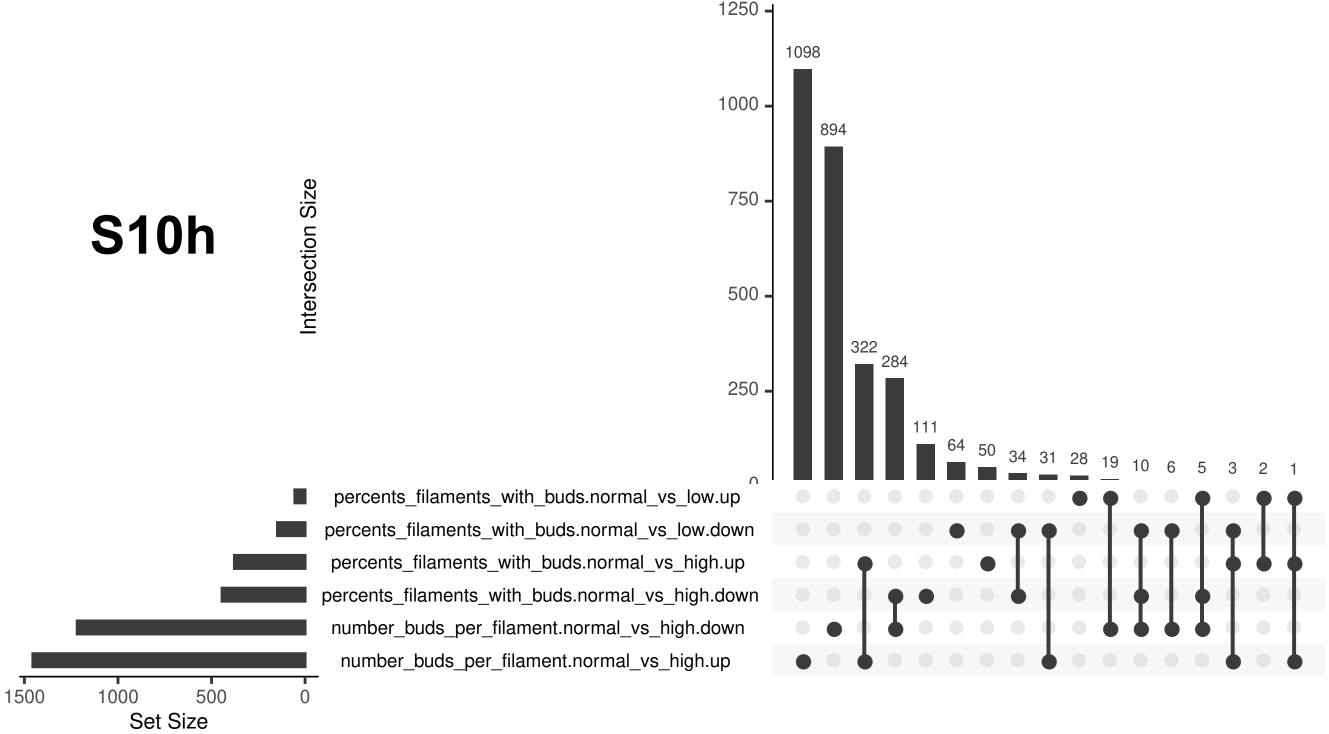

Intersection Size
1250
1000
750
500
250
0
1098
894
322
284
111
64
50
34
31
28
19
10
6
5
3
2
1
percents_filaments_with_buds.normal_vs_low.up
percents_filaments_with_buds.normal_vs_low.down
percents_filaments_with_buds.normal_vs_high.up
percents_filaments_with_buds.normal_vs_high.down
number_buds_per_filament.normal_vs_high.down
number_buds_per_filament.normal_vs_high.up
1500
1000
500
0
Set Size

S10i

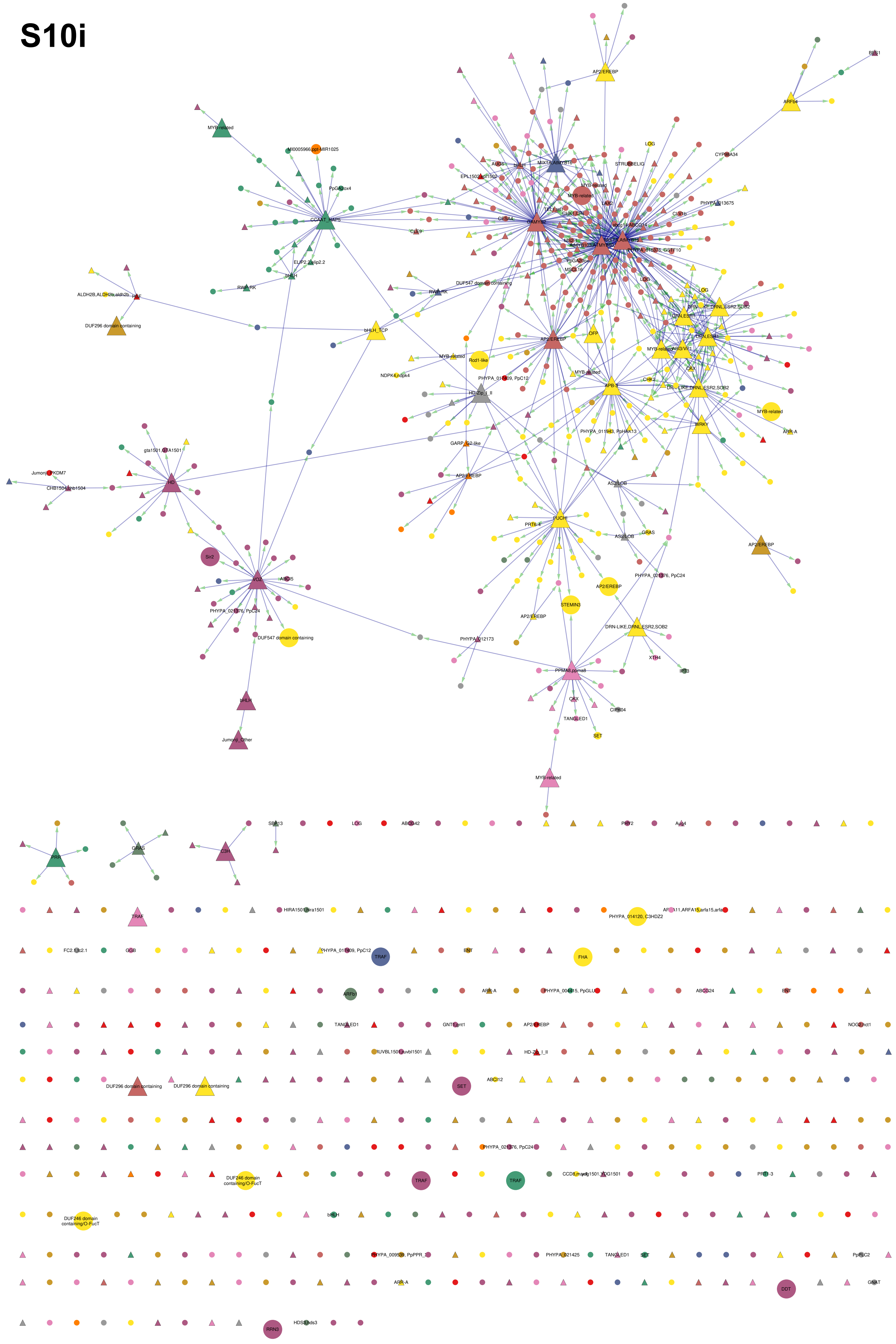

AP2/EREBP
ARFb4
BES1
MYB-related
MI0005966,ppt-MIR1025
PpGA2ox4
CCAAT_HAP5
ELIP2.2,elip2.2
bHLH
RWP-RK
ALDH2B,ALDH2b,aldh2b
HSF
DUF296 domain containing
bHLH_TCP
CLE9
CESA4
GAMYB2
MIXTA,AtMYB16
AUG5
EPL1502,epl1502
LOG
STRUBBELIG
CYP98A34
PHYPA_013675
CLV1b
LAX2
TEL1,tel1
CHK1,CRI
MS2-1
PpGA2ox4
MSC1.16
DUF547 domain containing
Rod1-like
NDPK4,ndpk4
OFP
PHYPA_017409, PpC12
HD-Zip_I_II
CHK2
APB-3
CKX
DRN,ESR1
ABI3/VP1
WRKY
ARR-A
PHYPA_011943, PpHAK13
GARP_G2-like
AS2/LOB
gta1501,GTA1501
Jumonji_PKDM7
CHB1504,chb1504
HD
Sir2
VOZ
ABCI5
PHYPA_021376, PpC24
DUF547 domain containing
bHLH
Jumonji_Other
PRT6-4
FUCHI
GRAS
STEMIN3
DRN-LIKE,DRNL,ESR2,SOB2
PHYPA_012173
XTH4
PPMA8,ppma8
CKX
CIPK04
TANGLED1
SET
PRR
GRAS
C3H
SBP13
ABCG42
PHY2
TRAF
HIRA1501,hira1501
PHYPA_014120, C3HDZ2
FC2.1,fc2.1
GGB
ENT
FHA
ARFb1
PHYPA_004415, PpGLU
ABCG24
GNT1,gnt1
NOG2,hct1
RUVBL1501,ruvbl1501
HD-Zip_I_II
DUF296 domain containing
SET
ABCI12
DUF246 domain containing/O-FucT
CCD8,maxd1501,YDG1501
PRT1-3
PHYPA_009539, PpPPR_3
PHYPA_021425
PpPLC2
ARR-A
DDT
GNAT
RRN3
HDS3,hds3

S10j

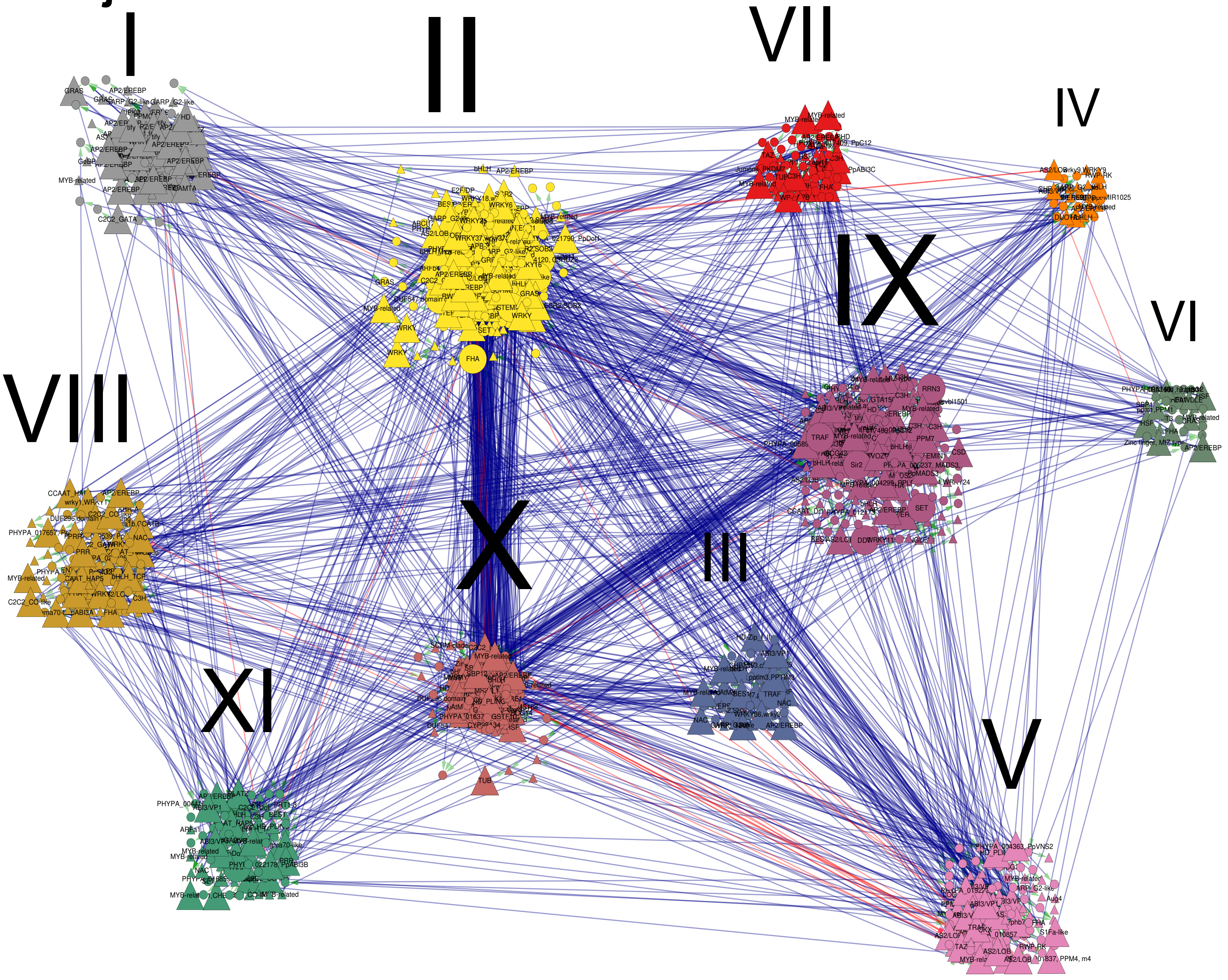

S10k

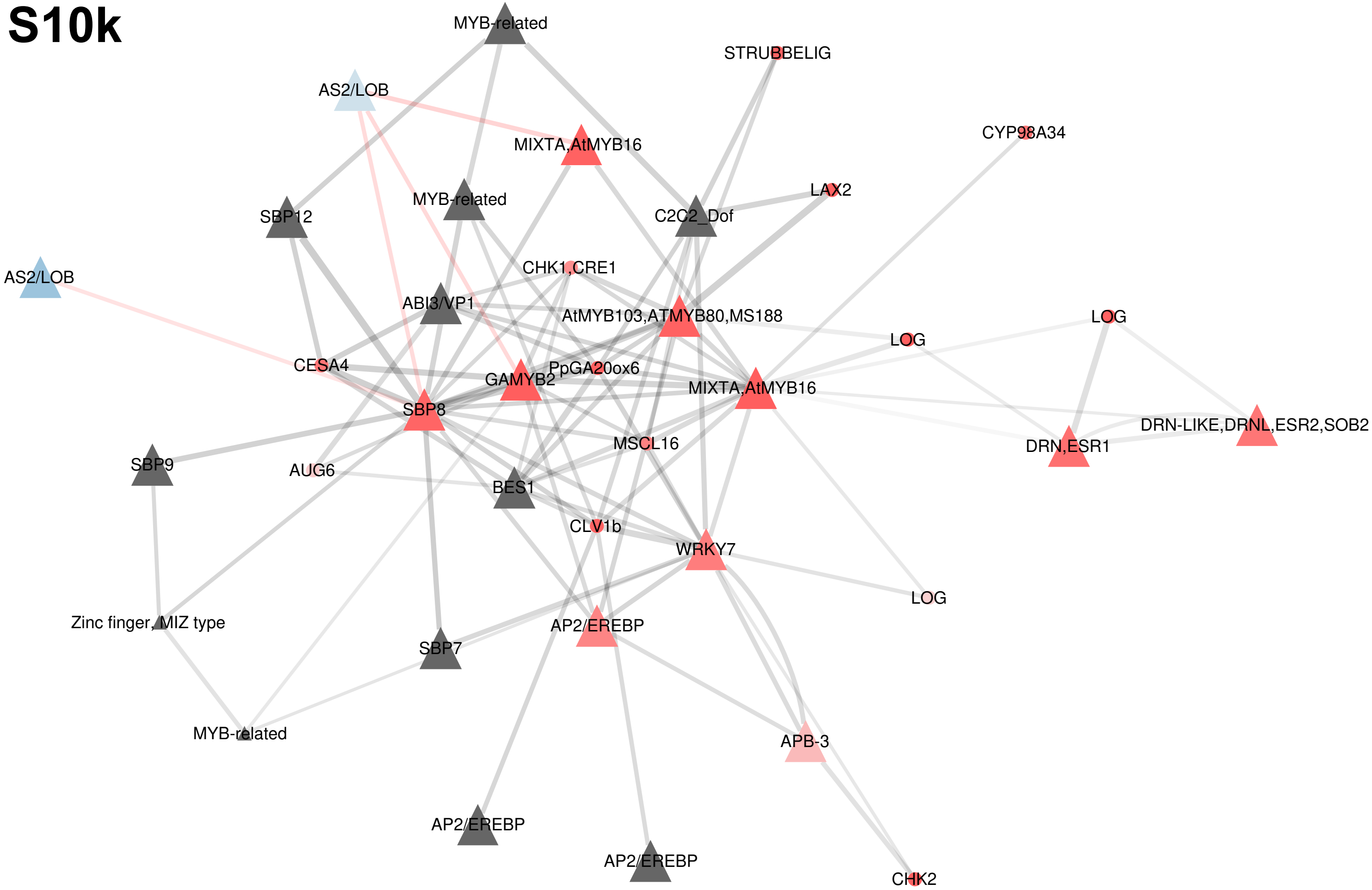

MYB-related
STRUBBELIG
AS2/LOB
CYP98A34
MIXTA,AtMYB16
LAX2
MYB-related
SBP12
C2C2_Dof
AS2/LOB
CHK1,CRE1
ABI3/VP1
AtMYB103,ATMYB80,MS188
LOG
LOG
CESA4
PpGA20ox6
GAMYB2
MIXTA,AtMYB16
SBP8
DRN-LIKE,DRNL,ESR2,SOB2
MSCL16
DRN,ESR1
SBP9
AUG6
BES1
CLV1b
WRKY7
LOG
Zinc finger, MIZ type
AP2/EREBP
SBP7
MYB-related
APB-3
AP2/EREBP
AP2/EREBP
CHK2

S10

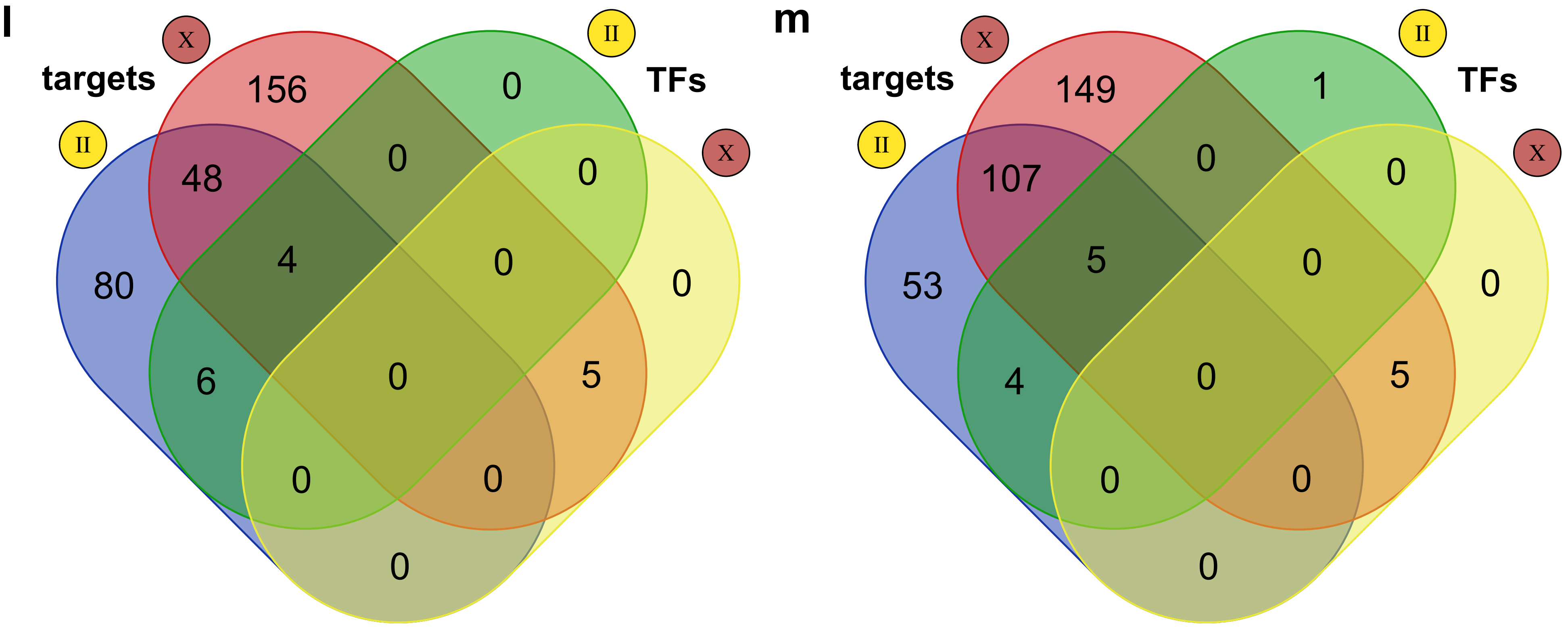
l
targets
TFs
X
II
II
X
156
0
48
0
0
80
4
0
0
6
0
5
0
0
0
m
targets
TFs
X
II
II
X
149
1
107
0
0
53
5
0
0
4
0
5
0
0
0

Supplementary Fig. S10.

Deciphering complex phenotypes by Factorial Differential Gene Expression Network Enrichment Analysis (FDGENEA).

S10a. Translation of the complex DEK1 phenotype to phenotypic factors. Graphical summary of the quantified *DEK1* mutant phenotypes and translation into binary phenotypic factors (first table column in Panel S10b. Cell numbers are used throughout the other panels to refer to a specific phenotypic trait.

S10b. Contrasting phenotypic factors in Differential Gene Expression Network Enrichment Analysis. Numbers refer to cell numbers containing drawings of the respective phenotype in Panel S10a. Symbols in the heat map cells represent directionality of differential gene expression for each component trait for each of the subnetworks: + depicts enrichment of up-regulated genes. - depicts enrichment of down-regulated genes. Heatmap fill-color of each cell represents the z-score-scaled, overall enrichment of DEGs (irrespective of their directionality) for each phenotypic factor. Subnetworks X, II and V are predominantly enriched for regulons affecting 2D-to-3D phenotypes.

S10c. Enriched subnetworks and upstream regulators associated with high number of buds per filament – subnetwork colors. Nodes are color-coded by subnetworks. Node sizes relative to the cumulative, absolute fold-change of deregulation of the respective gene and any possible target gene in the mutants. Node shapes: TFs depicted as triangles, TRs as diamonds, miRNAs as inverted triangles and targets as circles. Edge colors: positive correlation of connected nodes in DEK1 RNASeq data (black) and negative correlation coefficient (orange).

S10d. Enriched subnetworks and upstream regulators associated with high number of buds per filament – log2foldchanges. Same graph and attributes as Panel S10c, but color-coded by directionality of DGE analysis (intensity gradient relative to log-fold change). The three groups of nodes depicted in Panels S10c and S10d correspond to genes that are (R) positively associated with a high number of buds (i.e. are up-regulated in *Δdek1* and *Δloop*; right group), (L) display a negative association (i.e. up-regulated in WT, *oex1* and *Δlg3*[18]; left group and down-regulated in *Δdek1*[8] and *Δloop*[19]) or (T) upstream regulators without significant change in gene expression with respect to this phenotype (top group). This clustering is also evident in the type of connections between these groups – especially the two major assemblages harbor many negative regulatory interactions (orange color of links between them). Overall, while subnetwork V dominates the left group, the right group of nodes is more diverse and consists of subnetworks X, XI and IX. Subnetwork II is prominent in both groups. This pattern gives important insight into the implementation of this developmental phase transition – a predominantly negative interaction between the regulatory toolkit driving gametophore development (X) and the gene complement of the primary filament (V; chloronema). This transition between cell states is brokered by the subnetwork II, which is also implementing the other types of secondary filaments (caulonema and rhizoids). Subnetworks IX and XI seem to act in conjunction.

S10e. FDGENEA gene sets. These sets comprise genes that are significantly (LRT q-value < 0.1) associated with any of the phenotypic factors and fall within any of the enriched subnetworks (see Panels S10a and S10b).

S10f. Top50 intersections between the FDGENEA gene sets. UpSetR plot depicting the 50 largest non-overlapping FDGENEA gene sets (Panels S10b and S10e).

S10g. The indirect DEK1 target types display specific enrichment patterns among the FDGENEA sets. Subset of the FDGENEA gene sets that is predicted to be indirectly targeted by DEK1, i.e. controlled by either an activator or a repressor TF that is directly cleaved by the DEK1 calpain. Enrichment of the respective DEK1 target type in each of the FDGENEA gene sets was tested using the *enrichment_test* method from the RVenn package. P-values for both target types were used to cluster the sets by hierarchical clustering using the *ward.D2* method in R.

S10h. All intersections of gene sets associated with traits involving the number of buds per filament i.e. bud initiation. UpSetR plot depicting non-overlapping FDGENEA gene sets (Panels S10b and S10e).

S10i. Network of DEK1-controlled, *overbudding* up-regulated genes. Network plot depicting FDGENEA gene set with significant, positive association to the *number of buds per filament high* trait, predicted to be indirectly or directly controlled by DEK1. Total number of nodes: 901 genes. Node colors based on subnetwork affiliations. Nodes were labeled based on available manual annotations, which are usually indicative of experimental evidence or curation efforts for this study. Edge colors based on mutant expression pattern and directionality of the predicted, DEK1-controlled, regulatory interaction (i.e. blue: *activator targets* and red: *repressor targets*; see Fig 1 and main text for details). Triangular node shape indicates predicted, DEK1-controlled TFs (cleavage targets) and circles their targets. Node sizes relative to overall local reaching network centrality.

S10j. Network of DEK1-controlled, *overbudding* up-regulated genes and their top5 DEK1-controlled regulators. Network plot depicting FDGENEA gene set with significant, positive association to the *number of buds per filament high* trait, predicted to be indirectly controlled by DEK1 and their top5, direct, DEK1-controlled regulators. Total number of nodes: 1,222 genes. The latter were ranked by their overall GENIE3 weight for each target and did not need to be significantly associated with the trait themselves. Node colors based on subnetwork affiliations. Nodes were labeled based on available manual annotations, which are usually indicative of experimental evidence or curation efforts for this study. Edge colors based on mutant expression pattern and directionality of the predicted, DEK1-controlled, regulatory interaction (i.e. blue: *activator targets* and red: *repressor targets*; see Fig 1 and main text for details). Triangular node shape indicates predicted, DEK1-controlled TFs (cleavage targets) and circles their targets. Node sizes relative to overall local reaching network centrality.

S10k. Full regulatory context of CLV1b is enriched for genes involved in flowering plant meristem initiation and maintenance that are positively associated with bud initiation. Network plot depicting the regulatory context of CLV1b, i.e. genes that are predicted to be controlled by the same regulators as CLV1b and TFs. Selected were only genes that are not more than two orders away, i.e. maximally direct targets of regulators of regulators of

CLV1b. Triangular node shapes indicate predicted direct NERD-like calpain cleavage. Node colour scaled according to log2foldchange from the FDGENEA analysis of the overbudding trait (up = positive association = red; down = negative association = blue; grey = LRT q-value >= 0.1).

S10l. Partial overlap between DEK1-controlled AP2 and MYB TFs in subnetworks II. Venn diagram depicting intersections between the target lists of the ten subnetwork II AP2 (APB-3, SEMIN3 as well as orthologs of PUCHI, DRN, DRNL, SEMIN3) and five subnetwork X MYB (GAMYB and orthologs of MYB80 and MIXTA) TFs. Targets were selected from *overbudding* up-regulated, predicted DEK1-controlled regulatory interactions (Fig. S10j) choosing first and second degree outgoing connections of said TFs (Supplementary Data sheet S11; columns B & E).

S10m. Partial overlap between DEK1-controlled AP2 and MYB TFs in subnetworks II and X. Venn diagram depicting intersections between the target lists of the ten subnetwork II AP2 (APB-3, SEMIN3 as well as orthologs of PUCHI, DRN, DRNL, SEMIN3) and five subnetwork X MYB (GAMYB and orthologs of MYB80 and MIXTA) TFs. Targets were selected from the extended *overbudding* up-regulated, predicted DEK1-controlled regulatory interactions that included also the up to top5 DEK-controlled regulators independent of their deregulation pattern with respect to *overbudding* (Fig. S10j) choosing first and second degree outgoing connections of said TFs (Supplementary Data sheet S11; columns C&F).

S11

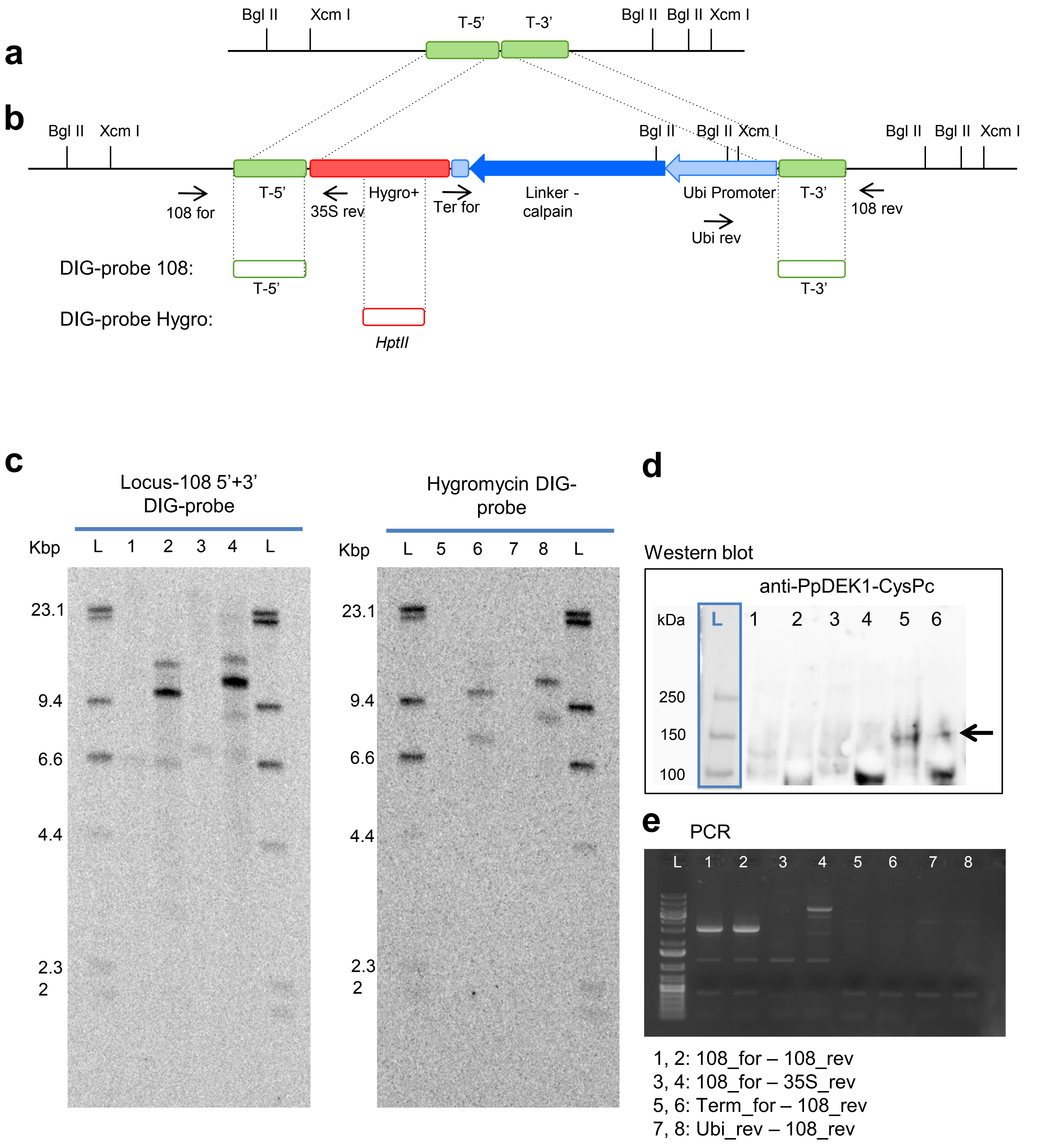
a
Bgl II
Xcm I
T-5’
T-3’
Bgl II
Bgl II
Xcm I
b
Bgl II
Xcm I
Bgl II
Bgl II
Xcm I
Bgl II
Bgl II
Xcm I
T-5’
108 for
35S rev
Hygro+
Ter for
Linker -
calpain
Ubi Promoter
T-3’
108 rev
Ubi rev
DIG-probe 108:
T-5’
T-3’
DIG-probe Hygro:
HptII
c
Locus-108 5’+3’
DIG-probe
Kbp
L 1 2 3 4 L
23.1
9.4
6.6
4.4
2.3
2
Hygromycin DIG-
probe
Kbp
L 5 6 7 8 L
23.1
9.4
6.6
4.4
2.3
2
d
Western blot
anti-PpDEK1-CysPc
kDa
L 1 2 3 4 5 6
250
150
100
e
PCR
L 1 2 3 4 5 6 7 8
1, 2: 108_for – 108_rev
3, 4: 108_for – 35S_rev
5, 6: Term_for – 108_rev
7, 8: Ubi_rev – 108_rev

S11

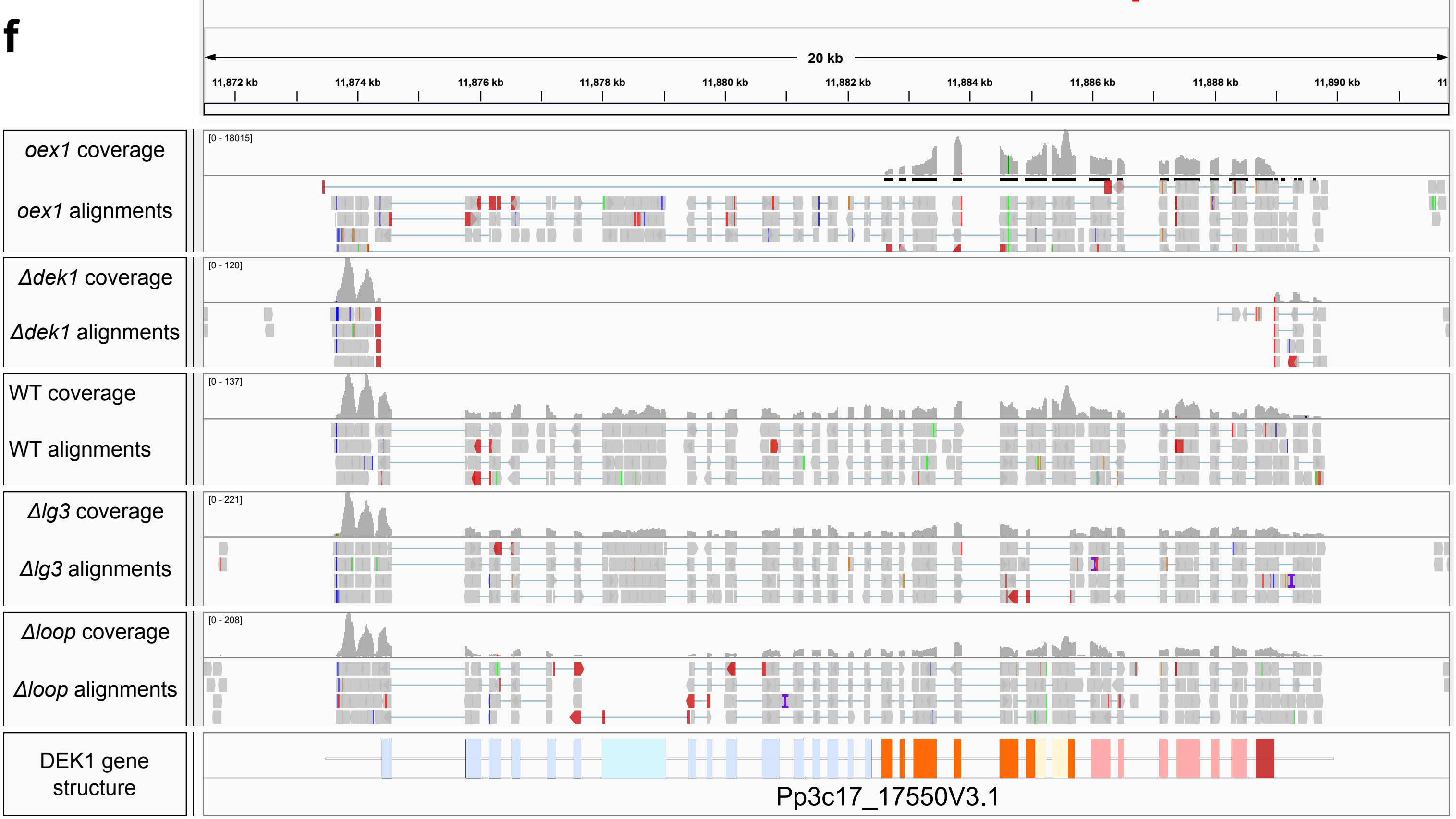

f
Chr17
20 kb
11,872 kb
11,874 kb
11,876 kb
11,878 kb
11,880 kb
11,882 kb
11,884 kb
11,886 kb
11,888 kb
11,890 kb
oex1 coverage
oex1 alignments
[0 - 18015]
Δdek1 coverage
Δdek1 alignments
[0 - 120]
WT coverage
WT alignments
[0 - 137]
Δlg3 coverage
Δlg3 alignments
[0 - 221]
Δloop coverage
Δloop alignments
[0 - 208]
DEK1 gene structure
Pp3c17_17550V3.1

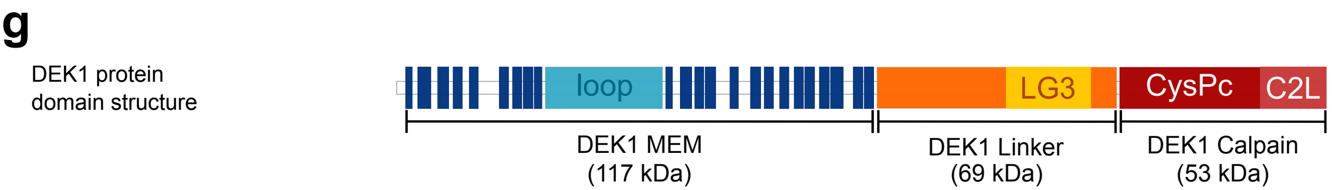

g
DEK1 protein domain structure
loop
LG3
CysPc
C2L
DEK1 MEM (117 kDa)
DEK1 Linker (69 kDa)
DEK1 Calpain (53 kDa)

Supplementary Fig. S11.

Molecular analysis of the linker-calpain overexpressing (*oex1*) strain.

S11a. The *108* locus (T-5', T-3') in the wild type (WT). Schematic representation of 5' and 3' targeting sites (green filled boxes) for the insertion of the PpDEK1 Linker-Calpain overexpression construct at the neutral *108* locus[15] in the *P. patens* WT genotype. Used restriction sites are marked and annotated as ticks.

S11b. Schematic representation of the *108* locus in the *oex1* genotype. Designed targeting of the *Pp*DEK1 Linker-Calpain construct (blue, thick arrows) under a maize ubiquitin promoter (light blue thick arrow) and Terminator (light blue thick box) coupled with a hygromycin-resistance cassette (35S promoter - *HptII* hygromycin resistance gene; red box). Positions of the restriction sites (black ticks), DIG-probes (green and red framed boxes) and primers (black arrows) used in downstream analyses (Figs. S11c, d, e) are marked and annotated accordingly.

S11c. Southern blot analysis *of oex1* transformant. The Southern blot analysis performed with two different restriction enzymes, Bgl II and Xcm I, indicates that *oex1* is a stable transformant. The line *oex1* contains multiple concatenated copies of the *Pp*DEK1 Linker-Calpain construct inserted at a single genomic position, the locus *108*. This insertion pattern represents the majority of the mutants obtained with this transformation procedure in *P. patens*[16] and is not known to induce any phenotype per se. Lanes 1, 3, 5 and 7 contain wild type genomic DNA. Lanes 2, 4, 6 and 8 contain *oex1* genomic DNA. Lanes 1, 2, 5 and 6: Bgl II restriction digestions; lanes 3, 4, 7, 8, 10: Xcm I restriction digestion. Lane L: Lambda phage-HindIII DIG labeled marker.

S11d. Detection of the *Pp*DEK1 Linker-Calpain protein in *oex1*. Western blot detecting *Pp*DEK1 CysPc epitope with polyclonal anti-*Pp*DEK1 CysPc-C2L antibody. L: protein ladder, 1, 2: WT, 3, 4: *Δdek1*, 5, 6: *oex1*. Protein samples in lanes 1, 3, 5 were heated before PAGE, samples 2, 4, 6 were not heated before PAGE. Chemiluminescence was detected followed by imaging of the prestained dual color protein marker (Bio-Rad) on the same membrane. The marker image (lane L; blue frame) was merged with the chemiluminescence scan. The *oex1* line displays a clear signal between the 90 and 150kDa marker size (lanes 5, 6), that is absent in WT (lanes 1, 2) or *Δdek1* (lane 3, 4) and corresponds to the predicted 120kDa Linker-Calpain protein. The bottom part of the membrane scan contains a uniform identical background banding signal present in all tested genotypes generated by the polyclonal anti-*Pp*DEK1 CysPc-C2L antibody. Original TIFF whole versions of the blot and the gel are provided in the External Files part of the data archive deposited on Zenodo (`wetlab/gels_and_blots/Western/C2*.tif`).

S11e. PCR genotyping confirms genomic insertion in the *108* locus' 5' region. Primer pairs are indicated below the gel image. L: GeneRuler 1 kb DNA ladder, lanes 1, 3, 5, 7 WT DNA template, 2, 4, 6, 8 *oex1* DNA template. The specific amplification signal from the 5' flank of the construct in *oex1* (lane 4) but not in the WT (lane 3) and not from the 3' flank (lanes 6 and 8) points to the insertion of the *Pp*DEK1 Linker-Calpain construct at the 5' site of the 108 locus. The presence of a full *108* signal in *oex1 (*lane 2) indicates the presence of an intact *108* fragment probably associated with the aforementioned complex concatenated insertion.

S11f. Genomic mapping of RNASeq data confirms *DEK1* mutant genotypes and expected transcriptional outcomes. Integrative Genome Viewer[17] tracks displaying the coverage and alignments from a mapping of RNASeq data of the four *DEK1* mutants and the wild type (WT) at day 14 of gametophytic development with respect to the V3.3[21] gene

structure of *DEK1* (last track). Each of the RNASeq tracks is composed of two subtracks: 1) an upper subtrack displaying the coverage as a density plot. Peak minimum and maximum number of reads depicted in the upper left corner of the subtrack (e.g. *oex1* [0-18015]). 2) lower subtrack displays exemplary individual read alignments (color-coded by SAM alignment flags, non-gray colors usually indicating aberrant/low quality alignments). The last track shows the predicted gene structure of *DEK1* in genome annotation V3.3[21] color-coded by the domain architecture of the DEK1 protein depicted in S11g. Boxes depict exonic and connecting thin lines the intronic regions of the gene. Coverage histograms and read alignments are consistent with the expected genotype of the respective *DEK1* mutant, with reads lacking entirely or partially in the full (*Δdek1*[8]) or partial (*Δlg3*[18] and *Δloop*[19]) deletion lines as well as an over-accumulation of reads only in the regions encoding the Linker-Calpain domains in the *oex1* strain.

S11g. DEK1 protein domain structure. Overview of the DEK1 protein domain architecture. Individual domains and transmembrane regions are color-coded and annotated. Colored boxes represent conserved and/or functional domains and transmembrane regions, while the gray backbone depicts less conserved (spacer) regions.

S12a

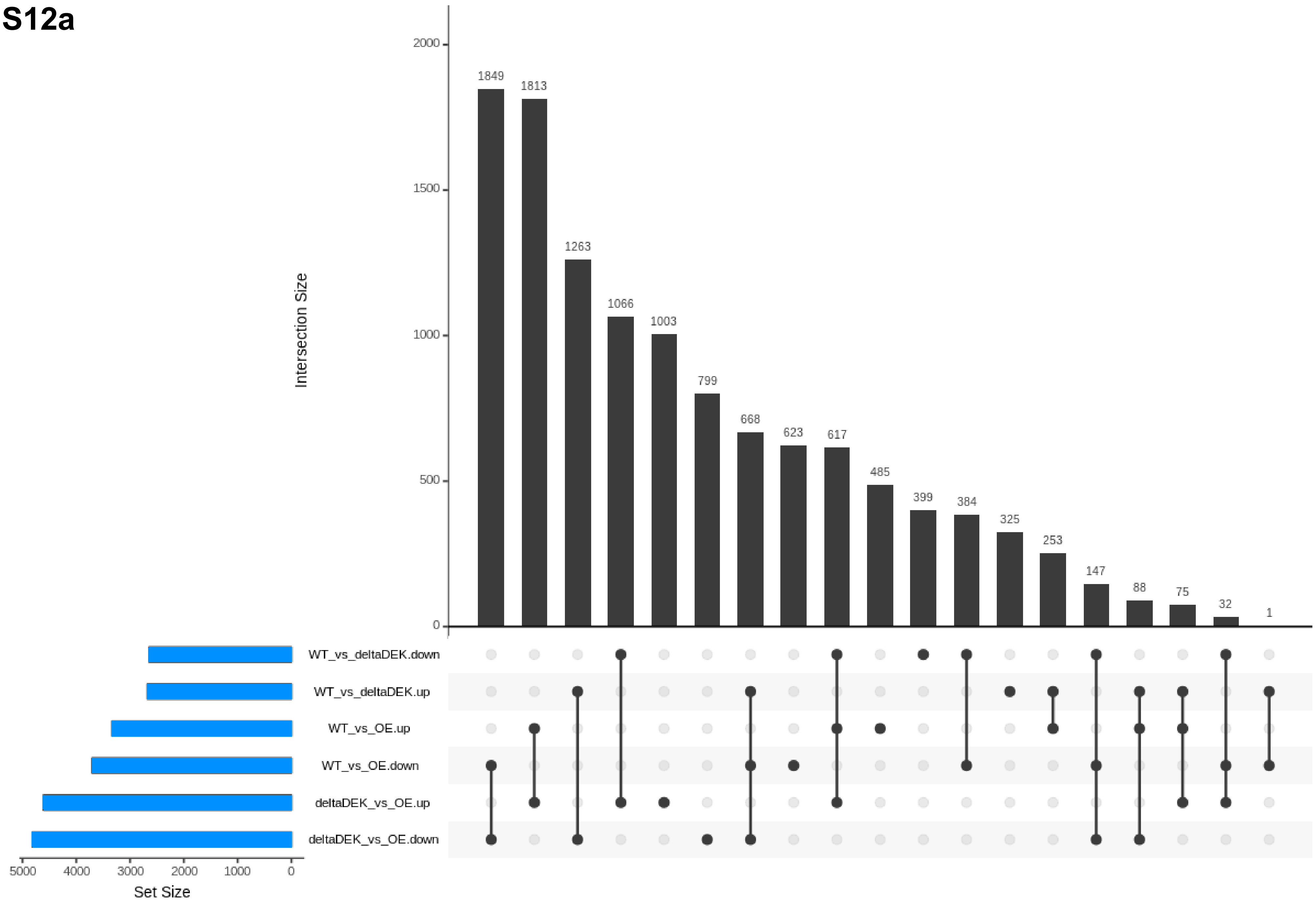
Intersection Size
2000
1500
1000
500
0
1849
1813
1263
1066
1003
799
668
623
617
485
399
384
325
253
147
88
75
32
1
WT_vs_deltaDEK.down
WT_vs_deltaDEK.up
WT_vs_OE.up
WT_vs_OE.down
deltaDEK_vs_OE.up
deltaDEK_vs_OE.down
5000
4000
3000
2000
1000
0
Set Size

S12b

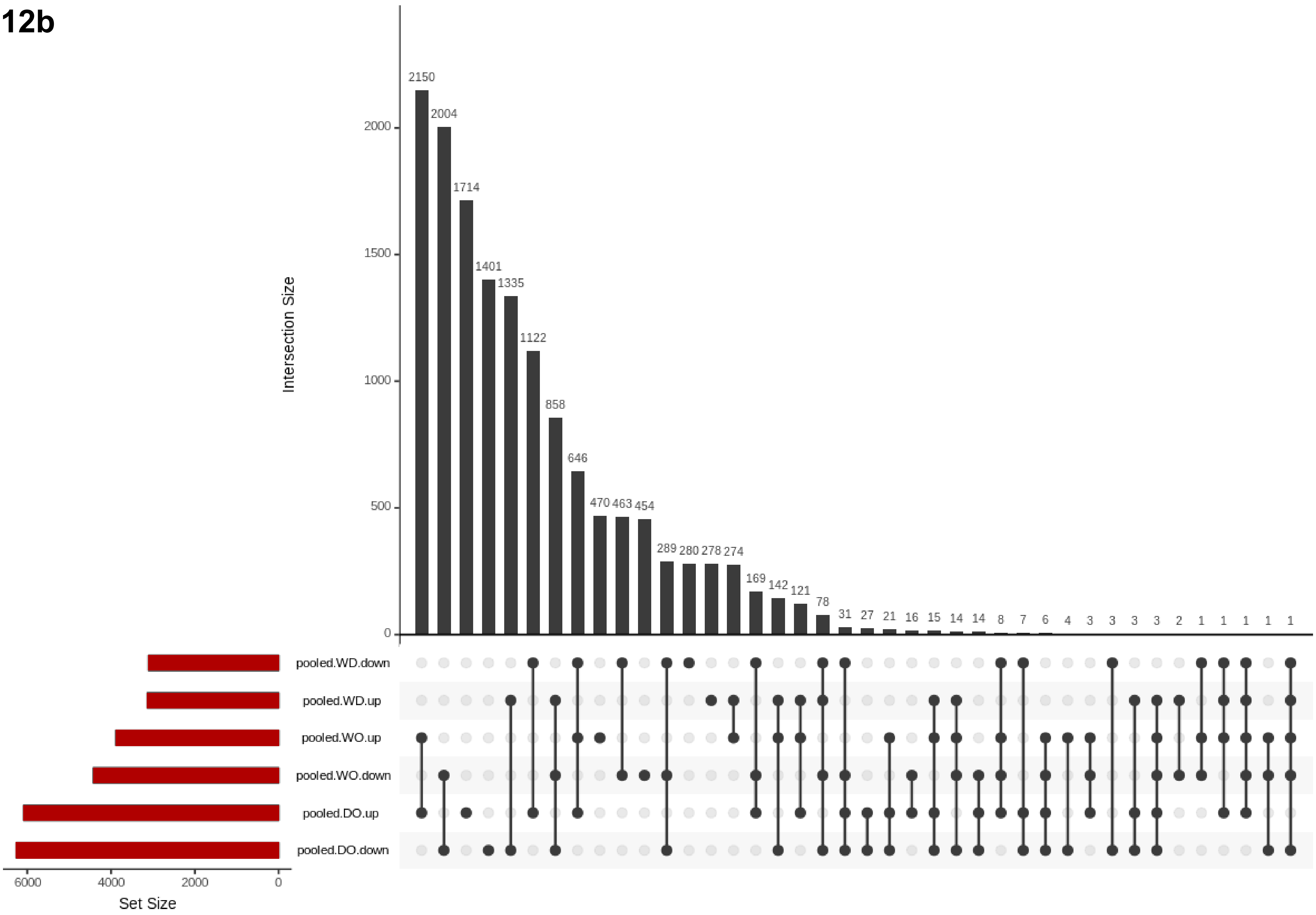
Intersection Size
2150
2004
1714
1401
1335
1122
858
646
470
463
454
289
280
278
274
169
142
121
78
31
27
21
16
15
14
14
8
7
6
4
3
3
3
3
2
1
1
1
1
1
0
500
1000
1500
2000
pooled.WD.down
pooled.WD.up
pooled.WO.up
pooled.WO.down
pooled.DO.up
pooled.DO.down
6000
4000
2000
0
Set Size

S12c

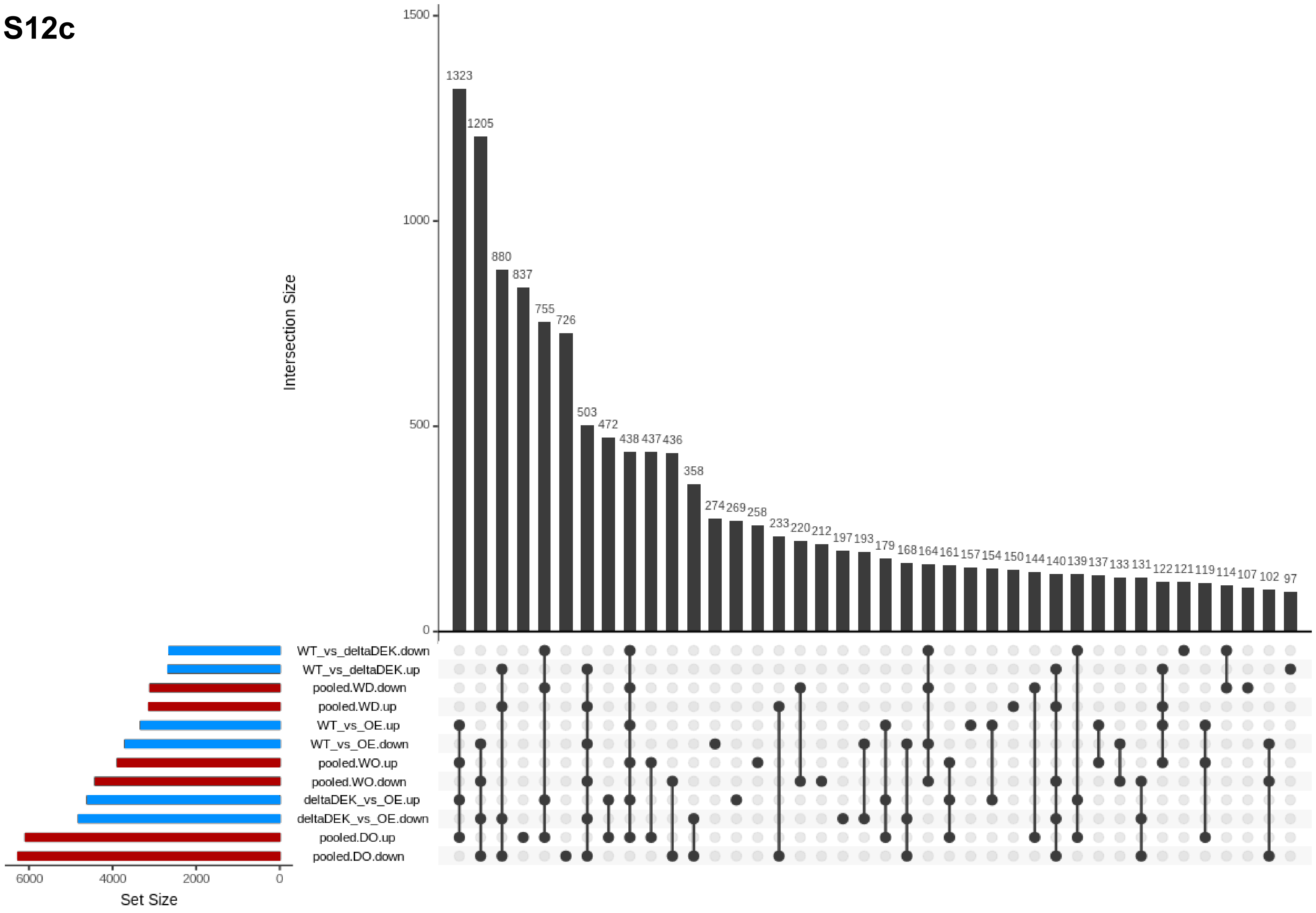
Intersection Size
1500
1000
500
0
1323
1205
880
837
755
726
503
472
438
437
436
358
274
269
258
233
220
212
197
193
179
168
164
161
157
154
150
144
140
139
137
133
131
122
121
119
114
107
102
97
WT_vs_deltaDEK.down
WT_vs_deltaDEK.up
pooled.WD.down
pooled.WD.up
WT_vs_OE.up
WT_vs_OE.down
pooled.WO.up
pooled.WO.down
deltaDEK_vs_OE.up
deltaDEK_vs_OE.down
pooled.DO.up
pooled.DO.down
6000
4000
2000
0
Set Size

S12d

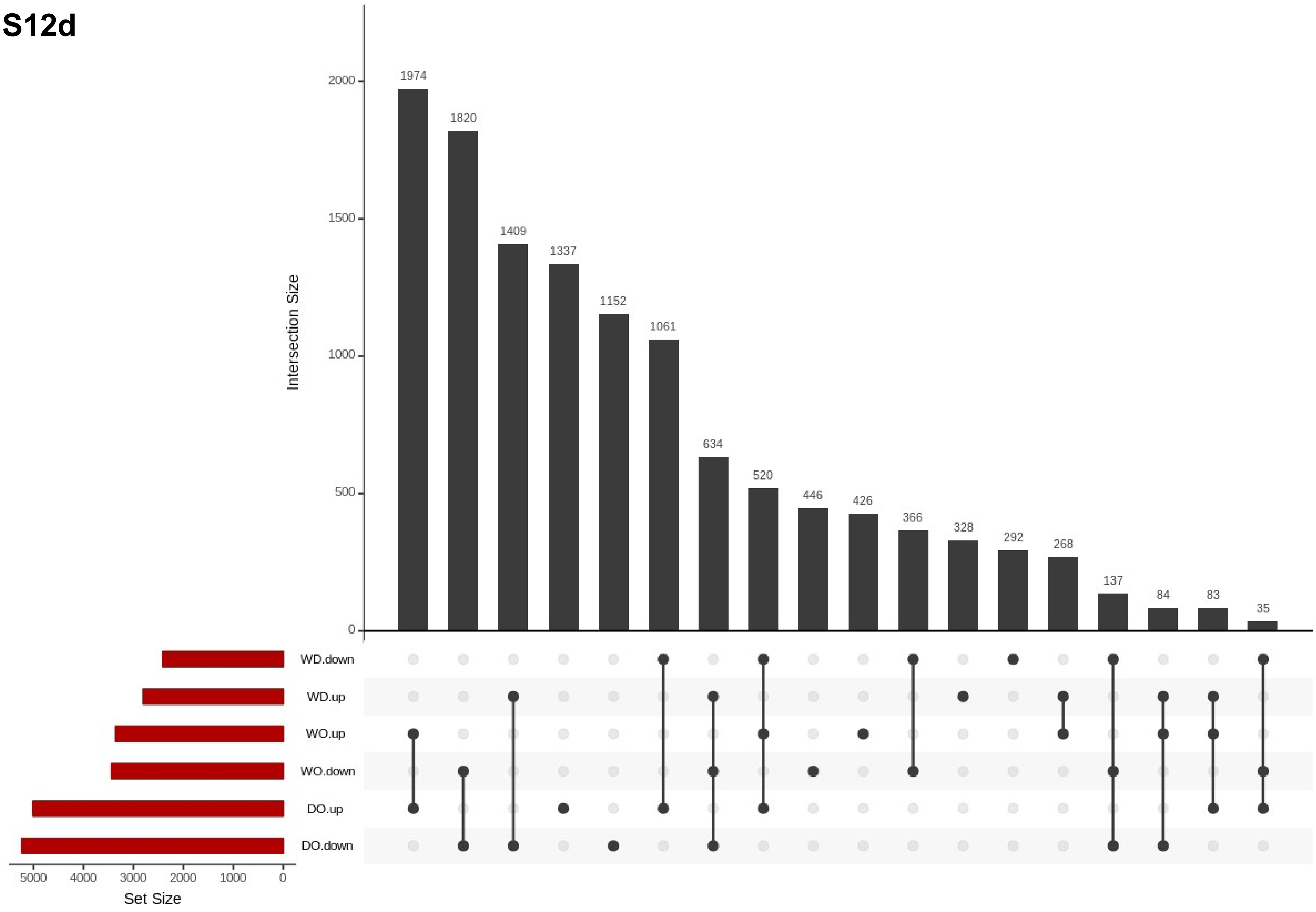

Intersection Size
2000
1500
1000
500
0
1974
1820
1409
1337
1152
1061
634
520
446
426
366
328
292
268
137
84
83
35
WD.down
WD.up
WO.up
WO.down
DO.up
DO.down
5000
4000
3000
2000
1000
0
Set Size

S12e

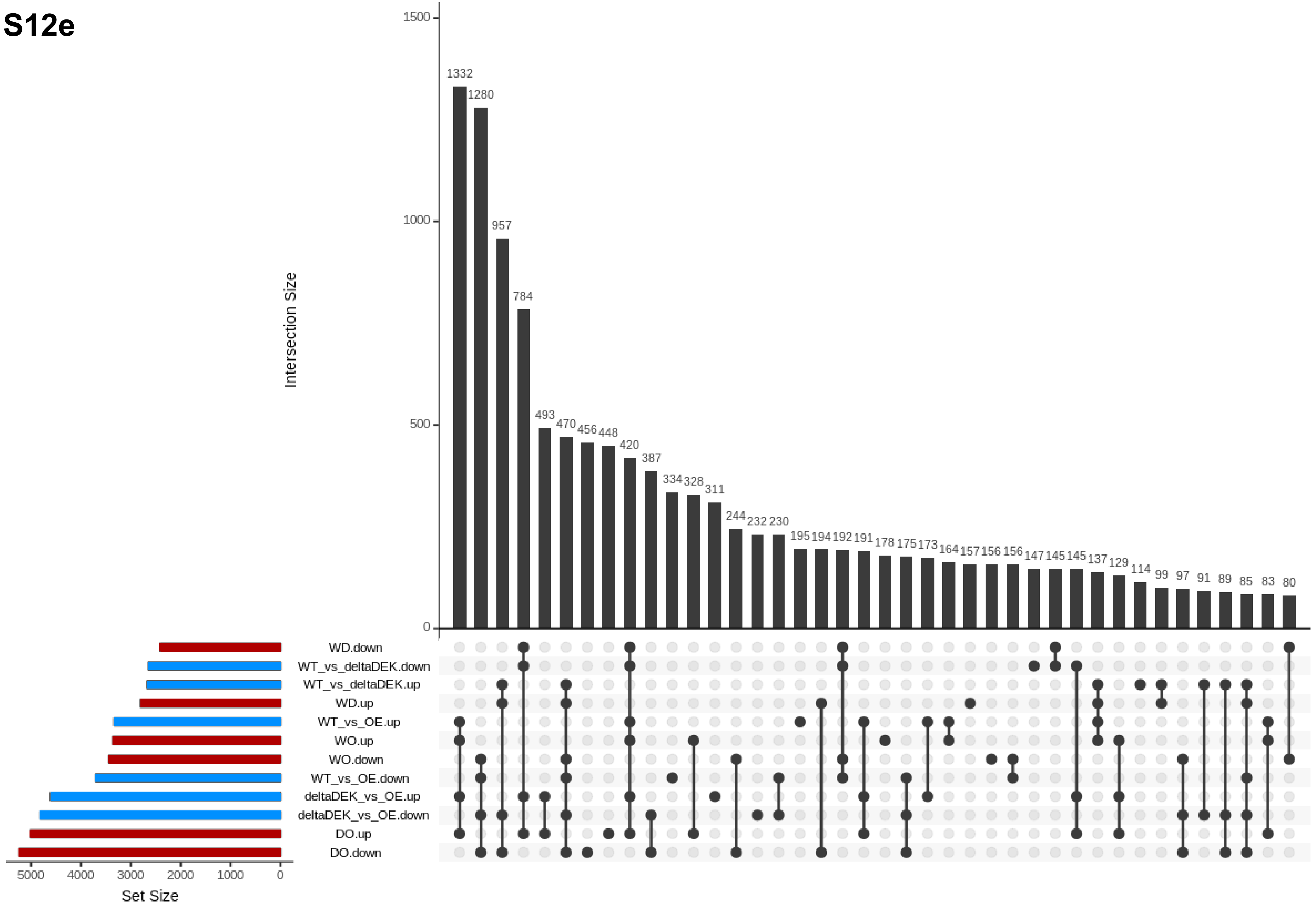

Intersection Size
1500
1000
500
0
1332
1280
957
784
493
470
456
448
420
387
334
328
311
244
232
230
195
194
192
191
178
175
173
164
157
156
156
147
145
145
137
129
114
99
97
91
89
85
83
80
WD.down
WT_vs_deltaDEK.down
WT_vs_deltaDEK.up
WD.up
WT_vs_OE.up
WO.up
WO.down
WT_vs_OE.down
deltaDEK_vs_OE.up
deltaDEK_vs_OE.down
DO.up
DO.down
5000
4000
3000
2000
1000
0
Set Size

Supplementary Fig. S12.

Comparison of differential gene expression (DGE) analysis results of *dek1* mutant lines using sleuth[2] vs. edgeR[20]. Global analysis of consistency between differentially expressed gene (DEG) sets. We compared the wild type (abbreviated in the subfigures as WT or W), *Δdek1*[8] (deltaDEK or D), *oex1 (*OE or O).

Several DGE approaches were assessed for there utility to analyze *dek1* mutant line time series comparisons. To illustrate the consistency among different approaches we include a comparison of sleuth and edgeR DGE analysis packages. As only sleuth supported time series modeling, we compared two strategies for edgeR: 1) pooling all time points per genotype and 2) testing time points individually and merging them prior to set analyses.

S12a. Overlap of DEG sets inferred using the sleuth DGE analysis package modeling genotype + timeseries + batch. UpSetR[35] plot depicting intersections and unique genes between up/down-regulated DEG sets comparing mutant lines and the wild type (WT) over time incorporating the error attributed due to RNA batch. Blue bars indicate the total DEG set size from DGE analysis using sleuth. Black bars indicate the size of the intersections/unique sets. Intersections are indicated by multiple, connected dots in the set matrix. Unique sets by only one dot.

S12b. Overlap of DEG sets inferred using the edgeR DGE analysis package - Strategy 1 modeling genotypes with pooled time points. Time point samples were analyzed as one pool per genotype as edgeR did not support time series modeling. UpSetR[35] plot depicting intersections and unique genes between up/down-regulated DEG sets. Red bars indicate total DEG set size from DGE analysis using sleuth. Black bars indicate the size of the intersections/unique sets. Intersections are indicated by multiple, connected dots in the set matrix. Unique sets by only one dot.

S12c. Comparison of sleuth vs. edgeR Strategy 1 DEG sets. UpSetR plot depicting intersections and unique genes between up/down-regulated DEG sets depicted in subfigure S12a (sleuth/blue color) vs. S12b (pooled edgeR/red color).

S11d. Overlap of DEG sets inferred using the edgeR DGE analysis package - Strategy 2 modeling genotypes with individual time points and subsequent merging of sets. Individual time point comparisons were carried out separately and subsequently merged for this intersection.

S12e. Comparison of sleuth vs. edgeR Strategy 2 DEG sets. UpSetR plot depicting intersections and unique genes between up/down-regulated DEG sets depicted in subfigure S12a (sleuth/blue color) vs. S12d (pooled edgeR/red color).

S13a

	1	2	3	4	5	6	7	8
L	WT	*oex1*	WT	*oex1*	WT	*oex1*	WT	*oex1*

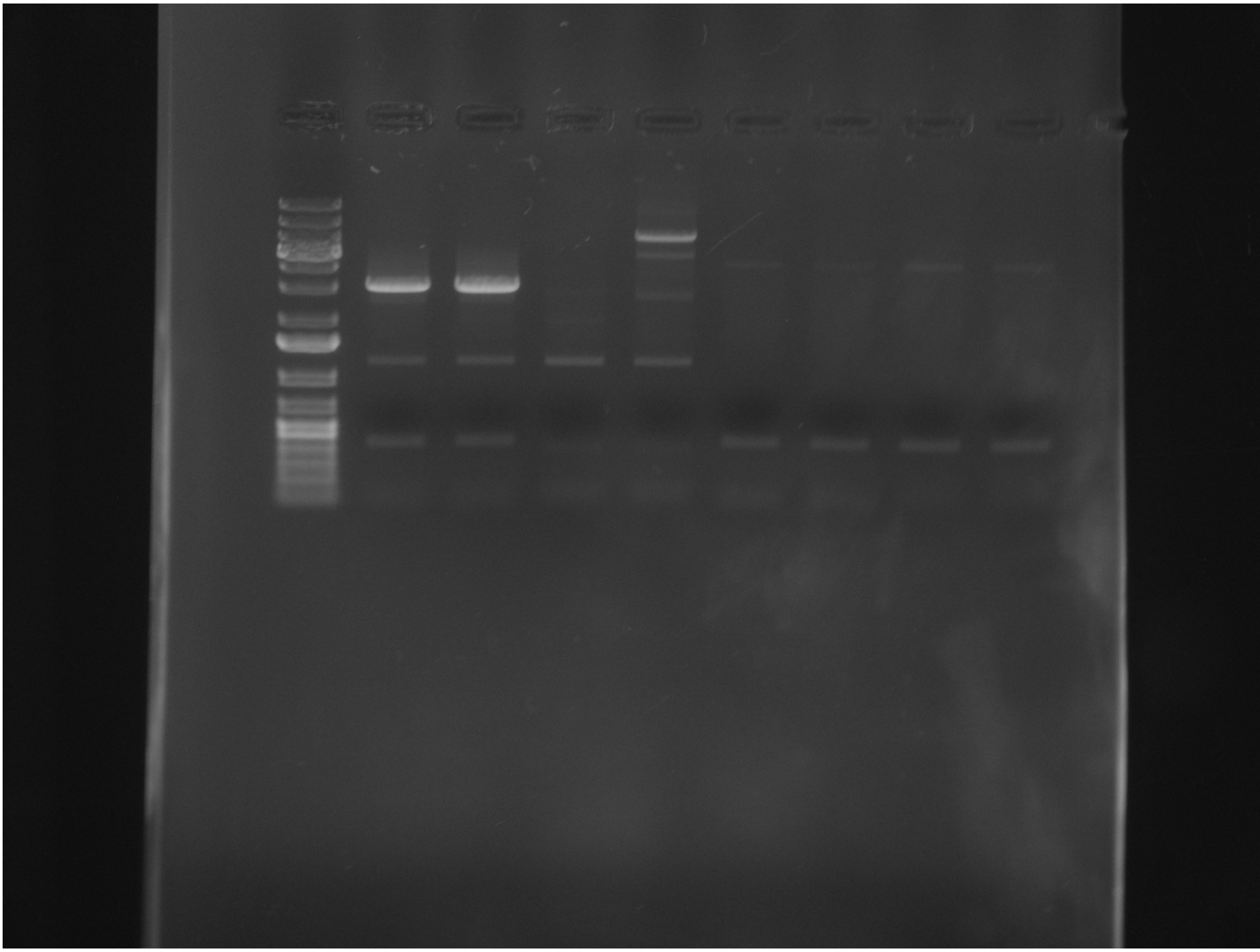

1, 2: 108_For – 108_Rev
3, 4: 108_For – 35S_Rev
5, 6: Term_For – 108_Rev
7, 8: Ubi_Rev – 108_Rev

S13b

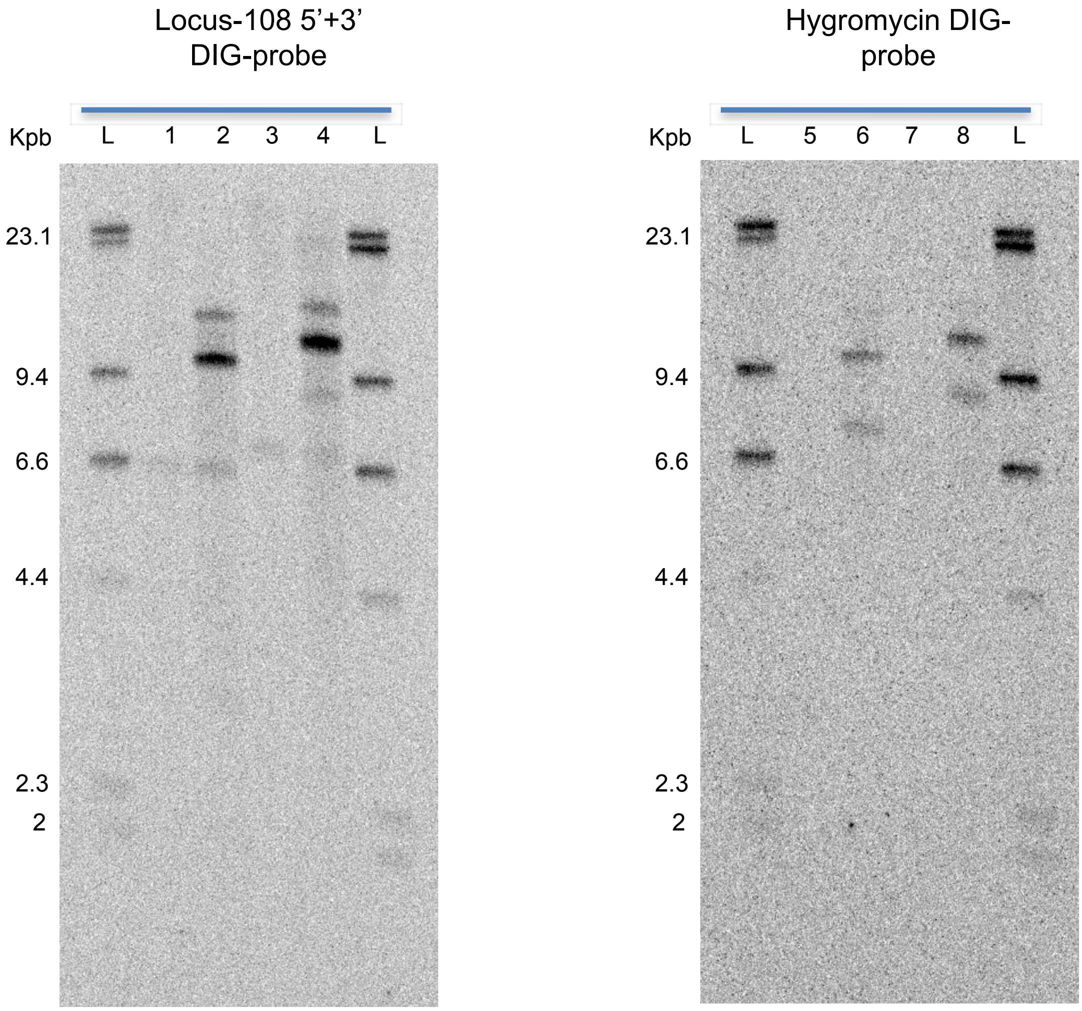

Locus-108 5'+3' DIG-probe
Kpb
L
1
2
3
4
L
23.1
9.4
6.6
4.4
2.3
2
Hygromycin DIG-probe
Kpb
L
5
6
7
8
L
23.1
9.4
6.6
4.4
2.3
2

S13c

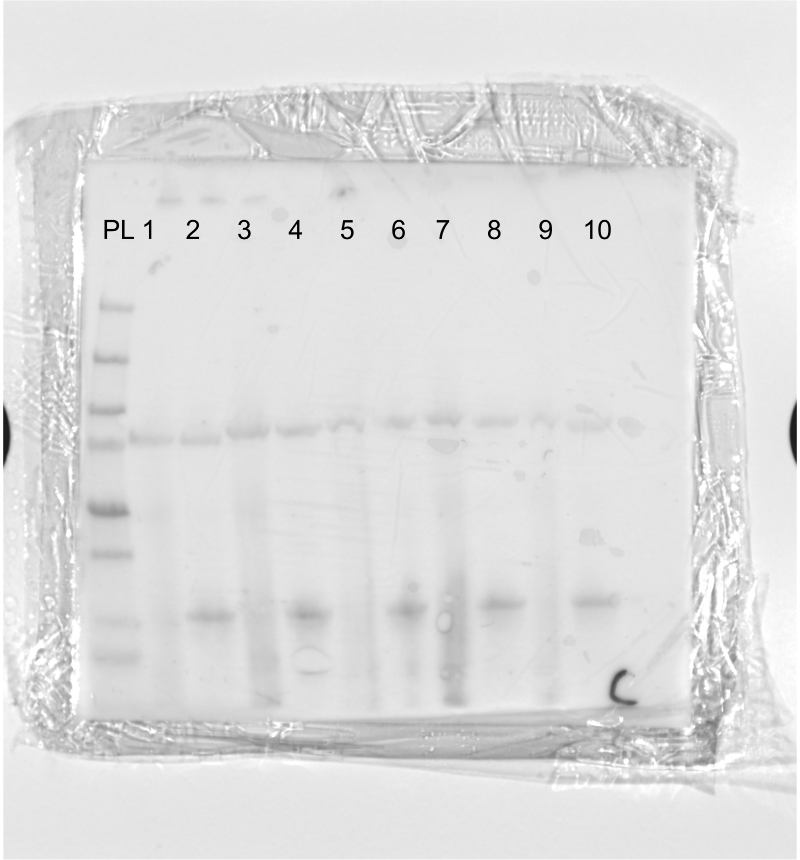

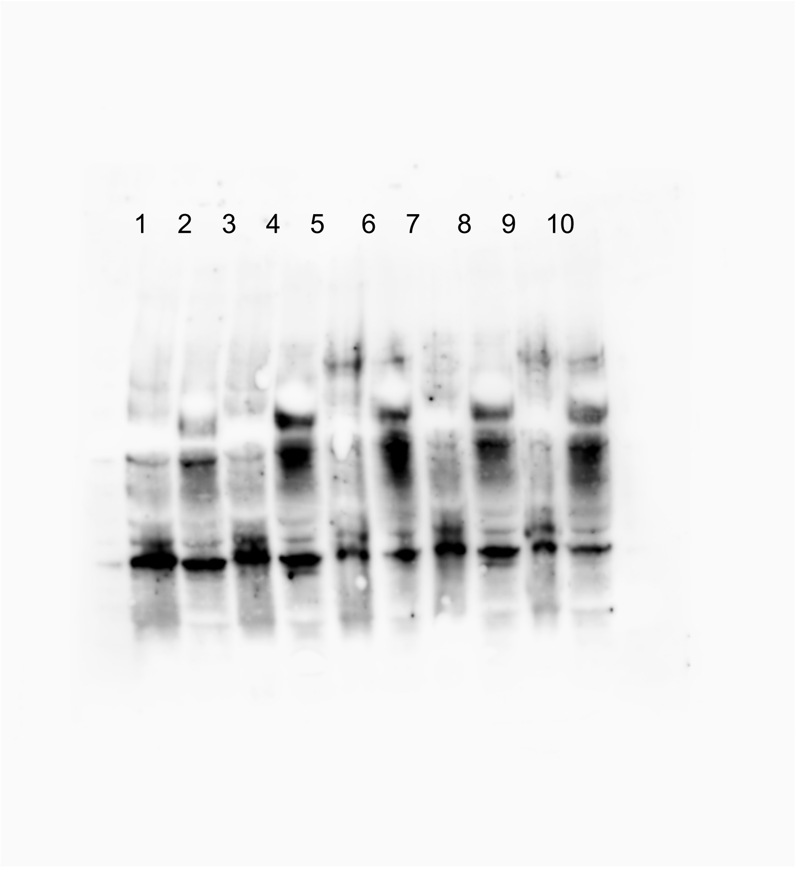

Supplementary Fig. S13.

Unedited gel and blot images.

S13a. PCR genotyping of the WT and *oex1* strain. Primer pairs used with DNA samples from individual strains are indicated below.

S13b. Southern blot analysis. Lanes 1,3,5, 7 wild type genomic DNA. Lane 2, 4, 6, 8 *oex1* genomic DNA. Restriction enzyme digests: lanes 1, 2, 5 and 6: Bgl II; lanes 3, 4, 7, 8,10: Xcm I. L: Lambda phage-HindIII DIG labeled marker.

S13c. Western blot analysis. Lines 1, 2, WT; 3, 4, 7, 8 *Δdek1;* 5, 6, 9, 10 *oex1*. Protein samples in lines 1, 3, 5, 7, 9 were heated before PAGE, samples 2, 4, 6 , 8, 10 were not heated before PAGE. PL, protein ladder.

Supplementary Data

Supplementary Data sheet S1. Summary table of the differential gene expression analysis of *DEK1* mutants.

Supplementary Data sheet S2. Summary table of overall deregulated biological processes, molecular functions, cellular components, anatomical entities and developmental stages in *DEK1* mutants as represented by the respective Gene Ontology (GO) and Plant Ontology (PO) partitions.

Supplementary Data sheet S3. Enriched ontology terms for the predicted *P. patens* gene regulatory subnetworks.

Supplementary Data sheet S4. Genes encoded by the five DEK1-controlled subnetworks.

Supplementary Data sheet S5. Summary table of the predicted regulatory interactions within and between the 11 subnetworks.

Supplementary Data sheet S6. Final filtered set of DEK1-controlled regulatory interactions.

Supplementary Data sheet S7. Enriched ontology terms for the filtered set of DEK1-controlled regulatory interactions (all targets and per subnetwork-pair/deregulation pattern type).

Supplementary Data sheet S8. Factorial Differential Gene Expression Network Enrichment Analysis (FDGENEA) of 17 phenotypic factors.

Supplementary Data sheet S9. Summary table of the DEK1-controlled regulatory interactions for *overbudding* associated genes.

Supplementary Data sheet S10. Full annotation of the genes associated with the *overbudding* phenotype (FDGENEA) including the cell type specific transcriptome data (Frank and Scanlon 2015; Figs 2a, b).

Supplementary Data sheet S11. Gene sets of the AP2 and MYB controlled circuits depicted in Figs. S10l, m.

Supplementary Data sheet S12. Primers used in this study.

Supplementary Data sheet S13. Key resources used in this study. Complete list of used antibodies, deposited data, experimental models/strains, software and algorithms including citations, sources, accession numbers and URLs.

Supplementary References

1. Perroud, P. F. et al. The *Physcomitrella patens* gene atlas project: large-scale RNA-seq based expression data. Plant J. 95, 168-182 (2018).

2. Pimentel, H., Bray, N. L., Puente, S., Melsted, P. & Pachter, L. Differential analysis of RNA-seq incorporating quantification uncertainty. Nat. Methods 14, 687-690 (2017).

3. Horstman, A., Willemsen, V., Boutilier, K. & Heidstra, R. AINTEGUMENTA-LIKE proteins: hubs in a plethora of networks. Trends Plant Sci. 19, 146-157 (2014).

4. Scheres, B. & Krizek, B. A. Coordination of growth in root and shoot apices by AIL/PLT transcription factors. Curr. Opin. Plant Biol. 41, 95-101 (2018).

5. Aoyama, T. et al. AP2-type transcription factors determine stem cell identity in the moss *Physcomitrella patens*. Development 139, 3120-3129 (2012).

6. Gil, D. P., Law, J. N. & Murali, T. M. The PathLinker app: Connect the dots in protein interaction networks. F1000Res 6, 58 (2017).

7. Shannon, P., Markiel, A., Ozier, O., Baliga, N. S., Wang, J.T., Ramage, D., Amin, N., Schwikowski, B., Ideker, T. Cytoscape: a software environment for integrated models of biomolecular interaction networks. Genome Res. 13, 2498-2504 (2003).

8. Perroud, P. F. et al. Defective Kernel 1 (DEK1) is required for three-dimensional growth in *Physcomitrella patens*. New Phytol. 203, 794-804 (2014).

9. Whitewoods, C. D. et al. CLAVATA was a genetic novelty for the morphological innovation of 3D growth in land plants. Curr. Biol. 28, 2365-2376 (2018).

10. Ishikawa, M. et al. *Physcomitrella* STEMIN transcription factor induces stem cell formation with epigenetic reprogramming. Nat. Plants 5, 681-690 (2019).

11. Chandler, J. W. & Werr, W. DORNRÖSCHEN, DORNRÖSCHEN-LIKE, and PUCHI redundantly control floral meristem identity and organ initiation in Arabidopsis. J. Exp. Bot. 68, 3457-3472 (2019).

12. Aya, K. et al. The Gibberellin perception system evolved to regulate a pre-existing GAMYB-mediated system during land plant evolution. Nat. Commun. 2, 544 (2011).

13. Xu, Y., Iacuone, S., Li, S. F. & Parish, R. W. MYB80 homologues in Arabidopsis, cotton and Brassica: regulation and functional conservation in tapetal and pollen development. BMC Plant Biol. 14, 278 (2014).

14. Oshima, Y. et al. MIXTA-like transcription factors and WAX INDUCER1/SHINE1 coordinately regulate cuticle development in Arabidopsis and Torenia fournieri. Plant Cell 25, 1609-1624 (2013).

15. Schaefer, D. G. & Zrÿd, J. P. Efficient gene targeting in the moss *Physcomitrella patens*. Plant J. 11, 1195-1206 (1997).

16. Kamisugi, Y., Mitsuya, S., El-Shami, M., Knight, C. D., Cuming, A. C., Baker, A. Giant peroxisomes in a moss (*Physcomitrella patens*) peroxisomal biogenesis factor 11 mutant. New Phytol. 209, 576-589 (2016).

17. Robinson, J. T., Thorvaldsdóttir, H., Winckler, W., Guttman, M., Lander, E. S., Getz, G., Mesirov, J. P. Integrative genomics viewer. Nat. Biotechnol. 29, 24-26 (2011).

18. Johansen, W. et al. The DEK1 calpain Linker functions in three-dimensional body pattering in *Physcomitrella patens*. Plant Physiol. 172, 1089-1104 (2016).

19. Demko, V. et al. Genetic analysis of DEK1 Loop function in three-dimensional body patterning in *Physcomitrella patens*. Plant Physiol. 166, 903-919 (2014).

20. Robinson M. D., McCarthy D. J., Smyth, G. K. edgeR: a Bioconductor package for differential expression analysis of digital gene expression data. Bioinformatics 26, 139-140 (2010).

21. Lang, D. et al. The *Physcomitrella patens* chromosome-scale assembly reveals moss genome structure and evolution. Plant J. 93, 515-533 (2018).

22. Cooper, L. et al. The plant ontology as a tool for comparative plant anatomy and genomic analyses. Plant Cell Physiol. 54, e1 (2013).

23. Zimmer, A. D. et al. Reannotation and extended community resources for the genome of the non-seed plant *Physcomitrella patens* provide insights into the evolution of plant gene structures and functions. BMC Genomics 14, 498 (2013).

24. Fu, L., Niu, B., Zhu, Z., Wu, S. & Li, W. CD-HIT: accelerated for clustering the next-generation sequencing data. Bioinformatics 28, 3150-3152 (2012).

25. Pertea, G. & Pertea, M. GFF Utilities: GffRead and GffCompare. F1000Res 28, ISCB Comm. J-304 (2020).

26. Mölder, F., Jablonski, K.P., Letcher, B., Hall, M.B., Tomkins-Tinch, C.H., Sochat, V., Forster, J., Lee, S., Twardziok, S.O., Kanitz, A., Wilm, A., Holtgrewe, M., Rahmann, S., Nahnsen, S., Köster, J. Sustainable data analysis with Snakemake. F1000Res 10, 33 (2021).

28. Briesemeister, S., Rahnenführer, J. & Kohlbacher, O. Going from where to why--interpretable prediction of protein subcellular localization. Bioinformatics 26, 1232-1238 (2010).

29. Krogh, A., Larsson, B., von Heijne, G. & Sonnhammer, E. L. Predicting transmembrane protein topology with a hidden Markov model: application to complete genomes. J. Mol. Biol. 305, 567-580 (2001).

30. Jones D. T. Improving the accuracy of transmembrane protein topology prediction using evolutionary information. Bioinformatics 23, 538-544 (2007).

31. Emms, D. M. & Kelly, S. OrthoFinder: solving fundamental biases in whole genome comparisons dramatically improves orthogroup inference accuracy. Genome Biol. 16, 157 (2015).

32. Andrews, S. FastQC: a qualiupsetrty control tool for high throughput sequence data.https://www.bioinformatics.babraham.ac.uk/projects/fastqc/ (2010).

33. Bolger, A. M., Lohse, M. & Usadel, B. Trimmomatic: a flexible trimmer for Illumina sequence data. Bioinformatics 30, 2114-2120 (2014).

34. Kluyver, T. et al. Jupyter Notebooks – a publishing format for reproducible computational workflows. In F. Loizides & B. Schmidt, eds. Positioning and Power in Academic Publishing: Players, Agents and Agendas. 87–90 (2016).

35. Conway, J. R., Lex, A. & Gehlenborg, N. UpSetR: an R package for the visualization of intersecting sets and their properties. Bioinformatics 33, 2938-2940 (2017).

36. Kucera, M., Isserlin, R., Arkhangorodsky, A. & Bader, G. D. AutoAnnotate: A Cytoscape app for summarizing networks with semantic annotations. F1000Res 5, 1717 (2016).

37. Scrucca, L., Fop, M., Murphy, T. B. & Raftery, A. E. mclust 5: Clustering, Classification and Density Estimation Using Gaussian Finite Mixture Models. R. J. 8, 289-317 (2016).
