# Supplementary material for: Regulation of developmental gatekeeping and cell fate transition by the calpain protease DEK1 in Physcomitrium patens
Source: Commun Biol. 2024 Mar 4;7:261. doi: 10.1038/s42003-024-05933-z (PMC10912778; doi:10.1038/s42003-024-05933-z)
Supplement: Supplementary file 3 — Description of Additional Supplementary Files [file 42003_2024_5933_MOESM3_ESM.pdf]

Description of Additional Supplementary Files

File name: Supplementary Data S1
Description: Summary table of the differential gene expression analysis of DEK1 mutants.

File name: Supplementary Data S2
Description: Summary table of overall deregulated biological processes, molecular functions, cellular components, anatomical entities and developmental stages in DEK1 mutants as represented by the respective Gene Ontology (GO) and Plant Ontology (PO) partitions.

File name: Supplementary Data S3
Description: Enriched ontology terms for the predicted P. patens gene regulatory subnetworks.

File name: Supplementary Data S4
Description: Genes encoded by the five DEK1-controlled subnetworks

File name: Supplementary Data S5
Description: Summary table of the predicted regulatory interactions within and between the 11 subnetworks.

File name: Supplementary Data S6
Description: Final filtered set of DEK1-controlled regulatory interactions

File name: Supplementary Data S7
Description: Enriched ontology terms for the filtered set of DEK1- controlled regulatory interactions (all targets and per subnetwork-pair/deregulation pattern type).

File name: Supplementary Data S8
Description: Factorial Differential Gene Expression Network Enrichment Analysis (FDGENEA) of 17 phenotypic factors.

File name: Supplementary Data S9
Description: Summary table of the DEK1-controlled regulatory interactions for overbudding associated genes

File name: Supplementary Data S10
Description: Full annotation of the genes associated with the overbudding phenotype (FDGENEA) including the cell type specific transcriptome data (Frank and Scanlon 2015; Figs 2a, b).

File name: Supplementary Data S11
Description: Gene sets of the AP2 and MYB controlled circuits depicted in Figs. S10l, m.

File name: Supplementary Data S12
Description: Primers used in this study.

File name: Supplementary Data S13
Description: Key resources used in this study. Complete list of used antibodies, deposited data, experimental models/strains, software and algorithms including citations, sources, accession numbers and URLs.
