# Supplementary material for: Regulation of developmental gatekeeping and cell fate transition by the calpain protease DEK1 in Physcomitrium patens
Source: Commun Biol. 2024 Mar 4;7:261. doi: 10.1038/s42003-024-05933-z (PMC10912778; doi:10.1038/s42003-024-05933-z)
Supplement: Supplementary file 5 — Reporting Summary [file 42003_2024_5933_MOESM5_ESM.pdf]

nature portfolio

Corresponding author(s): Daniel Lang

Last updated by author(s): Jan 28, 2024

Reporting Summary

Nature Portfolio wishes to improve the reproducibility of the work that we publish. This form provides structure for consistency and transparency in reporting. For further information on Nature Portfolio policies, see our Editorial Policies and the Editorial Policy Checklist.

Statistics

For all statistical analyses, confirm that the following items are present in the figure legend, table legend, main text, or Methods section.

n/a	Confirmed	
☐	☒	The exact sample size (*n*) for each experimental group/condition, given as a discrete number and unit of measurement
☐	☒	A statement on whether measurements were taken from distinct samples or whether the same sample was measured repeatedly
☐	☒	The statistical test(s) used AND whether they are one- or two-sided<br>*Only common tests should be described solely by name; describe more complex techniques in the Methods section.*
☐	☒	A description of all covariates tested
☐	☒	A description of any assumptions or corrections, such as tests of normality and adjustment for multiple comparisons
☐	☒	A full description of the statistical parameters including central tendency (e.g. means) or other basic estimates (e.g. regression coefficient) AND variation (e.g. standard deviation) or associated estimates of uncertainty (e.g. confidence intervals)
☐	☒	For null hypothesis testing, the test statistic (e.g. *F*, *t*, *r*) with confidence intervals, effect sizes, degrees of freedom and *P* value noted<br>*Give P values as exact values whenever suitable.*
☒	☐	For Bayesian analysis, information on the choice of priors and Markov chain Monte Carlo settings
☐	☒	For hierarchical and complex designs, identification of the appropriate level for tests and full reporting of outcomes
☐	☒	Estimates of effect sizes (e.g. Cohen's *d*, Pearson's *r*), indicating how they were calculated

Our web collection on statistics for biologists contains articles on many of the points above.

Software and code

Policy information about availability of computer code

Data collection	Code availability<br>● All original code has been deposited to git repositories. Parallelized Snakemake workflows are provided as individual repositories. Data analyses, statistics and visualizations were implemented via R or Python Jupyter Notebooks and for convenience are also accessible via a GitHub repository (https://github.com/dandaman/moss_DEK1_GRN_analysis). All git repositories have been pushed to GitHub and deposited at Zenodo and are publicly available. DOIs are listed Supplementary Data sheet S13.<br>● Used packaged software are provided via conda environments included in the Zenodo archive listed Supplementary Data sheet S13. File names of the environments correspond to the Jupyter kernels of each notebook.
Data analysis	Code availability<br>● All original code has been deposited to git repositories. Parallelized Snakemake workflows are provided as individual repositories. Data analyses, statistics and visualizations were implemented via R or Python Jupyter Notebooks and for convenience are also accessible via a GitHub repository (https://github.com/dandaman/moss_DEK1_GRN_analysis). All git repositories have been pushed to GitHub and deposited at Zenodo and are publicly available. DOIs are listed Supplementary Data sheet S13.<br>● Used packaged software are provided via conda environments included in the Zenodo archive listed Supplementary Data sheet S13. File names of the environments correspond to the Jupyter kernels of each notebook.

For manuscripts utilizing custom algorithms or software that are central to the research but not yet described in published literature, software must be made available to editors and reviewers. We strongly encourage code deposition in a community repository (e.g. GitHub). See the Nature Portfolio guidelines for submitting code & software for further information.

Data

Policy information about availability of data

All manuscripts must include a data availability statement. This statement should provide the following information, where applicable:

- Accession codes, unique identifiers, or web links for publicly available datasets
- A description of any restrictions on data availability
- For clinical datasets or third party data, please ensure that the statement adheres to our policy

Data and materials availability:
● The Physcomitrium patens line (oex1) generated in this study as well as other P. patens lines used have been deposited at Comenius University in Bratislava, Department of Plant Physiology moss collection, and are listed in Supplementary Data sheet S13.
● RNA-seq data have been deposited at EBI Array Express and are publicly available as of the date of publication (E-MTAB-10907).
● All generated data sets have been deposited at Zenodo https://doi.org/10.5281/zenodo.5513495
● This paper analyzes existing, publicly available data. A table with all accession numbers for public datasets is provided in Supplementary Data sheet S13.
● Raw images generated in this study, including microscopy, gel and immunoblot images, are publicly available as part of the Zenodo archive and listed Supplementary Data sheet S13.
● All 27 P. patens gene sets used in the figures or the text are provided as geneid lists in plain text files in the gene_sets/ folder of the Zenodo archive listed Supplementary Data sheet S13.
● Postgresql table dumps, as well as additional .tsv/.csv tables that are not explicitly mentioned in the text below but are used in the Jupyter notebooks, are provided in the Zenodo archive listed Supplementary Data sheet S13.

Research involving human participants, their data, or biological material

Policy information about studies with human participants or human data. See also policy information about sex, gender (identity/presentation), and sexual orientation and race, ethnicity and racism.

Reporting on sex and gender	N/A
Reporting on race, ethnicity, or other socially relevant groupings	N/A
Population characteristics	N/A
Recruitment	N/A
Ethics oversight	N/A

Note that full information on the approval of the study protocol must also be provided in the manuscript.

Field-specific reporting

Please select the one below that is the best fit for your research. If you are not sure, read the appropriate sections before making your selection.

☒ Life sciences ☐ Behavioural & social sciences ☐ Ecological, evolutionary & environmental sciences

For a reference copy of the document with all sections, see nature.com/documents/nr-reporting-summary-flat.pdf

Life sciences study design

All studies must disclose on these points even when the disclosure is negative.

Sample size	Plant material: five strains of Physcomitrium patens were used including wild type plants and four strains carrying specific mutations in the gene of interest DEK1. To quantify bud initiation, 100 filaments from each strain were analyzed as described in Material and Methods section of the manuscript.
Data exclusions	No data were excluded for phenotypic analysis of the model plant Physcomitrium patens.
Replication	For RNAseq analysis we used five strains of Physcomitrium patens. RNA was extracted from each strain at five time points. Three biological replicates were used for each strain/time point, which gives 75 samples for RNA library construction. One sample was damaged during the process (one replicate of the oex1 strain at 3 days time point) giving 74 RNAseq libraries used in total.
Randomization	Experimental plants were placed in the growth chamber in a way to achieve random position of each strain/biological replicate. For statistical and other data science procedures we applied a common random seed were applicable for the final figures and discussed results, but where feasible, compared results using different random seeds.
Blinding	Where feasible or relevant to the study design, we applied blinding during data collection and analysis.

Reporting for specific materials, systems and methods

We require information from authors about some types of materials, experimental systems and methods used in many studies. Here, indicate whether each material, system or method listed is relevant to your study. If you are not sure if a list item applies to your research, read the appropriate section before selecting a response.

Materials & experimental systems

n/a	Involved in the study
☐	☒ Antibodies
☒	☐ Eukaryotic cell lines
☒	☐ Palaeontology and archaeology
☒	☐ Animals and other organisms
☒	☐ Clinical data
☒	☐ Dual use research of concern
☐	☒ Plants

Methods

n/a	Involved in the study
☒	☐ ChIP-seq
☒	☐ Flow cytometry
☒	☐ MRI-based neuroimaging

Antibodies

Antibodies used	anti-CysPc to detect P. patens DEK1 Calpain epitope, polyclonal produced in rabbit, peptide sequence: WSRPEEVLREQGQDC, GenScript), Goat Anti-Rabbit IgG (H + L)-HRP Conjugate (Bio-Rad) was used as secondary antibody.
Validation	The primary antibody was validated by Western blot.

Plants

Seed stocks	Physcomitrium patens (Gransden) strain was used from the collection of the Norwegian University of Life Sciences, Norway.
Novel plant genotypes	Mutant Physcomitrium patens plants were generated using homologous recombination mutagenesis as described in details in the Material and Methods section. PEG-mediated protoplast transformation was used. Generation and characterization of the mutant lines (delta-dek1, delta-loop, delta-lg3) used in this study was previously published as referred. The DEK1 calpain overexpressing strain was generated for this study as described in detail in Material and Methods section.
Authentication	All mutant strains used were characterized by PCR genotyping and Southern blot genotyping. In addition the oex1 strain used in this study was validated using Western blot analysis. The phenotype, growth and development of each strain was assessed using microscopic methods as described in detail in Material and Methods section of the manuscript.
